# Beyond Bar and Line Graphs: Time for a New Data Presentation Paradigm

**DOI:** 10.1371/journal.pbio.1002128

**Published:** 2015-04-22

**Authors:** Tracey L. Weissgerber, Natasa M. Milic, Stacey J. Winham, Vesna D. Garovic

**Affiliations:** 1 Division of Nephrology & Hypertension, Mayo Clinic, Rochester, Minnesota, United States of America; 2 Department of Biostatistics, Medical Faculty, University of Belgrade, Belgrade, Serbia; 3 Division of Biomedical Statistic and Informatics, Mayo Clinic, Rochester, Minnesota, United States of America

## Abstract

Figures in scientific publications are critically important because they often show the data supporting key findings. Our systematic review of research articles published in top physiology journals (*n* = 703) suggests that, as scientists, we urgently need to change our practices for presenting continuous data in small sample size studies. Papers rarely included scatterplots, box plots, and histograms that allow readers to critically evaluate continuous data. Most papers presented continuous data in bar and line graphs. This is problematic, as many different data distributions can lead to the same bar or line graph. The full data may suggest different conclusions from the summary statistics. We recommend training investigators in data presentation, encouraging a more complete presentation of data, and changing journal editorial policies. Investigators can quickly make univariate scatterplots for small sample size studies using our Excel templates.

## Abstract 2

A systematic review of research articles reveals widespread poor practice in the presentation of continuous data. The authors recommend training for investigators and supply templates for easy use.

## Introduction

Data presentation is the foundation of our collective scientific knowledge, as readers’ understanding of a dataset is generally limited to what the authors present in their publications. Figures are critically important because they often show the data that support key findings. However, studies of the Journal of the American Medical Association [1] and the British Medical Journal [2] provide compelling evidence that fundamental changes in the types of figures that scientists use are needed. Authors generally use figures to present summary statistics, instead of providing detailed information about the distribution of the data or showing the full data [1,2].

**Fig 1:**
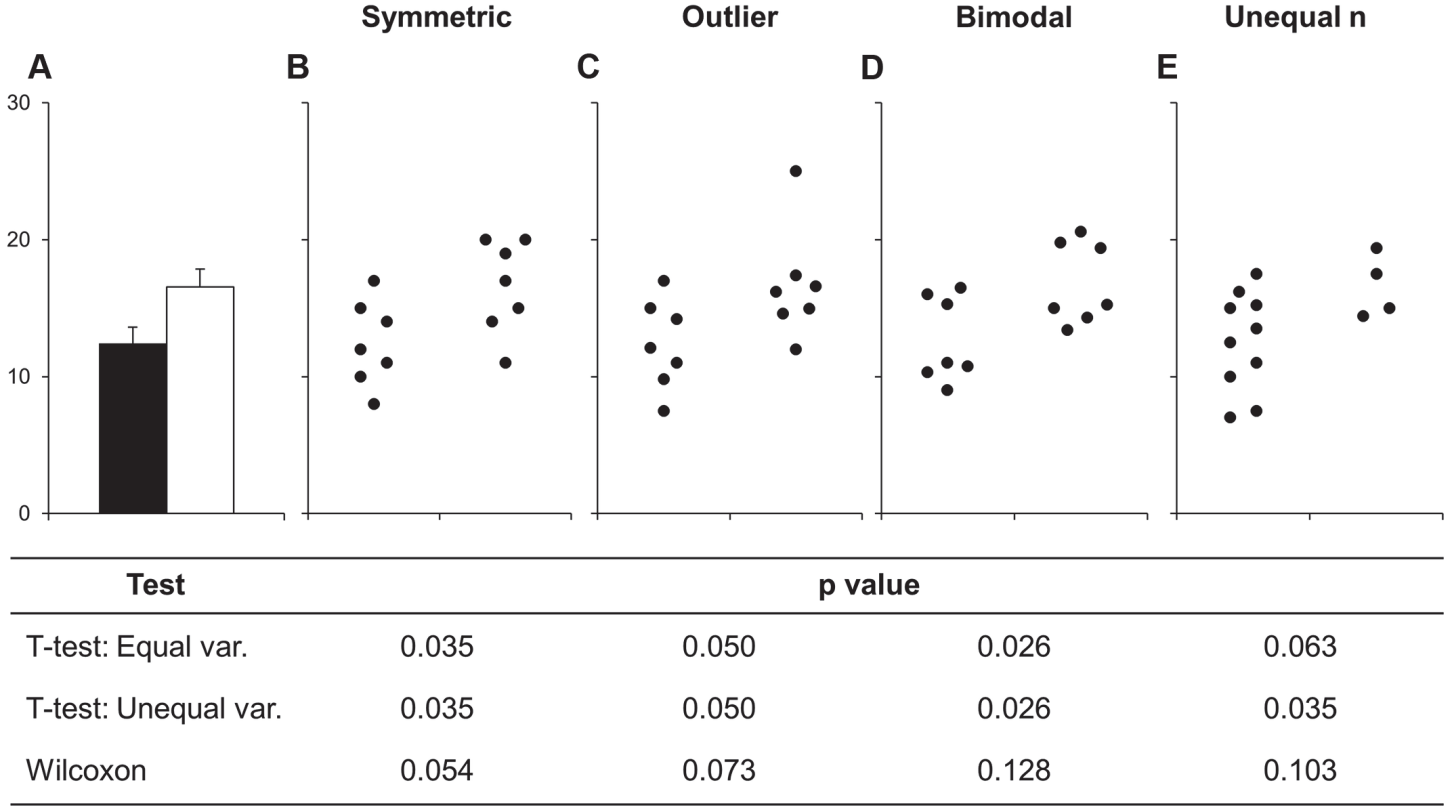
Many different datasets can lead to the same bar graph. The full data may suggest different conclusions from the summary statistics. The means and SEs for the four example datasets shown in Panels B–E are all within 0.5 units of the means and SEs shown in the bar graph (Panel A). *p*-values were calculated in R (version 3.0.3) using an unpaired t-test, an unpaired t-test with Welch’s correction for unequal variances, or a Wilcoxon rank sum test. In Panel B, the distribution in both groups appears symmetric. Although the data suggest a small difference between groups, there is substantial overlap between groups. In Panel C, the apparent difference between groups is driven by an outlier. Panel D suggests a possible bimodal distribution. Additional data are needed to confirm that the distribution is bimodal and to determine whether this effect is explained by a covariate. In Panel E, the smaller range of values in group two may simply be due to the fact that there are only three observations. Additional data for group two would be needed to determine whether the groups are actually different.

**Fig 2:**
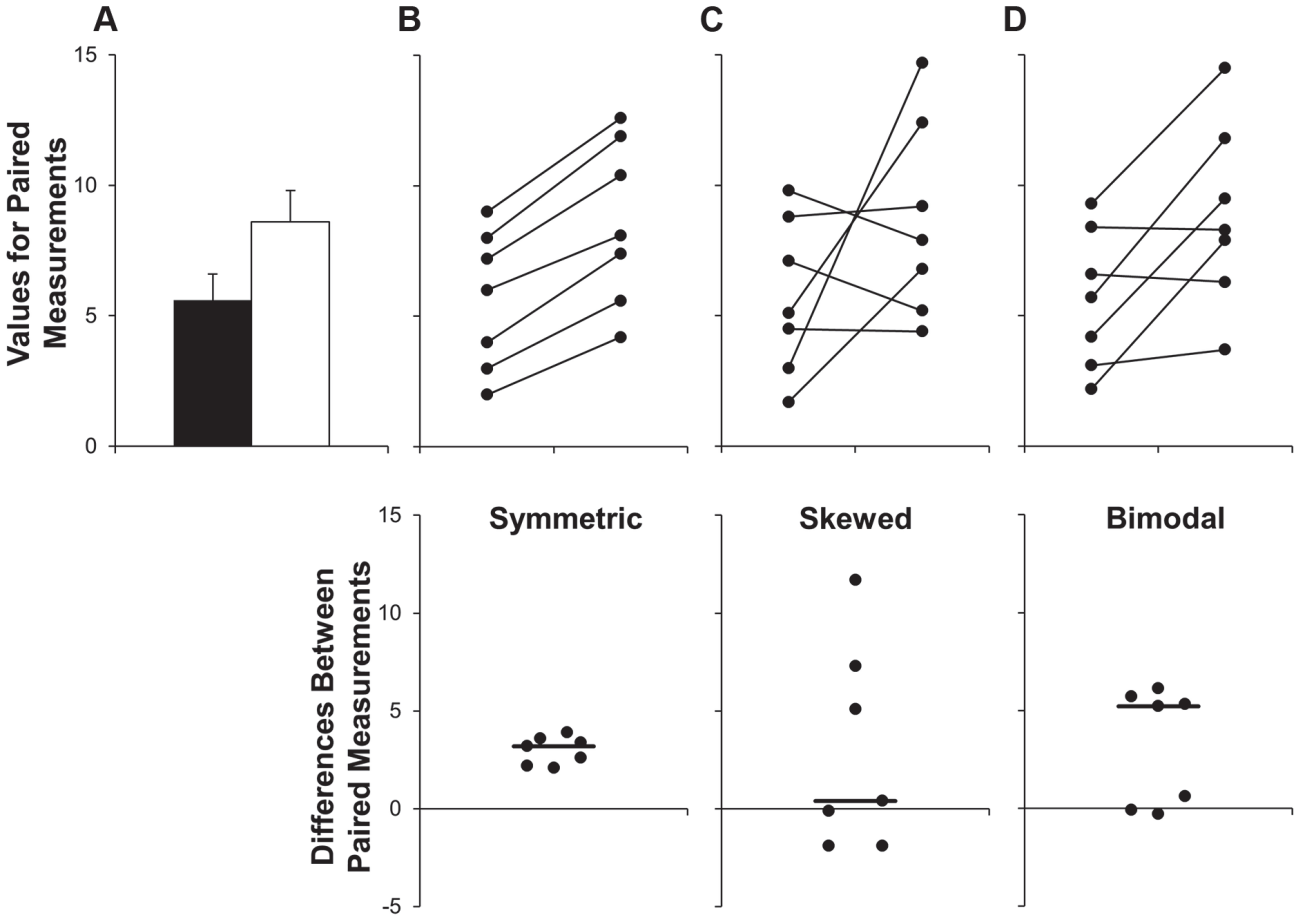
Additional problems with using bar graphs to show paired data. The bar graph (mean ± SE) suggests that the groups are independent and provides no information about whether changes are consistent across individuals (Panel A). The scatterplots shown in the Panels B–D clearly demonstrate that the data are paired. Each scatterplot reveals very different patterns of change, even though the means and SEs differ by less than 0.3 units. The lower scatterplots showing the differences between measurements allow readers to quickly assess the direction, magnitude, and distribution of the changes. The solid lines show the median difference. In Panel B, values for every subject are higher in the second condition. In Panel C, there are no consistent differences between the two conditions. Panel D suggests that there may be distinct subgroups of “responders” and “nonresponders.”

Bar graphs are designed for categorical variables; yet they are commonly used to present continuous data in laboratory research, animal studies, and human studies with small sample sizes. Bar and line graphs of continuous data are “visual tables” that typically show the mean and standard error (SE) or standard deviation (SD). This is problematic for three reasons. First, many different data distributions can lead to the same bar or line graph (Fig 1 and Fig 2). The full data may suggest different conclusions from the summary statistics (Fig 1 and Fig 2). Second, additional problems arise when bar graphs are used to show paired or nonindependent data (Fig 2). Figures should ideally convey the design of the study. Bar graphs of paired data erroneously suggest that the groups being compared are independent and provide no information about whether changes are consistent across individuals (Panel A in Fig 2). Third, summarizing the data as mean and SE or SD often causes readers to wrongly infer that the data are normally distributed with no outliers. These statistics can distort data for small sample size studies, in which outliers are common and there is not enough data to assess the sample distribution.

In contrast, univariate scatterplots, box plots, and histograms allow readers to examine the data distribution. This approach enhances readers’ understanding of published data, while allowing readers to detect gross violations of any statistical assumptions. The increased flexibility of univariate scatterplots also allows authors to convey study design information. In small sample size studies, scatterplots can easily be modified to differentiate between datasets that include independent groups (Fig 1) and those that include paired or matched data (Fig 2).

We conducted a systematic review of standard practices for data presentation in scientific papers, contrasting the use of bar graphs versus figures that provide detailed information about the distribution of the data (scatterplots, box plots, and histograms). We focused on physiology because physiologists perform a wide range of studies, including human studies, animal studies, and in vitro laboratory experiments. We systematically reviewed all full-length, original research articles published in the top 25% of physiology journals between January 1 and March 31, 2014 (*n* = 703) to assess the types of figures that were used to present continuous outcome data (S1 Fig and Table A in S1 Text). We also abstracted information on sample size and statistical analysis procedures, as these factors may influence figure selection. Detailed methods and results are presented in the data supplement. Based on our findings, we recommend major changes to standard practices for presenting continuous data in small sample size studies. We hope that these recommendations will promote scientific discourse by giving readers the information needed to fully examine published data.

## Are Your Figures Worth a Thousand Words?

In addition to showing data for key findings, figures are important because they give authors the opportunity to display a large amount of data very quickly. However, most figures provided little more information than a table (Panel A in S2 Fig and S1 Text). Bar graphs were the most commonly used figures for presenting continuous data. 85.6% of papers included at least one bar graph. Most of these papers used bar graphs that showed mean ± SE (77.6%, Panel B in S2 Fig), rather than mean ± SD (15.3%). Line graphs and point and error bar plots were also common (61.3% of articles, Panel A in S2 Fig), and most showed mean ± SE. Figures that provide detailed information about the distribution of the data were seldom used. 13.4% of articles included at least one univariate scatterplot, 5.3% included at least one box plot, and 8.0% included at least one histogram. The journals that we examined publish research conducted by investigators in many fields; therefore, it is likely that investigators in other disciplines follow similar practices. The overuse of bar graphs and other figures that do not provide information about the distribution of the data has also been documented in psychology [3] and medicine [1,4].

**Fig 3:**
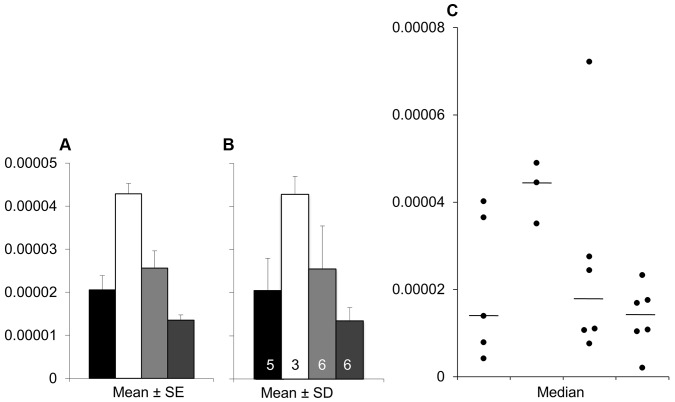
Bar graphs and scatterplots convey very different information. While scatterplots prompt the reader to critically evaluate the statistical tests and the authors’ interpretation of the data, bar graphs discourage the reader from thinking about these issues. Placental endothelin 1 (*EDN1*) mRNA data for four different groups of participants is presented in bar graphs showing mean ± SE (Panel A), or mean ± SD (Panel B), and in a univariate scatterplot (Panel C). Panel A (mean ± SE) suggests that the second group has higher values than the remaining groups; however, Panel B (mean ± SD) reveals that there is considerable overlap between groups. Showing SE rather than SD magnifies the apparent visual differences between groups, and this is exacerbated by the fact that SE obscures any effect of unequal sample size. The scatterplot (Panel C) clearly shows that the sample sizes are small, group one has a much larger variance than the other groups, and there is an outlier in group three. These problems are not apparent in the bar graphs shown in Panels A and B.

Our data show that most bar and line graphs present mean ± SE. Fig 3 illustrates that presenting the same data as mean ± SE, mean ± SD, or in a univariate scatterplot can leave the reader with very different impressions. While the scatterplot prompts the reader to critically evaluate the authors’ analysis and interpretation of the data, the bar graphs discourage the reader from thinking about these issues by masking distributional information. The question of whether investigators should report the SE or the SD has been extensively debated by biomedical scientists and statisticians [5,6]. We argue that figures for small sample size studies should show the full distribution of the data, rather than mean ± SE or mean ± SD. However, given that figures showing these summary statistics are ubiquitous in the biomedical literature, researchers should understand why the SE and SD can give such different visual impressions. The SD measures the variation in the sample, whereas the SE measures the accuracy of the mean. The SE is strongly dependent on sample size (SE = SD / √*n*)—as sample size increases, the uncertainty surrounding the value of the mean decreases. If two samples have the same SE, the one with the larger sample size will have the larger SD. Showing the SE rather than the SD magnifies the apparent visual differences between groups. This effect is exacerbated when the groups being compared have different sample sizes, which is common in physiology and in other disciplines.

The infrequent use of univariate scatterplots, boxplots, and histograms is a missed opportunity. The ability to independently evaluate the work of other scientists is a pillar of the scientific method. These figures facilitate this process by immediately conveying key information needed to understand the authors’ statistical analyses and interpretation of the data. This promotes critical thinking and discussion, enhances the readers’ understanding of the data, and makes the reader an active partner in the scientific process. In contrast, bar and line graphs are “visual tables” that transform the reader from an active participant into a passive consumer of statistical information. Without the opportunity for independent appraisal, the reader must rely on the authors’ statistical analyses and interpretation of the data.

## Summary Statistics Are Only Meaningful When There Are Enough Data to Summarize

Sample size is an important consideration when designing figures and selecting statistical analysis procedures (Box 1) for continuous data. Our analysis shows that most studies had very small sample sizes (Panel C in S2 Fig). The minimum sample size for any group shown in a figure was four (median number of independent observations), with an interquartile range of three independent observations (25th percentile: *n* = 3, 75th percentile: *n* = 6). The maximum sample size for any group shown in a figure was ten, with an interquartile range of nine (25th percentile: *n* = 6, 75th percentile: *n* = 15). Univariate scatterplots would be the best choice for many of these small studies. The summary statistics shown in bar graphs, line graphs, and box plots are only meaningful when there are enough data to summarize. Histograms are difficult to interpret when there aren’t enough observations to clearly show the distribution of the data.

**Box 1. Data Analysis**

- The distribution of the data and the sample size are critical considerations when selecting statistical tests. Univariate scatterplots immediately convey this important information.
- T-tests and analysis of variance (ANOVA) are examples of parametric tests. These tests compare means and assume that the data are normally distributed with no outliers. In small samples, these tests are prone to errors if the data contain outliers or are not normally distributed.
- The Wilcoxon rank sum test is an example of a nonparametric test. Nonparametric tests don’t make assumptions about the distribution of the variables that are being assessed. These tests often compare the ranks of the observations or the medians across groups. Nonparametric statistics are often preferred to parametric tests when the sample size is small and the data are skewed or contain outliers.
- Some statisticians recommend nonparametric tests for small sample size studies. Others argue that these tests are underpowered, especially if the data distribution appears symmetric.
- Our data suggest that most authors assume that their data are normally distributed, use parametric statistical analysis techniques, and select figures that show parametric summary statistics (Table B in S1 Text). 78.1% of studies performed only parametric analyses. 13.6% of studies used both parametric and nonparametric analyses, whereas 3.8% included only nonparametric analyses.
- More than half of the authors who performed non-parametric analyses showed means when presenting their data. Investigators should show medians whenever they use nonparametric statistical tests. Medians are often used in situations where the mean is misleading due to outliers or a skewed distribution.
- Investigators who use nonparametric statistics for paired or matched data should report the median difference instead of the median values for each condition (Fig 2). Unlike means, medians are not additive. The median difference is not the same as the difference between the medians for each condition.
- Scientists and statisticians continue to debate many statistical practices that are commonly used in basic science research. These include whether to test the assumptions underlying parametric analyses [7], when to use parametric versus nonparametric tests [8,9,10], whether to report SD versus SE for normally distributed data [5,6,8], and how to use *p*-values [11]. The data presentation practices that we recommend will benefit scientists and statisticians on all sides of these debates by allowing others to examine the potential impact of using different statistical techniques.

## Recommendations for a New Data Presentation Paradigm

These results suggest that, as scientists, we urgently need to change our standard practices for presenting and analyzing continuous data in small sample size studies. We recommend three changes to resolve the problems identified in this systematic review.

1. **Encourage a more complete presentation of data**. We encourage investigators to consider the characteristics of their datasets, rather than relying on standard practices in the field, whenever they present data. The best option for small datasets is to show the full data, as summary statistics are only meaningful if there are enough data to summarize. In 75% of the papers that we reviewed, the minimum sample size for any group shown in a figure was between two and six. Univariate scatterplots are the best choice for showing the distribution of the data in these small samples, as boxplots and histograms would be difficult to interpret. By displaying the full dataset, scatterplots allow readers to detect gross violations of statistical assumptions and to determine whether the results would have been different using alternative statistical analysis techniques. This is especially important for investigators who use parametric analyses to compare groups in small studies.
  While Microsoft Excel allows scientists to quickly and efficiently create bar graphs, univariate scatterplots are more challenging. We created free Excel templates that are available in the supplemental files for the manuscript (S2 Text and S3 Text). The templates can also be downloaded from CTSpedia (https://www.ctspedia.org/do/view/CTSpedia/TemplateTesting), where we will post updated versions. Researchers can quickly enter data to make univariate scatterplots for paired data, independent data, and independent data with points jittered so that points with similar values do not overlap. The supplemental files also include detailed instructions for investigators who wish to make univariate scatterplots for paired or independent data using Graph Pad PRISM (S4 Text, S5 Text and S6 Text).
2. **Change journal policies**. We strongly recommend that journals change their editorial policies and peer review practices to discourage the use of bar graphs and encourage the use of univariate scatterplots, boxplots, and histograms to present continuous data. Journal policies should provide specific guidance about what types of figures are preferred. Nonspecific policies stating that figures are preferred to tables whenever possible do not effectively promote the use of figures that show the distribution of continuous data (Table C in S1 Text, Table D in S1 Text, and S1 Text). Journals play a crucial role in redefining standard practices in scientific research [12]. However, editorial policies are only effective if they are implemented. There were few improvements in scientific reporting among animal studies two years after the Animal Research: Reporting of In Vivo Experiments (ARRIVE) guidelines were published, despite endorsement by top journals and funding agencies [13]. These guidelines were designed to encourage reporting of key methodological details in animal studies. Journals seeking to implement the policy changes recommended in this paper will need to work with editors and reviewers [14] to accomplish this goal.
3. **Train investigators in data presentation**. This systematic review demonstrates that scientists need better training in how to select the appropriate type of figure for their data. A visually appealing figure is of little value if it is not suitable for the type of data being presented. Investigators should consider the type of outcome variable (categorical versus continuous), the sample size and the study design (independent versus nonindependent data, etc.) when designing figures.
  Presenting data in scientific publications is a critical skill for scientists [15], although this information is not universally included in statistics courses. This systematic review demonstrates that most scientists who publish in top physiology journals work with very small datasets. However, in the authors’ experience, statistics courses in many basic science departments are taught by statisticians, epidemiologists, or other researchers who perform complex analyses in very large datasets. Effective statistics instruction cannot follow a “one size fits all” approach [15]. Statistics instructors need to consider the types of data that their students will be working with and the standard practices in their students’ fields when designing courses. Basic science departments should work with instructors to develop course materials that will address the needs of their students and faculty. Data presentation training should include techniques for small sample size studies and address the problems with the standard practices identified in this review.

## Conclusions

Our systematic review identified several critical problems with the presentation of continuous data in small sample size studies. A coordinated effort among investigators, medical journals, and statistics instructors is recommended to address these problems. We created free Excel templates (S2 Text and S3 Text, https://www.ctspedia.org/do/view/CTSpedia/TemplateTesting) that will allow researchers to quickly make univariate scatterplots for independent data (with or without overlapping points) and nonindependent data. We hope that improved data presentation practices will enhance authors’, reviewers’, and readers’ understanding of published data by ensuring that publications include the information needed to critically evaluate continuous data in small sample size studies.

## Supporting Information

S1 Text Supplemental methods and results. This file contains the methods and results for the systematic review, including Table A in S1 Text, Table B in S1 Text, Table C in S1 Text and Table D in S1 Text. Table A in S1 Text: The number of articles examined by journal. Values are n, or n (% of articles reviewed that were eligible and included in the analysis). Journals are organized by 2012 impact factor. Articles that were not full length original research articles were excluded after screening (i.e. reviews, editorials, perspectives, commentaries, letters to the editor, short communications, etc.). Abbreviations: AJP, American Journal of Physiology; APS, American Physiological Society. *APS Journal. Table B in S1 Text: Most studies performed parametric analyses. Values are n (%). *n (%) of 493 articles which performed parametric analyses. The remaining articles did not specifically state whether these assumptions were tested. Table C in S1 Text: Relationship between journal affiliation and the use of bar graphs and univariate scatterplots. Abbreviations: APS, American Physiological Society. Seven of the top 20 physiology journals are published by the American Physiological Society (APS), which specifies that outcome data should be presented in figures rather than in tables whenever possible. Nonhuman studies did not include human participants, tissues, cells or cell lines. Human studies included human participants, tissues, cells or cell lines. Table D in S1 Text: Relationship between journal affiliation and the use of histograms and line graphs/point and error bars plots. Abbreviations: APS, American Physiological Society. Seven of the top 20 physiology journals are published by the American Physiological Society (APS), which specifies that outcome data should be presented in figures rather than in tables whenever possible.
(DOCX)

S2 Text Excel templates for creating univariate scatterplots for independent data. Use this template to create scatterplots for independent data in two to five groups. Independent data means that the variable of interest is measured one time in each subject, and subjects are not related to each other. If your data do not meet these criteria, see the spreadsheet for paired or nonindependent data.
(XLSX)

S3 Text Excel templates for creating univariate scatterplots for paired or matched data. Use this template to create scatterplots for paired or matched data. Paired data are when you measure the variable of interest more than one time in each participant. Matched data are when participants in groups one and two are matched for important characteristics. If your data are independent, please see the template for independent data. The template will allow you to create scatterplots for one group with two conditions, or two groups with two conditions.
(XLS)

S4 Text Instructions for creating univariate scatterplots for independent data in GraphPad PRISM. Use these instructions to create univariate scatterplots for independent data in one or more groups of subject using GraphPad PRISM 6.0. Independent data means that the variable of interest is measured one time in each participant or specimen and participants or specimens are not related to each other. If your data are paired or matched, please see the instructions for paired or matched data.
(PDF)

S5 Text Instructions for creating univariate scatterplots for paired or matched data in GraphPad PRISM (one group, two conditions). Use these instructions to create univariate scatterplots for paired or matched data (two or more conditions) in one group of participants or specimens using GraphPad PRISM 6.0. Paired data are when you measure the variable of interest more than one time in each participant. Matched data are when participants in group one and group two are matched for important characteristics. If your data are independent, please see the instructions for independent data.
(PDF)

S6 Text Instructions for creating univariate scatterplots for paired or matched data in GraphPad PRISM (two groups, two conditions). Use these instructions to create scatterplots for paired data (two conditions) in two groups of participants or specimens using GraphPad PRISM 6.0. Paired data are when you measure the variable of interest more than one time in each participant. If your data are independent, please see the instructions for Independent data.
(PDF)

S1 Fig Study flow chart. (TIF)

S2 Fig Figure types, sample sizes, and statistical analysis. Panel a: Bar graphs and other figures that typically show mean and SE or mean and SD were strongly preferred to figures that provide detailed information about the distribution of the data (scatterplots, box plots, and histograms). Panel b: Most bar graphs show mean ± SE. Panel c: Box plots show the minimum and maximum sample sizes for any group presented in a figure. The box shows the median and interquartile range. Whiskers show the furthest point that is within 1.5 times the interquartile range. Note that a few very high outliers are not shown (*n* = 8 for minimum sample size; *n* = 7 for maximum sample size). The maximum values for minimum and maximum sample size per group were 593 and 2,192, respectively. Showing these outliers would make the box plots impossible to see. Seventeen studies were excluded from this analysis as sample size was not reported (*n* = 614). Panel d: The types of figures that are selected depend on the type of statistical analysis that is performed. We performed ordinal logistic regression, with analysis type and figure type both classified as ordinal variables. The distribution of figure types differed significantly between studies that performed only parametric analyses and studies that performed both parametric and nonparametric analyses (*p* < 0.001), and between studies that performed both types of analyses and studies that performed only nonparametric analyses (*p* < 0.001).
(TIF)

## Abbreviations

ANOVA: analysis of variance
ARRIVE: Animal Research: Reporting of In Vivo Experiments
SD: standard deviation
SE: standard error
