# Supplementary material for: Beyond Bar and Line Graphs: Time for a New Data Presentation Paradigm
Source: PLoS Biol. 2015 Apr 22;13(4):e1002128. doi: 10.1371/journal.pbio.1002128 (PMC4406565; doi:10.1371/journal.pbio.1002128)
Supplement: S1 Text — This file contains the methods and results for the systematic review, including Table A in S1 Text, Table B in S1 Text, Table C in S1 Text and Table D in S1 Text. Table A in S1 Text: The number of articles examined by journal. Values are n, or n (% of articles reviewed that were eligible and included in the analysis). Journals are organized by 2012 impact factor. Articles that were not full length original research articles were excluded after screening (i.e. reviews, editorials, perspectives, commentaries, letters to the editor, short communications, etc.). Abbreviations: AJP, American Journal of Physiology; APS, American Physiological Society. *APS Journal. Table B in S1 Text: Most studies performed parametric analyses. Values are n (%). *n (%) of 493 articles which performed parametric analyses. The remaining articles did not specifically state whether these assumptions were tested. Table C in S1 Text: Relationship between journal affiliation and the use of bar graphs and univariate scatterplots. Abbreviations: APS, American Physiological Society. Seven of the top 20 physiology journals are published by the American Physiological Society (APS), which specifies that outcome data should be presented in figures rather than in tables whenever possible. Nonhuman studies did not include human participants, tissues, cells or cell lines. Human studies included human participants, tissues, cells or cell lines. Table D in S1 Text: Relationship between journal affiliation and the use of histograms and line graphs/point and error bars plots. Abbreviations: APS, American Physiological Society. Seven of the top 20 physiology journals are published by the American Physiological Society (APS), which specifies that outcome data should be presented in figures rather than in tables whenever possible. (DOCX) [file pbio.1002128.s001.docx]

**Methods**

**Systematic Review of Literature:** We chose to examine studies published in highly ranked physiology journals for two reasons. First, physiologists perform a wide range of studies, including human studies, animal studies and in vitro laboratory experiments. Second, physiology journals provide a unique opportunity to examine the impact of journal policy. Seven of the top 20 physiology journals are published by the American Physiological Society (APS), which specifies that outcome data should be presented in figures rather than in tables whenever possible. This progressive policy should offer readers more insight into the distribution of the data. However, the policy will have little effect if most figures are bar or line graphs.

We examined the 20 top physiology journals, as determined by 2012 impact factors. Five journals that only publish review articles were excluded. Seven of the 15 remaining journals were affiliated with the APS, whereas eight journals were not affiliated with the APS. Two abstractors (TLW, NMM) reviewed all full length, original research articles published in issues of each journal between January 1 and March 31, 2014. Disagreements were resolved by consensus. Each article was screened. Papers were excluded if they did not include new data, did not have a continuous outcome variable, or only presented continuous variables in tables or in the text (Figure 4). Eligible manuscripts were reviewed in detail to evaluate the following six factors: 1.The types of figures that were used to display continuous data (bar graph, line graph, point and error bars plot, box plot, univariate scatter plot showing independent or paired data, histogram, Bland-Altman plot or correlation scatterplot); 2. The type of statistical analysis performed (parametric, non-parametric or both); 3. Whether the authors stated that they tested the normal distribution and equal variance assumptions; 4. The minimum and maximum sample sizes for any group shown in a bar graph, line graph, point and error bars plot, univariate scatterplot, or boxplot; 6. Whether bar graph legends stated what the bar height and error bars represented, and included the sample size of each group shown in the graph. Only the main paper was reviewed, as many readers do not examine online supplements. Figures showing representative values for a single subject or experiment were not included. This paper focuses on figures that compare continuous outcomes between groups; therefore papers that only included Bland-Altman plots or correlation scatterplots were excluded. Bland-Altman plots illustrate agreement between two different methods of measuring the same thing, whereas correlation scatterplots examine the relationship between two variables. In papers that provided information on both the number of independent units (animals or humans) or experiments (N) and the number of replicates (n), the number of independent units or experiments was recoded as the sample size. The total number of non-independent units or experiments (including replicates) was recorded as the sample size only when the number of independent units or experiments was not reported.

**Statistical Analysis:** Our objective was to assess the types of figures used to present data in physiology studies; therefore we examined all full length original research articles published in the top 25% of physiology journals for the first three months of 2014. Our primary outcome was the percentage of papers that included a bar graph of continuous data. We assumed that 50% of papers would contain bar graphs in order to quantify the maximum margin of error. Reviewing 600 papers would give a margin of error of less than 4% for a 95% confidence interval.

Categorical variables are presented as counts with percentages. The minimum and maximum sample sizes for any group shown in a figure were highly skewed, and are presented using box plots. Ordinal logistic regression was used to examine the effect of the relationship between the type of statistical analysis that was performed and the types of figures that were used. Logistic regression analysis was used to examine the effect of journal affiliation, study type and journal affiliation x study type interaction on the odds of including each type of figure. A separate model was created for each type of figure (bar graph, line graph/point and error bars plot, univariate scatterplot, box plot, histogram), and only significant predictors were included in the model. Stratified models were used to evaluate differences between subgroups when there was an interaction between study type and journal affiliation. Differences were considered statistically significant if p<0.05. Statistical analysis was performed using JMP (10.0.0, SAS Institute Inc., Cary, NC). Ethical approval was not required.

**Results**

943 articles were screened, and 703 articles were reviewed from 15 physiology journals (Figure 4, Table 1S). Seventy-two articles were excluded for the reasons outlined in Figure 4. Analyses were completed on the remaining 631 papers. Although the present paper argues that bar graphs are not the best method of presenting continuous data, most data here are presented in bar graphs. The irony of this point is not lost on the authors. However, bar graphs are designed to display categorical data and most outcomes in the present paper are categorical.

**Types of Figures:** Bar graphs were the most commonly used figure for presenting continuous data (85.6% of articles, Figure5a). Line graphs and point and error bar plots that typically show mean ± SE or SD were also common (61.3%, Figure 5a). Line graphs were more common than point and error bars plots (58.1% vs. 7.5%). Figures that provide detailed information about the distribution of the data were seldom used (Univariate scatterplots: 13.4%; Box plots: 5.3%; Histograms: 8.0%). Univariate scatterplots showing independent data were included in 9.6% of papers, whereas those showing non-independent or paired data were included in 4.2% of papers.

We then examined whether the types of figures selected depended on whether the article involved the use of human participants, tissues or cell lines. We also assessed the effects of journal policies that promote the use of figures. Seven of the top 20 physiology journals are published by the American Physiological Society (APS), which specifies that outcome data should be presented in figures rather than in tables whenever possible. The proportion of published studies that included univariate scatterplots and bar graphs depended on both study type (non-human vs. human) and journal policy (APS vs. non-APS journal). Publication in an APS journal was associated with higher rates of univariate scatterplot use in human studies (24.8% vs. 10.6%, Table 2S), but not in non-human studies (8.7% vs. 14.1%, p=0.002 for study type x journal policy). There was a trend towards a significant interaction between study type and journal policy for bar graph use (p=0.071). Publication in an APS journal was associated with higher rates of bar graph use among non-human studies (92.7% vs. 85.2%), but not among human studies (76.7% vs. 77.7%). The percentage of studies using box plots, histograms, or line graphs/point and error bars plots did not differ between human and non-human studies (data not shown); therefore, data were pooled. Compared to studies published in APS journals, histograms and line graphs or point and error bars plots were more common among studies published in non-APS journals (Table 3S). The proportion of papers that included a box plot did not differ between studies published in APS vs. non-APS journals.

Most bar graphs showed mean ± SE (Figure 5b). Among papers with at least one bar graph, bar height showed the group mean in 97.4% of papers. The error bars showed the SE in 77.6% of papers. The remaining papers used error bars to show the SD (15.3%), SE or SD (0.9%), other parameters (0.9%), did not state what the error bars represented (4.7%), or did not include error bars (0.6%). 48.3% of studies that included bar graphs always reported what values the bar height and error bars represented in the figure legend. 16.2% of papers included this information in some legends, whereas 35.4% never stated what values the bar height and error bars represented in the legend. Most line graphs (70.1%) and point and error bars plots (61.7%) also showed mean ± SE.

The SE shows the accuracy of the mean and depends on sample size. The SD shows the amount of variation in the sample, assuming that the data are normally distributed. One common argument for showing the SE is that the reader can calculate SD by multiplying the SE by √n. The information needed to calculate SD should ideally be included in the figure legend, as this allows readers to quickly estimate SD while they are examining the figure. We therefore examined the percentage of papers in which the legends for bar graphs showing mean and SE included all of the information required to calculate the SD from the SE (specifically, the sample size for each group and a statement that the bar graph showed mean and SE). Among 415 papers with mean ± SE bar graphs, only 21.7% (n = 90) included this information in legends for all bar graphs in the paper. 35.2% (n = 146) included this information in the legends of some bar graphs in the paper, and 43.1% (n = 179) did not include both pieces of information in any bar graph legend.

**Sample Size and Statistical Analyses for Continuous Variables:** The minimum and maximum sample sizes for any group shown in a figure (median, interquartile range) were 4 (interquartile range: 3, 6) and 10 (6, 15), respectively (Figure 5c). Despite these small sample sizes, most studies performed only parametric analyses (78.1%, Table 1). The remaining studies used both parametric and non-parametric analyses (13.6%), performed only non-parametric analyses (3.8%), did not state what type of analysis was performed (1.3%), or did not perform statistical analyses (3.2%). Among studies that performed only parametric analyses, 8.6% stated that they tested the normal distribution assumption. 4.5% stated that they tested the equal variance assumption. Minimum and maximum sample sizes were not different when the analysis was restricted to studies that performed only parametric analyses (data not shown).

The type of figures selected depended on the type of statistical analysis that was performed (Figure 5d; parametric vs. both parametric and non-parametric, p<0.001; both vs. non-parametric, p<0.001). The proportion of studies that included only bar graphs, line graphs and/or point and error bars plots was highest among studies that used only parametric analyses (80.8%), and decreased progressively in studies that used both types of statistics (62.4%) and studies that used only non-parametric statistics (41.7%). 25% of studies that performed only non-parametric analyses (n = 24) used only box plots, scatter plots and histograms, vs. 4.7% of papers that used both types of statistics and 1.2% of papers that only used parametric statistics. Among papers that performed any non-parametric analyses, 56% incorrectly presented data that were analyzed non-parametrically as mean ± SE or SD. Only 37.6% of papers appropriately presented data that were analyzed non-parametrically by showing box plots, scatterplots, or histograms, or stating the median and interquartile range or range in tables or in the text.

| **Table S1:** Number of articles examined by journal | | | |
| --- | --- | --- | --- |
| **Journal** | **Articles Screened**  (n = 943) | **Articles Reviewed**  (n = 703) | **Articles Included**  (n = 631) |
| Journal of Pineal Research | 22 | 21 | 21 (100%) |
| Pflugers Archives: European Journal of Physiology | 55 | 25 | 25 (100%) |
| Journal of General Physiology | 37 | 23 | 23 (100%) |
| AJP: Endocrinology and Metabolism* | 68 | 64 | 62 (97%) |
| Acta Physiologica | 65 | 39 | 37 (95%) |
| The Journal of Physiology | 121 | 71 | 67 (94%) |
| Chronobiology International | 34 | 25 | 18 (72%) |
| Journal of Cellular Physiology | 47 | 32 | 31 (97%) |
| AJP: Cell Physiology* | 70 | 47 | 47 (100%) |
| AJP: Gastrointestinal and Liver Physiology* | 55 | 49 | 46 (94%) |
| AJP: Heart and Circulatory Physiology* | 102 | 86 | 86 (100%) |
| AJP: Renal Physiology* | 79 | 62 | 55 (89%) |
| International Journal of Behavioral Nutrition and  Physical Activity | 45 | 40 | 5 (13%) |
| AJP: Lung, Cellular and Molecular Physiology* | 58 | 53 | 52 (98%) |
| Journal of Applied Physiology* | 85 | 66 | 56 (85%) |
| Values are n, or n (% of articles reviewed that were eligible and included in the analysis). Articles are organized by 2012 impact factor. Articles that were not full length original research articles were excluded after screening (i.e. reviews, editorials, perspectives, commentaries, letters to the editor, short communications, etc.).  Abbreviations: AJP, American Journal of Physiology; APS, American Physiological Society.  *APS Journal | | | |

| **Table S2:** Most Studies Performed Parametric Analyses | |
| --- | --- |
| **Statistical Analysis** | **All Articles**  (n = 631) |
| Parametric | 493 (78.1%) |
| Normal distribution assumption tested* | 43 (8.7%) |
| Equal variance assumption tested* | 22 (4.5%) |
| Parametric and non-parametric | 86 (13.6%) |
| Non-parametric | 24 (3.8%) |
| Not stated | 8 (1.3%) |
| No statistical analysis | 20 (3.2%) |
| Values are n (%).  *n (%) of 493 articles which performed parametric analyses. The remaining articles did not specifically state whether these assumptions were tested. | |

| **Table S3:** Effect of journal affiliation on the use of bar graphs and univariate scatterplots | | | | | | |
| --- | --- | --- | --- | --- | --- | --- |
| **Figure Type** | **Study Type** | **Non-APS Journal** | | **APS Journal** | | **p** |
|  |  | **n / total** | **%** | **n / total** | **%** |  |
| Bar graph | Non-human | 121/142 | 85.2% | 255/275 | 92.7% | 0.017* |
|  | Human | 66/85 | 77.7% | 99/129 | 76.7% | 0.878 |
| Univariate scatterplot | Non-human | 20/142 | 14.1% | 24/275 | 8.7% | 0.098 |
|  | Human | 9/85 | 10.6% | 32/129 | 24.8% | 0.008* |
| Abbreviations: APS, American Physiological Society.  Seven of the top 20 physiology journals are published by the American Physiological Society (APS), which specifies that outcome data should be presented in figures rather than in tables whenever possible. Non-human studies did not include human participants, tissues, cells or cell lines. Human studies included human participants, tissues, cells or cell lines. | | | | | | |

| **Table S4:** Effect of journal affiliation on use of histograms and line graphs/point and error bars plots | | | |
| --- | --- | --- | --- |
| **Figure Type** | **Non-APS Journal**  n (%) of 227 papers | **APS Journal**  n (%) of 404 papers | **p** |
| Box plot | 17 (7.5%) | 17 (4.2%) | 0.086 |
| Histogram | 27 (12.2%) | 23 (5.7%) | 0.007 |
| Line graph or point  and error bars plot | 152 (67.0%) | 236 (58.4%) | 0.033 |
| Abbreviations: APS, American Physiological Society.  Seven of the top 20 physiology journals are published by the American Physiological Society (APS), which specifies that outcome data should be presented in figures rather than in tables whenever possible. | | | |
