# Supplementary material for: Beyond Bar and Line Graphs: Time for a New Data Presentation Paradigm
Source: PLoS Biol. 2015 Apr 22;13(4):e1002128. doi: 10.1371/journal.pbio.1002128 (PMC4406565; doi:10.1371/journal.pbio.1002128)
Supplement: S4 Text — Use these instructions to create univariate scatterplots for independent data in one or more groups of subject using GraphPad PRISM 6.0. Independent data means that the variable of interest is measured one time in each participant or specimen and participants or specimens are not related to each other. If your data are paired or matched, please see the instructions for paired or matched data. (PDF) [file pbio.1002128.s004.pdf]

How to Create a Univariate Scatterplots for Independent Data (2 or more groups)
in GraphPad PRISM 6

These instructions will allow you to make graphs like this:

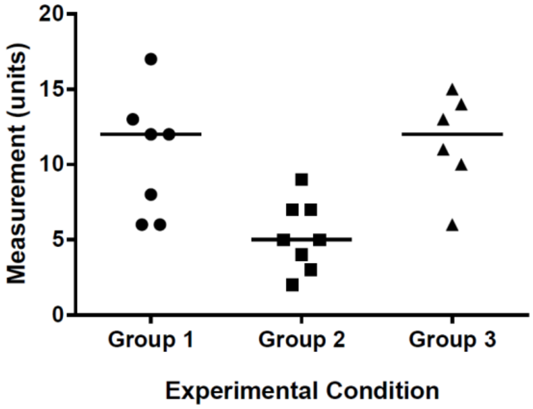

Use these instructions to create univariate scatterplots for independent data in two or more groups of subjects. Independent data means that the variable of interest is measured one time in each participant or specimens and participants or specimens are not related to each other. If your data are paired or matched, please see the instructions for paired or matched data.

Downloaded from: Weissgerber TL, Milic NM, Winham SJ, Garovic VD. Beyond Bar and Line Graphs: Time for a New Data Presentation Paradigm. *PLOS Biology*, 2015:13.

1. Under “New Table & Graph”, select “Column”
2. Select “Enter replicate values stacked into columns”. Click “Create”.

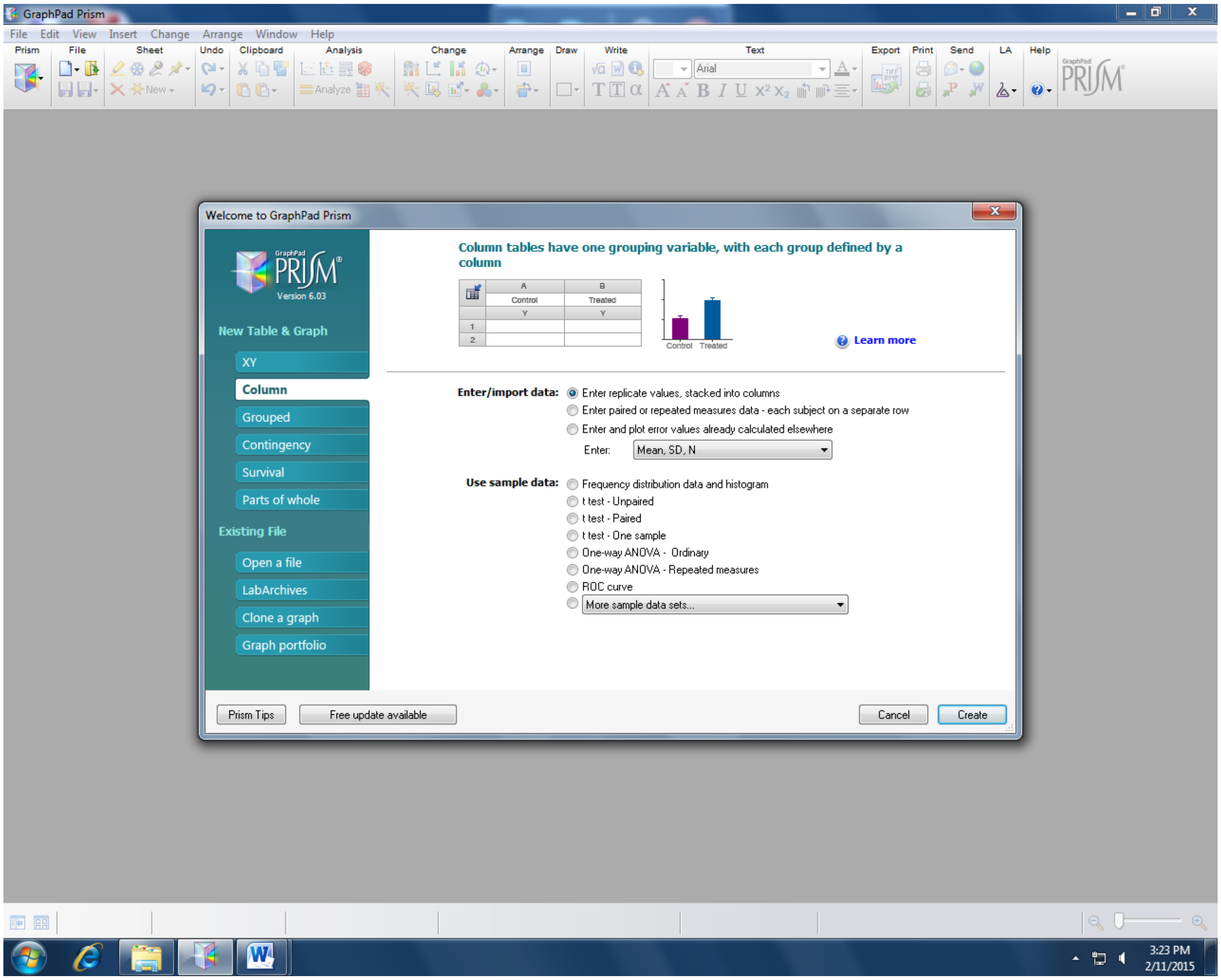

Downloaded from: Weissgerber TL, Milic NM, Winham SJ, Garovic VD. Beyond Bar and Line Graphs: Time for a New Data Presentation Paradigm. *PLOS Biology*, 2015:13.

3. Enter the group names under the column headings for “Group A”, “Group B”, “Group C”, etc. These will appear as your x-axis labels in your graph. Enter the data for each group, with 1 group per column.

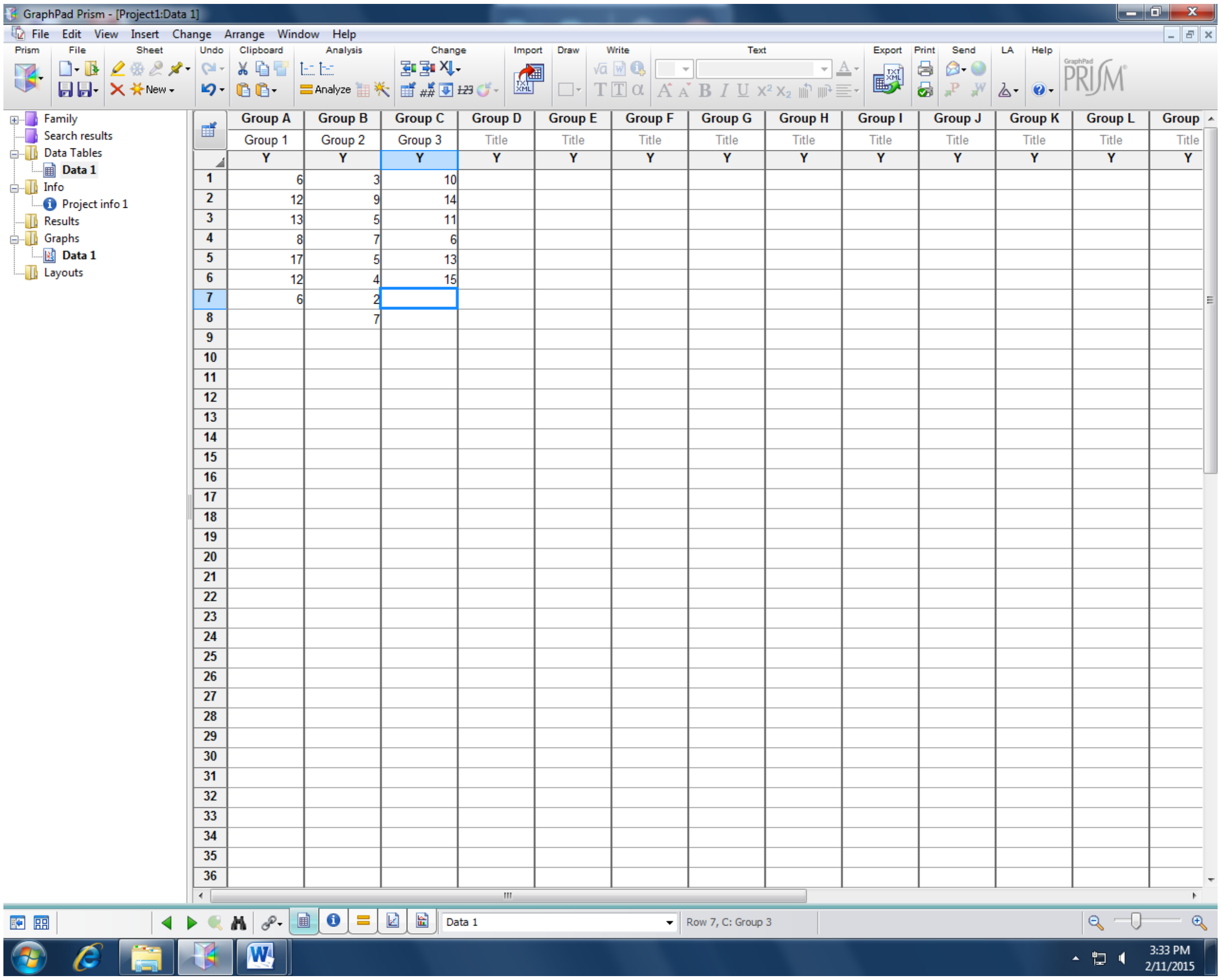

Downloaded from: Weissgerber TL, Milic NM, Winham SJ, Garovic VD. Beyond Bar and Line Graphs: Time for a New Data Presentation Paradigm. *PLOS Biology*, 2015:13.

4. From the insert menu at the top of the screen, select "New graph of existing data"

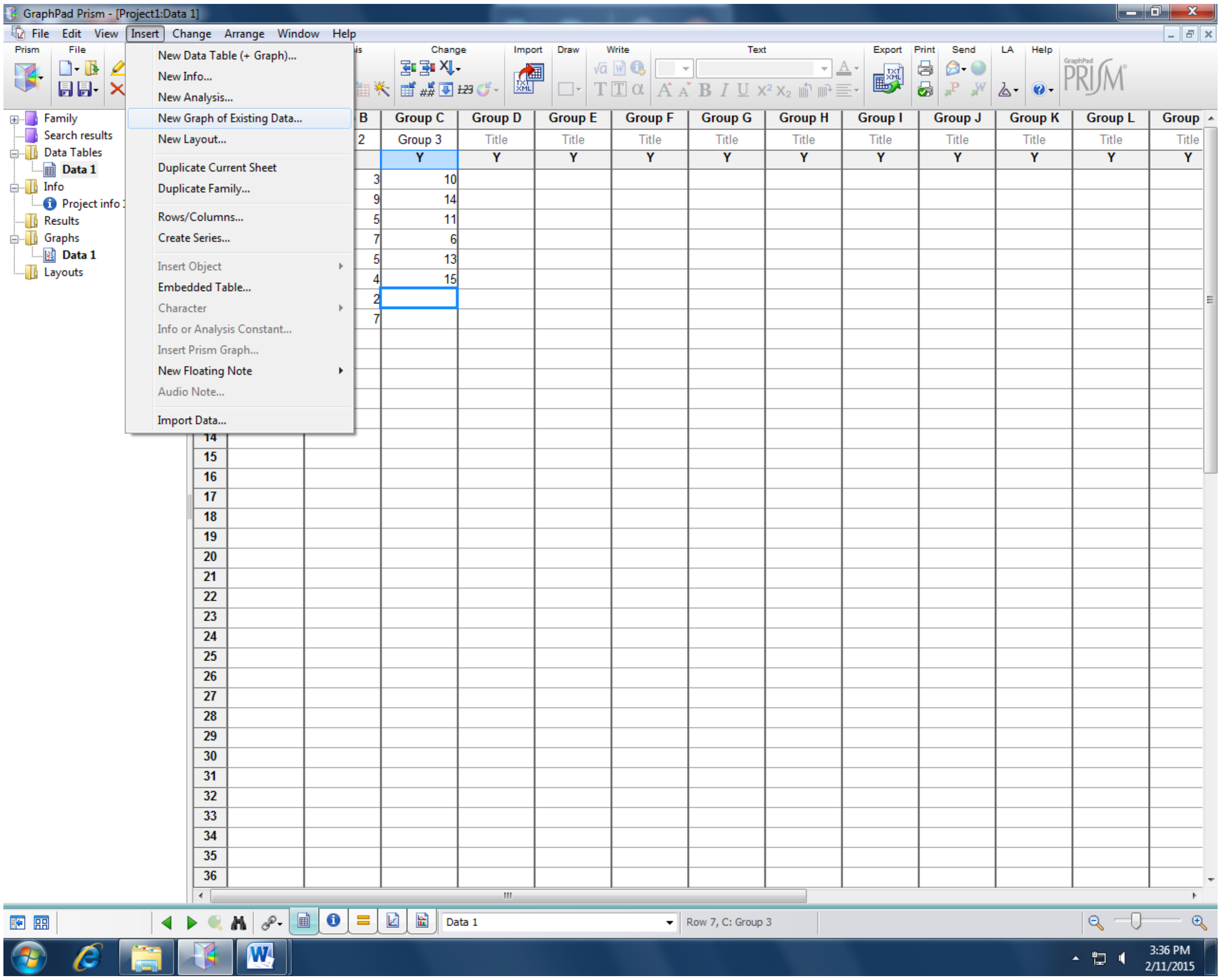

Downloaded from: Weissgerber TL, Milic NM, Winham SJ, Garovic VD. Beyond Bar and Line Graphs: Time for a New Data Presentation Paradigm. *PLOS Biology*, 2015:13.

5. Select “scatterplot” in the pop-up menu. Under the plot menu, select any summary statistics that you would like the graph to show. You should show the mean if you are using parametric statistics for your data analysis, and the median if you are using non-parametric statistics for your data analysis. Click “OK”.

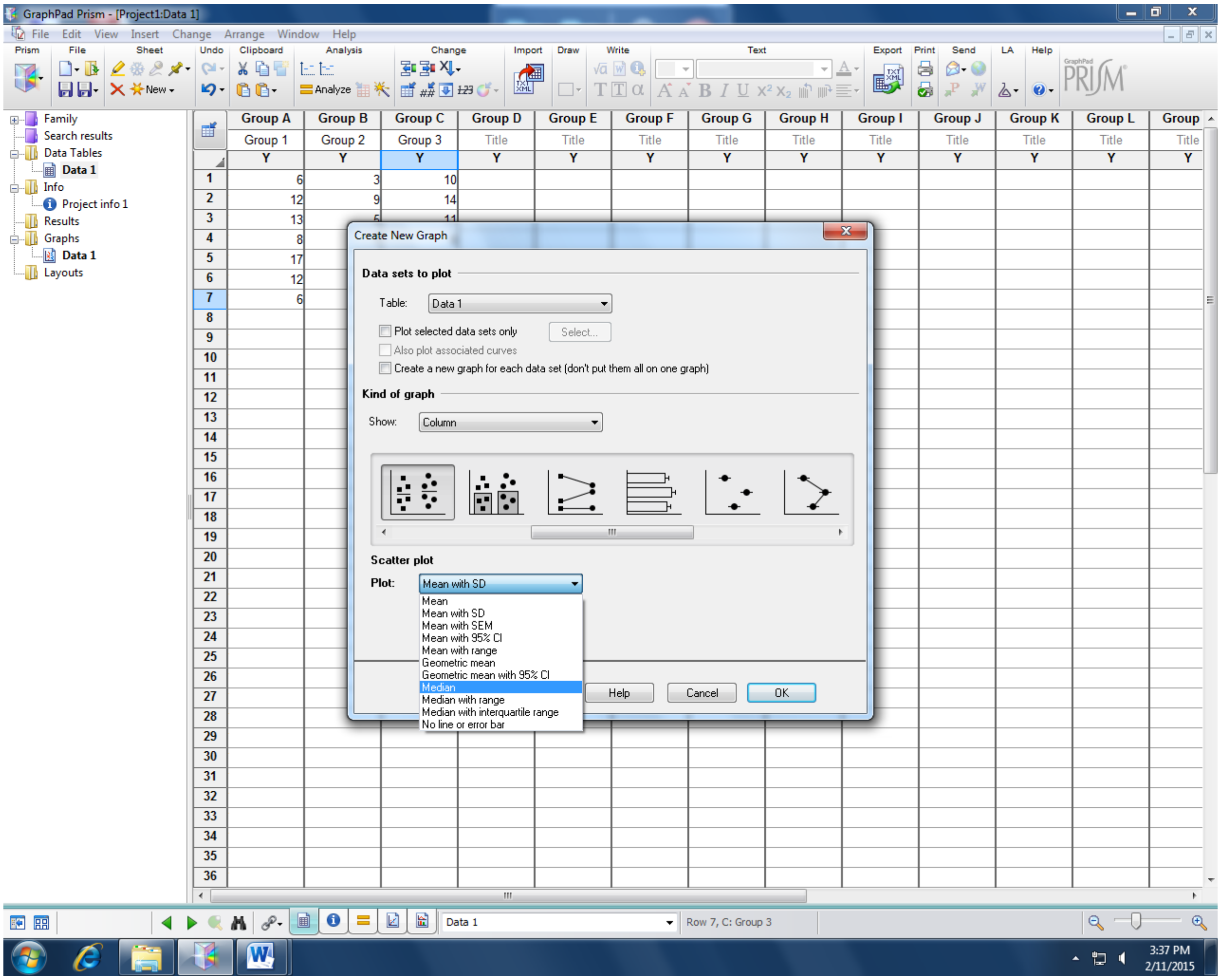

Downloaded from: Weissgerber TL, Milic NM, Winham SJ, Garovic VD. Beyond Bar and Line Graphs: Time for a New Data Presentation Paradigm. *PLOS Biology*, 2015:13.

6. The graph will look something like this. Click on “YTitle” to replace this label with the names and units for your axis. Either delete the chart title (Data1 in this example) and “XTitle” or click on these titles to replace them with the desired text.

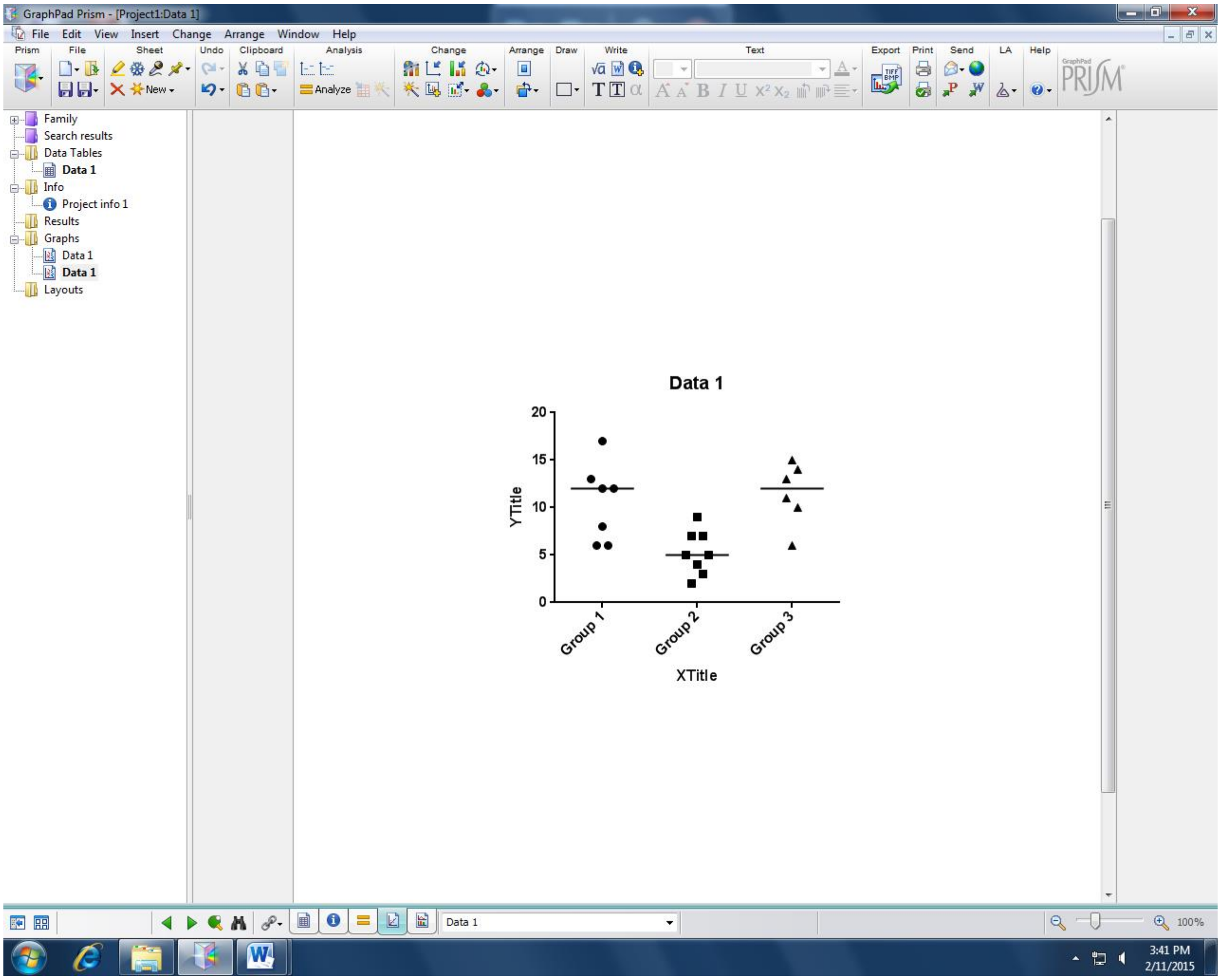

Downloaded from: Weissgerber TL, Milic NM, Winham SJ, Garovic VD. Beyond Bar and Line Graphs: Time for a New Data Presentation Paradigm. *PLOS Biology*, 2015:13.

7. Your new axis labels should now appear on the scatterplot.

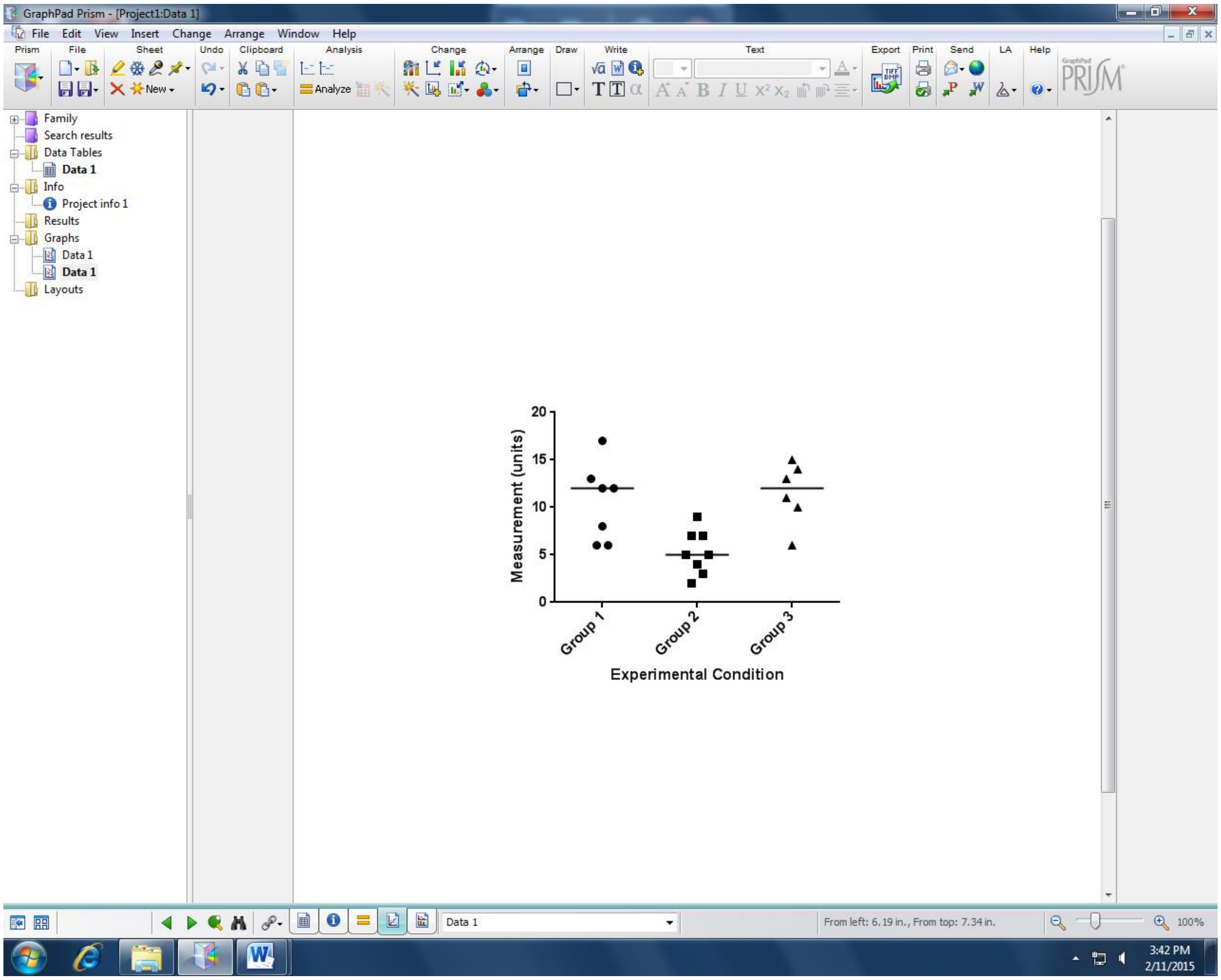

Downloaded from: Weissgerber TL, Milic NM, Winham SJ, Garovic VD. Beyond Bar and Line Graphs: Time for a New Data Presentation Paradigm. *PLOS Biology*, 2015:13.

8. Left click on one of the x-axis labels (Group 1, Group 2, etc.); then right click and select “Format axis”.

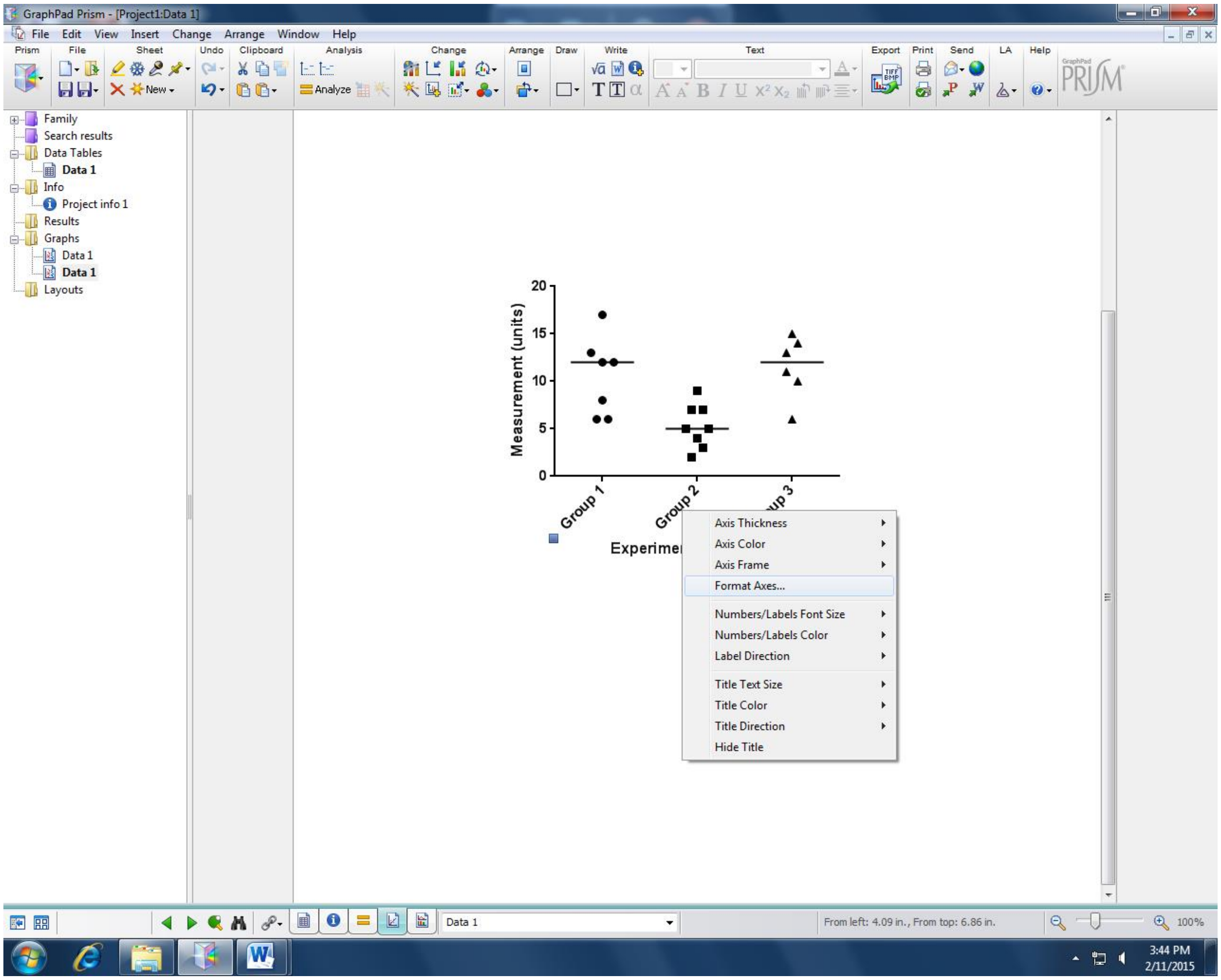

Downloaded from: Weissgerber TL, Milic NM, Winham SJ, Garovic VD. Beyond Bar and Line Graphs: Time for a New Data Presentation Paradigm. *PLOS Biology*, 2015:13.

9. In the pop-up menu, under the x-axis tab change “Below, angled” to “Below, horizontal”.

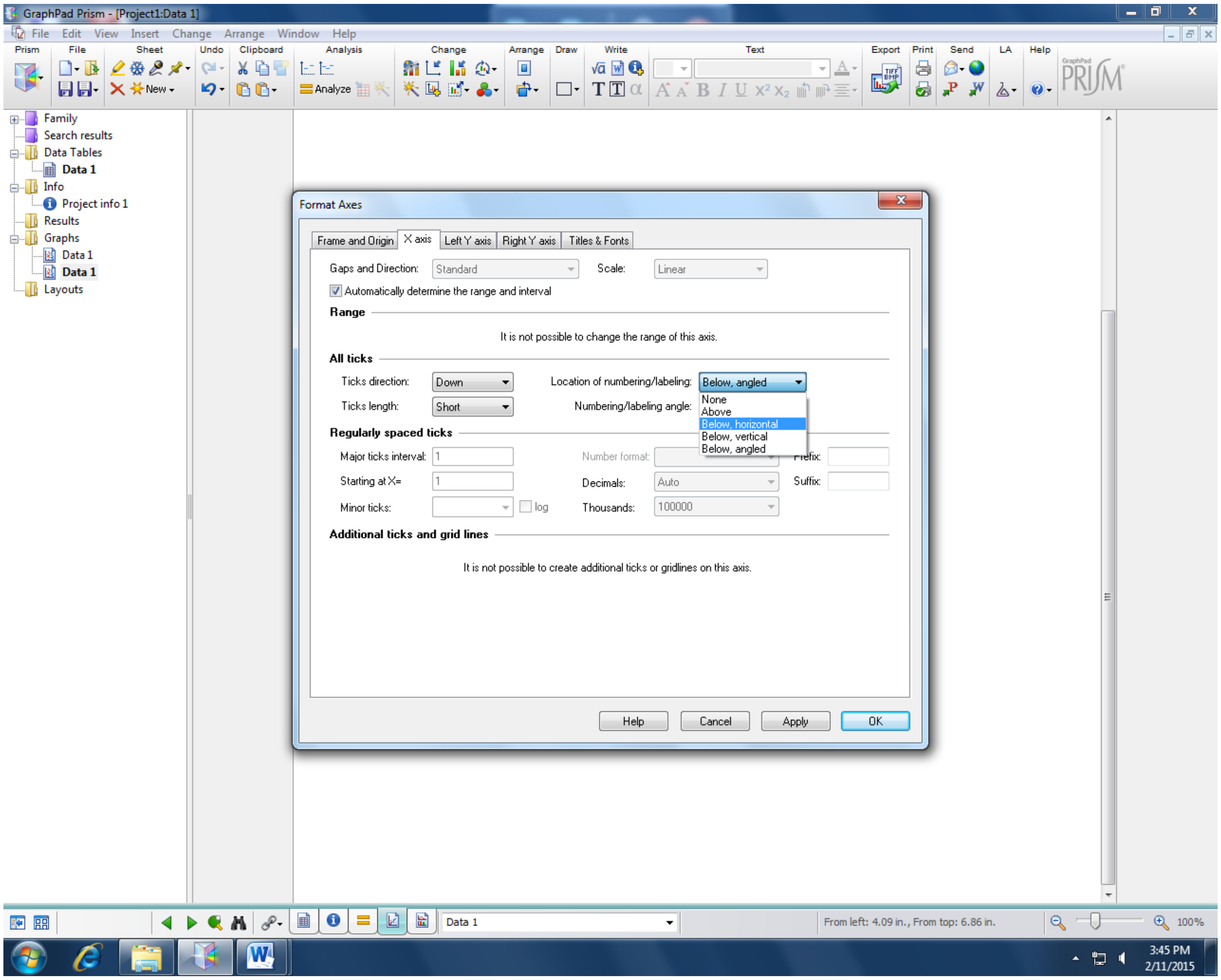

Downloaded from: Weissgerber TL, Milic NM, Winham SJ, Garovic VD. Beyond Bar and Line Graphs: Time for a New Data Presentation Paradigm. *PLOS Biology*, 2015:13.

10. If you are using an x-axis title, select the “Titles & Fonts” tab. Change ”Distance from axis” for the x-axis title to the desired value. Click “Apply” to view the change. Click “OK” when you are happy with the result.

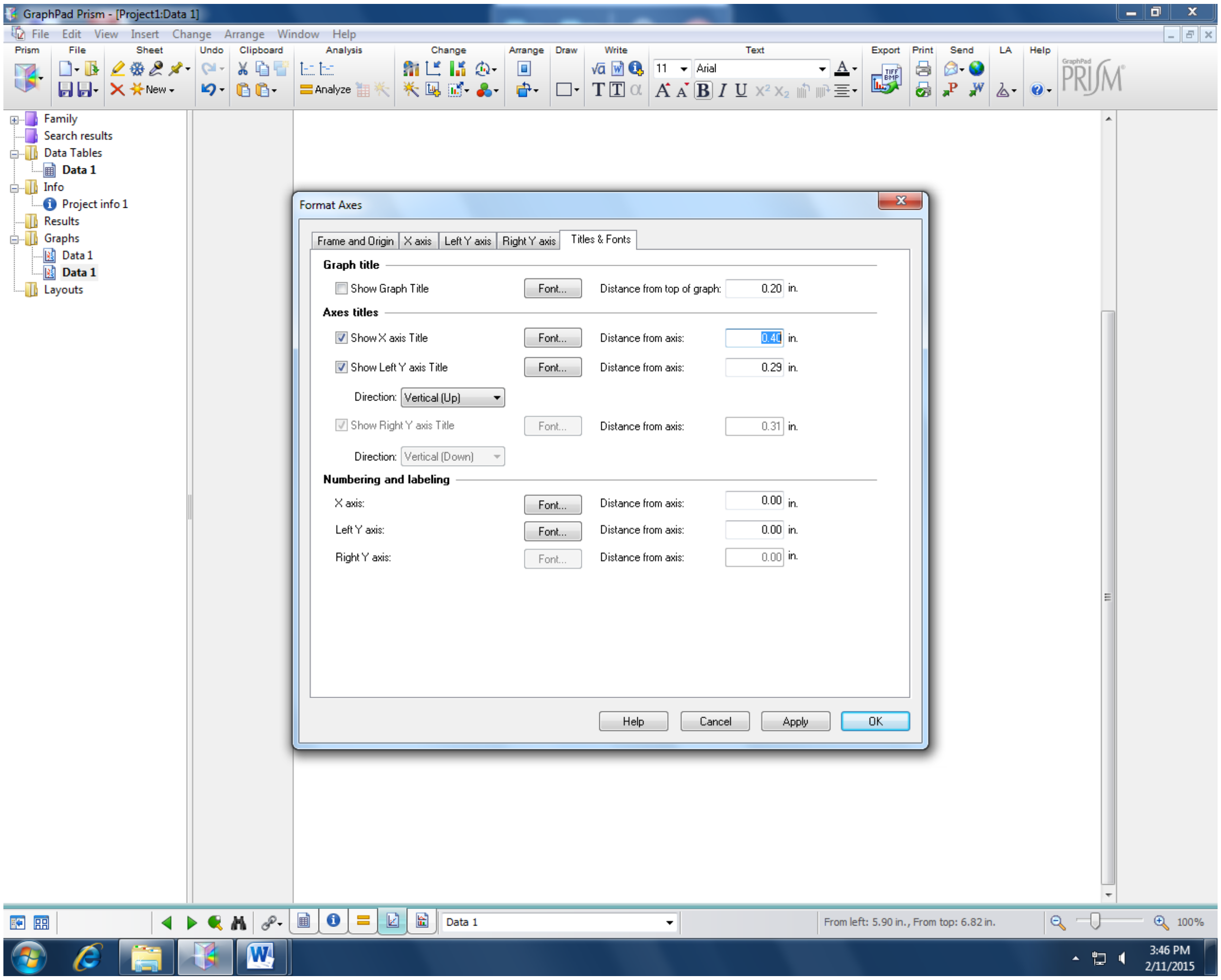

Downloaded from: Weissgerber TL, Milic NM, Winham SJ, Garovic VD. Beyond Bar and Line Graphs: Time for a New Data Presentation Paradigm. *PLOS Biology*, 2015:13.

11. Your univariate scatterplot will look something like this.

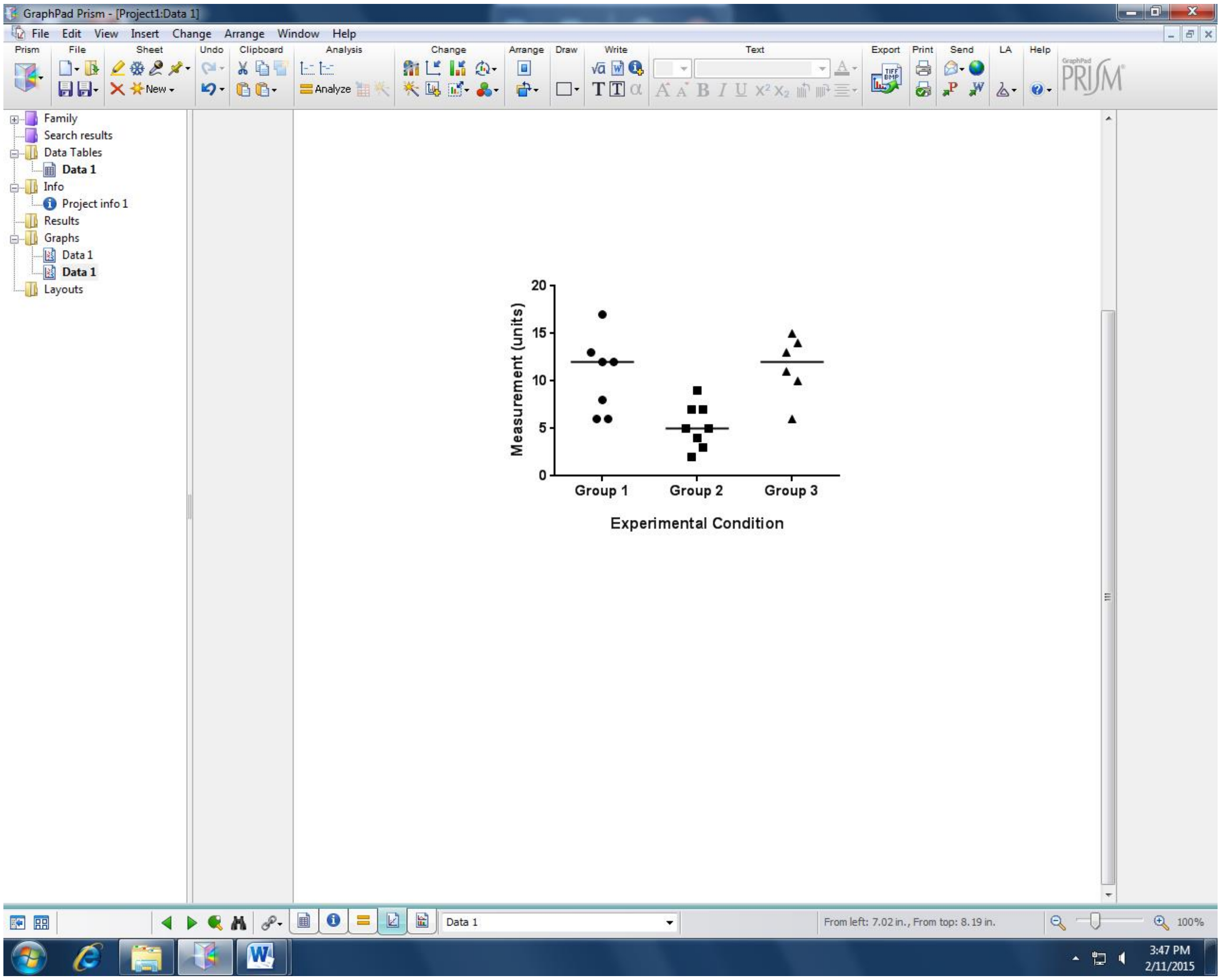

Downloaded from: Weissgerber TL, Milic NM, Winham SJ, Garovic VD. Beyond Bar and Line Graphs: Time for a New Data Presentation Paradigm. *PLOS Biology*, 2015:13.

12. Select “File”, then “Export” to save the scatterplot.

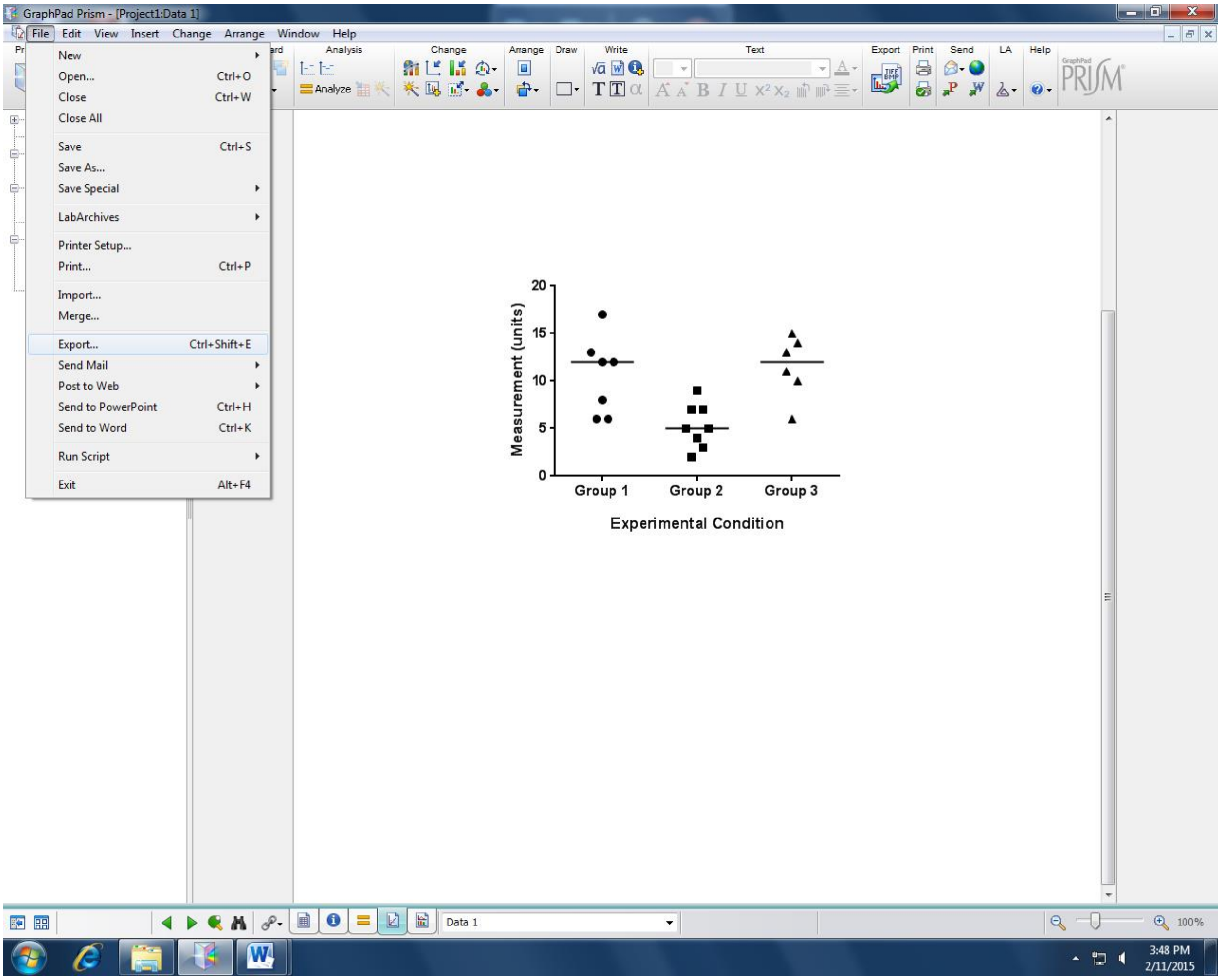

Downloaded from: Weissgerber TL, Milic NM, Winham SJ, Garovic VD. Beyond Bar and Line Graphs: Time for a New Data Presentation Paradigm. *PLOS Biology*, 2015:13.

13. Select the file format, enter a file name and chose the location where you want to save the graph. Click “OK”. Your scatterplot will be saved in the format and location that you selected.

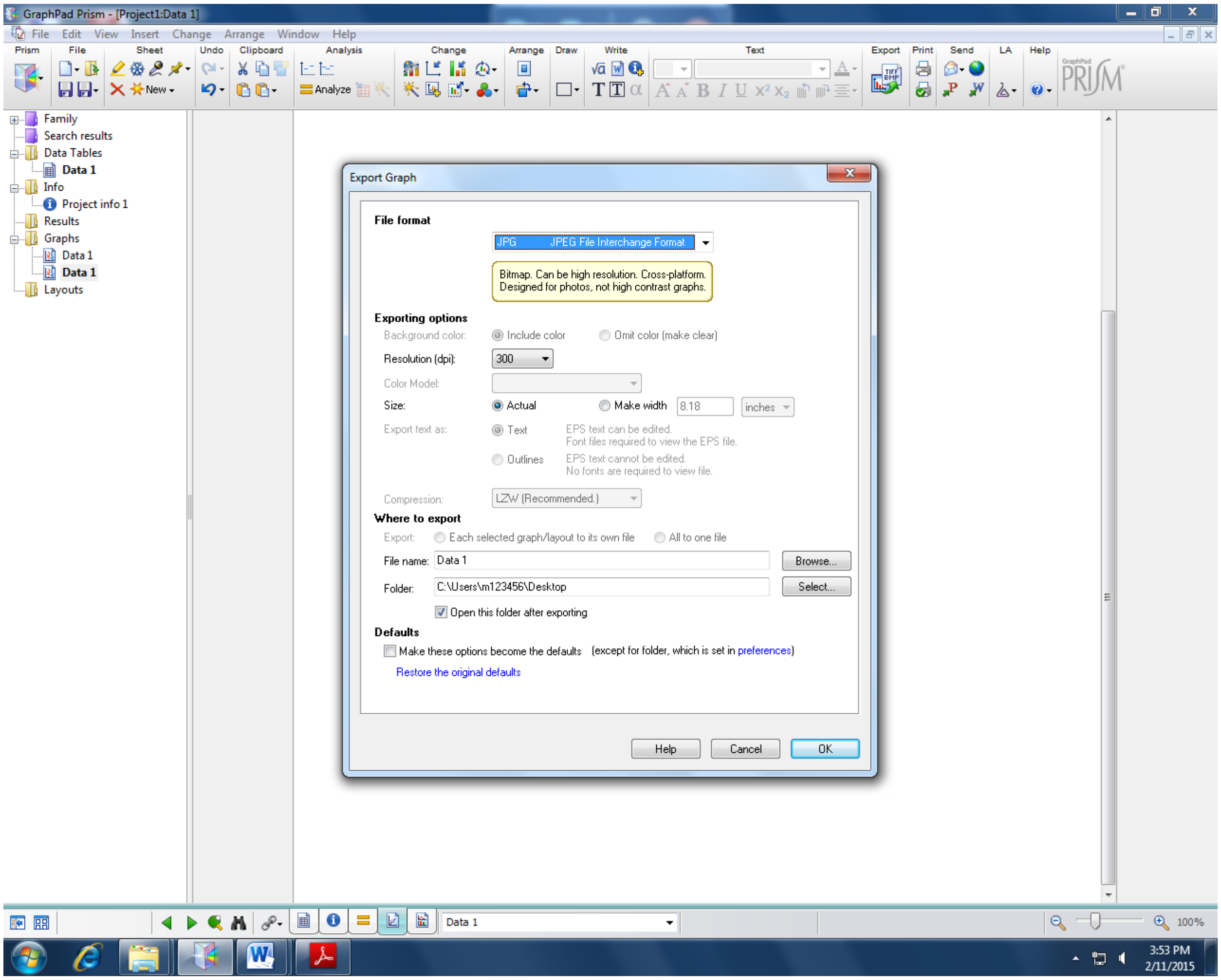

Downloaded from: Weissgerber TL, Milic NM, Winham SJ, Garovic VD. Beyond Bar and Line Graphs: Time for a New Data Presentation Paradigm. *PLOS Biology*, 2015:13.
