# Supplementary material for: Beyond Bar and Line Graphs: Time for a New Data Presentation Paradigm
Source: PLoS Biol. 2015 Apr 22;13(4):e1002128. doi: 10.1371/journal.pbio.1002128 (PMC4406565; doi:10.1371/journal.pbio.1002128)
Supplement: S5 Text — Use these instructions to create univariate scatterplots for paired or matched data (two or more conditions) in one group of participants or specimens using GraphPad PRISM 6.0. Paired data are when you measure the variable of interest more than one time in each participant. Matched data are when participants in group one and group two are matched for important characteristics. If your data are independent, please see the instructions for independent data. (PDF) [file pbio.1002128.s005.pdf]

How to Create a Univariate Scatterplots for Paired or Matched Data
(1 Group, 2+ conditions) in GraphPad PRISM 6

These instructions will allow you to make graphs like this:

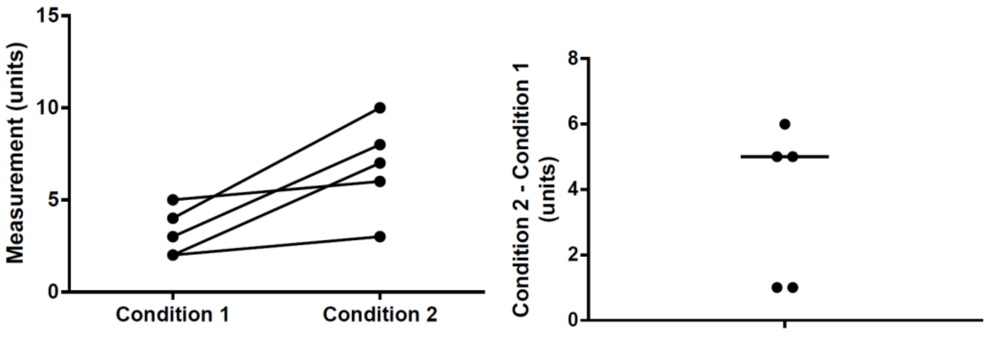

Use these instructions to create scatterplots for paired or matched data (2 or more conditions) in one group of subjects. Paired data are when you measure the variable of interest more than one time in each participant. Matched data are when participants in group 1 and group 2 are matched for important characteristics. If your data are independent, please see the instructions for Independent data.

Downloaded from: Weissgerber TL, Milic NM, Winham SJ, Garovic VD. Beyond Bar and Line Graphs: Time for a New Data Presentation Paradigm. *PLOS Biology*, 2015:13.

1. Under “New Table & Graph”, select “Column”
2. Select “Enter paired or repeated measures data – each subject on a separate row”. Click “Create”.

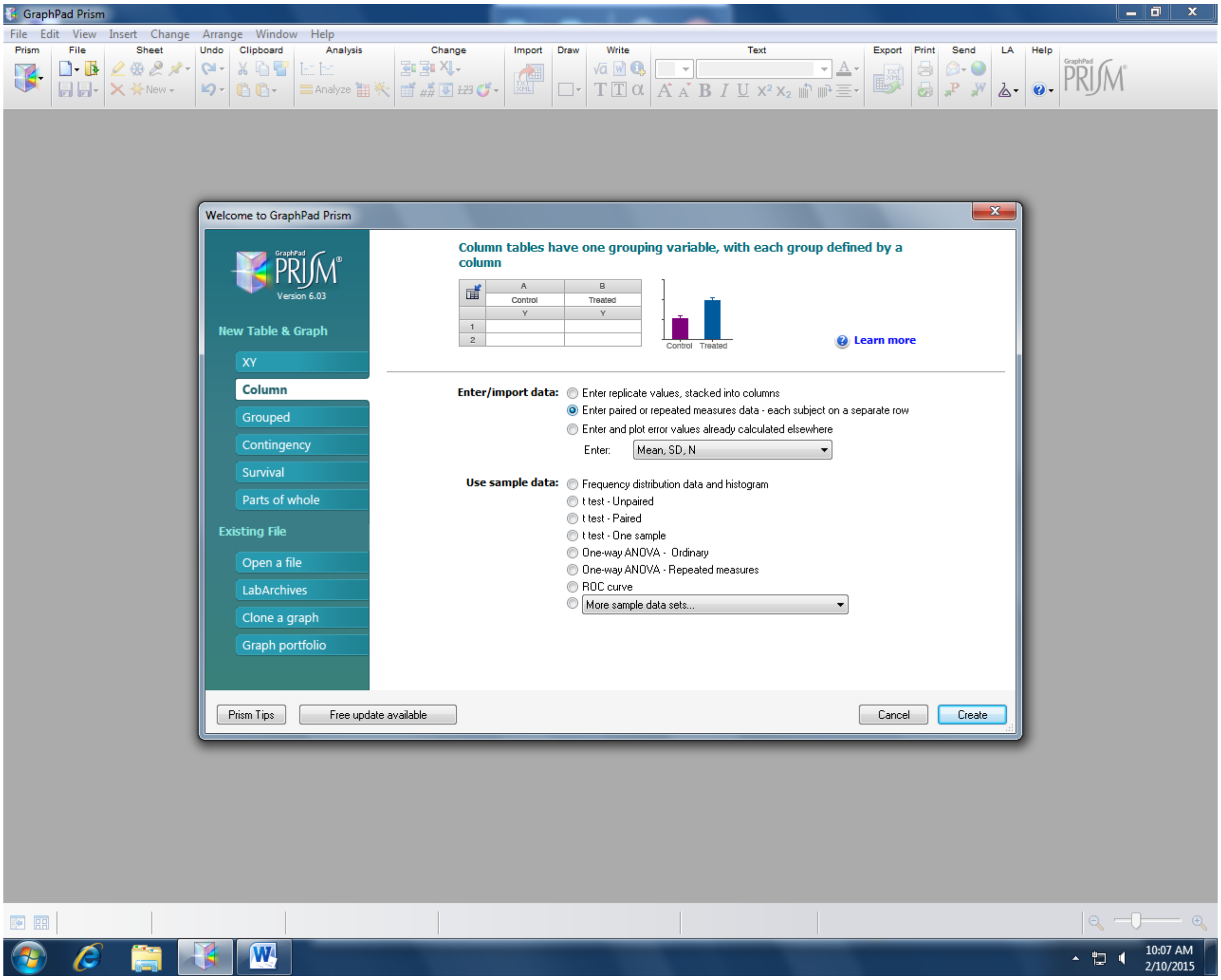

Downloaded from: Weissgerber TL, Milic NM, Winham SJ, Garovic VD. Beyond Bar and Line Graphs: Time for a New Data Presentation Paradigm. *PLOS Biology*, 2015:13.

3. Enter the name for Condition 1 just below Group A. Enter the name for Condition 2 just below Group B. The condition names that you enter will appear as labels on your graph. Enter the subject identifiers in the Column before the Group A column. Enter data for Condition 1 in the Group A column and Condition 2 in the Group B column, with one row per subject.

   This example uses 2 conditions. If you have more conditions, change the labels and enter the data in additional columns as needed.

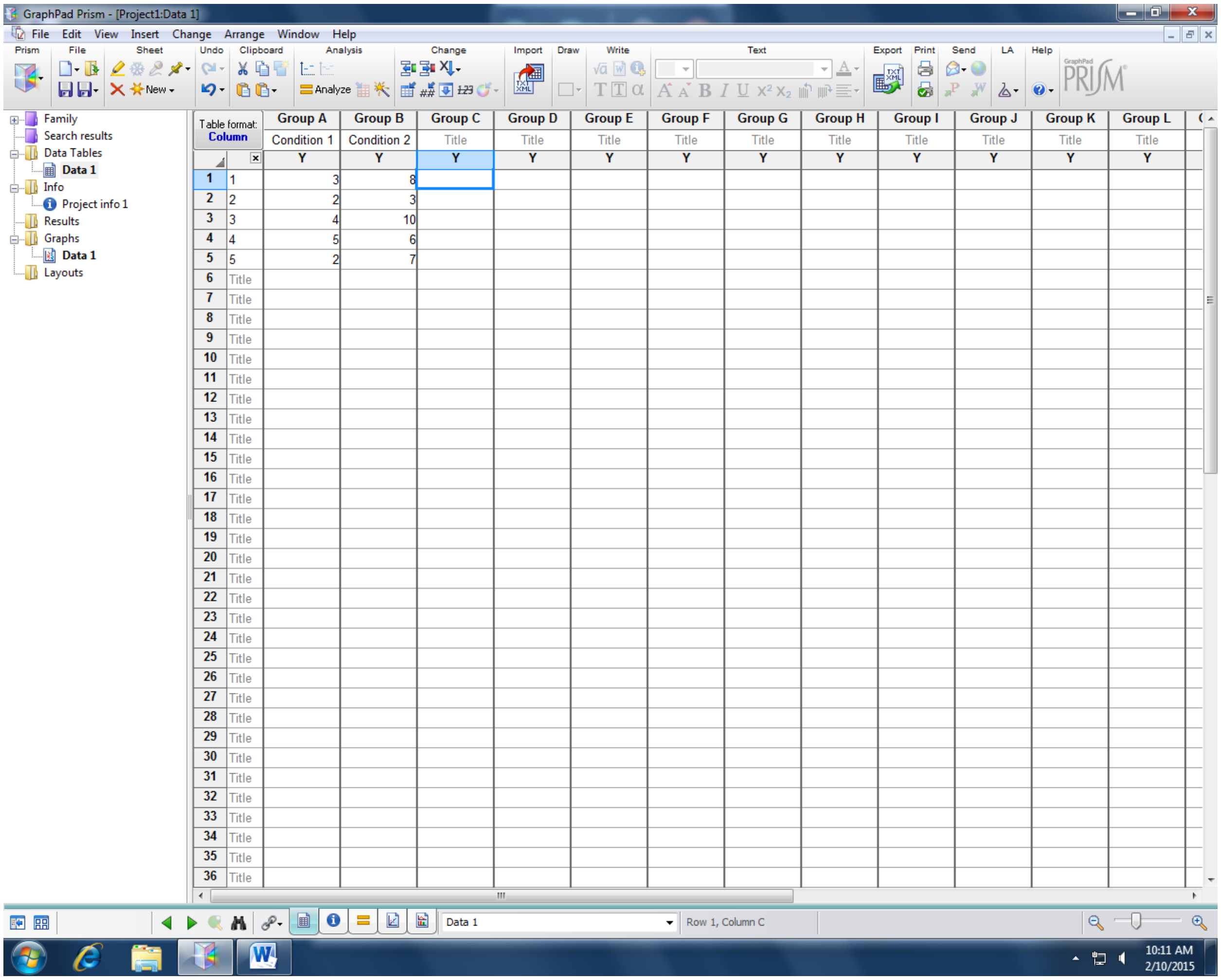

Downloaded from: Weissgerber TL, Milic NM, Winham SJ, Garovic VD. Beyond Bar and Line Graphs: Time for a New Data Presentation Paradigm. *PLOS Biology*, 2015:13.

4. From the insert menu at the top of the screen, select “New graph of existing data”

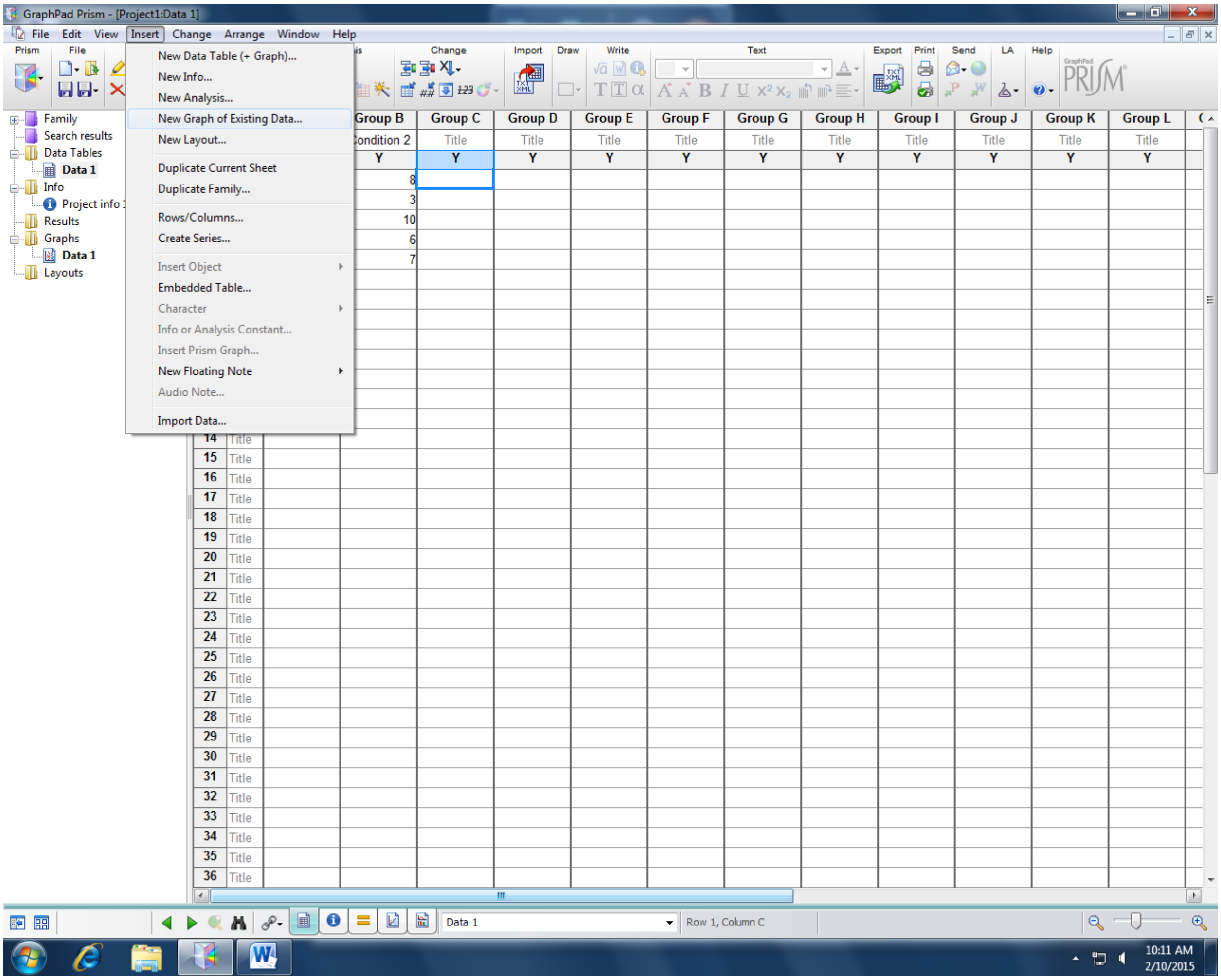

Downloaded from: Weissgerber TL, Milic NM, Winham SJ, Garovic VD. Beyond Bar and Line Graphs: Time for a New Data Presentation Paradigm. *PLOS Biology*, 2015:13.

5. Ensure that the “Symbols & lines” plot is selected. Click “OK” to create the graph.

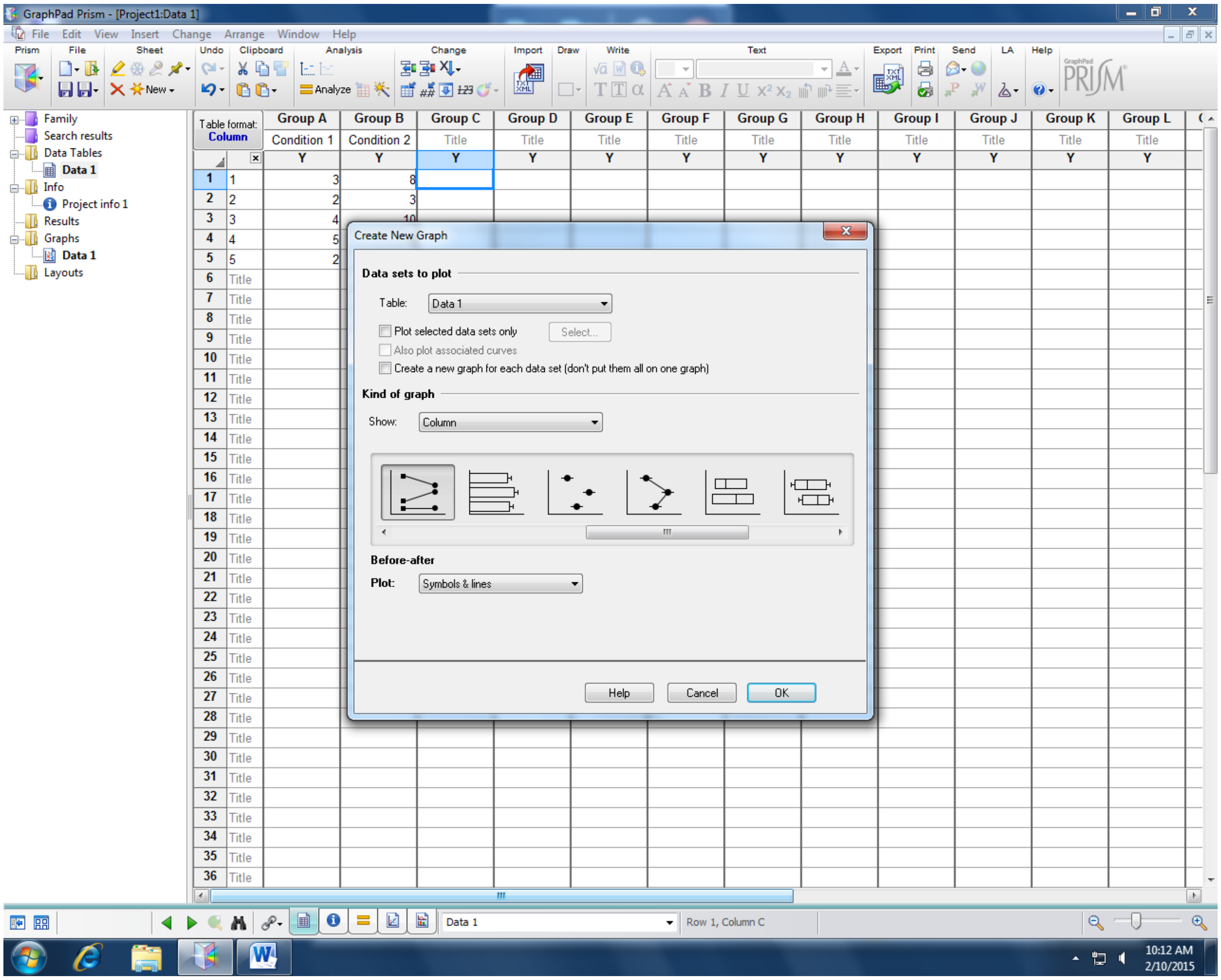

Downloaded from: Weissgerber TL, Milic NM, Winham SJ, Garovic VD. Beyond Bar and Line Graphs: Time for a New Data Presentation Paradigm. *PLOS Biology*, 2015:13.

6. The graph will look something like this. Click on “YTitle” to replace this label with the names and units for your axis. Either delete the chart title (Data1 in this example) and “XTitle” or click on these titles to replace them with the desired text.

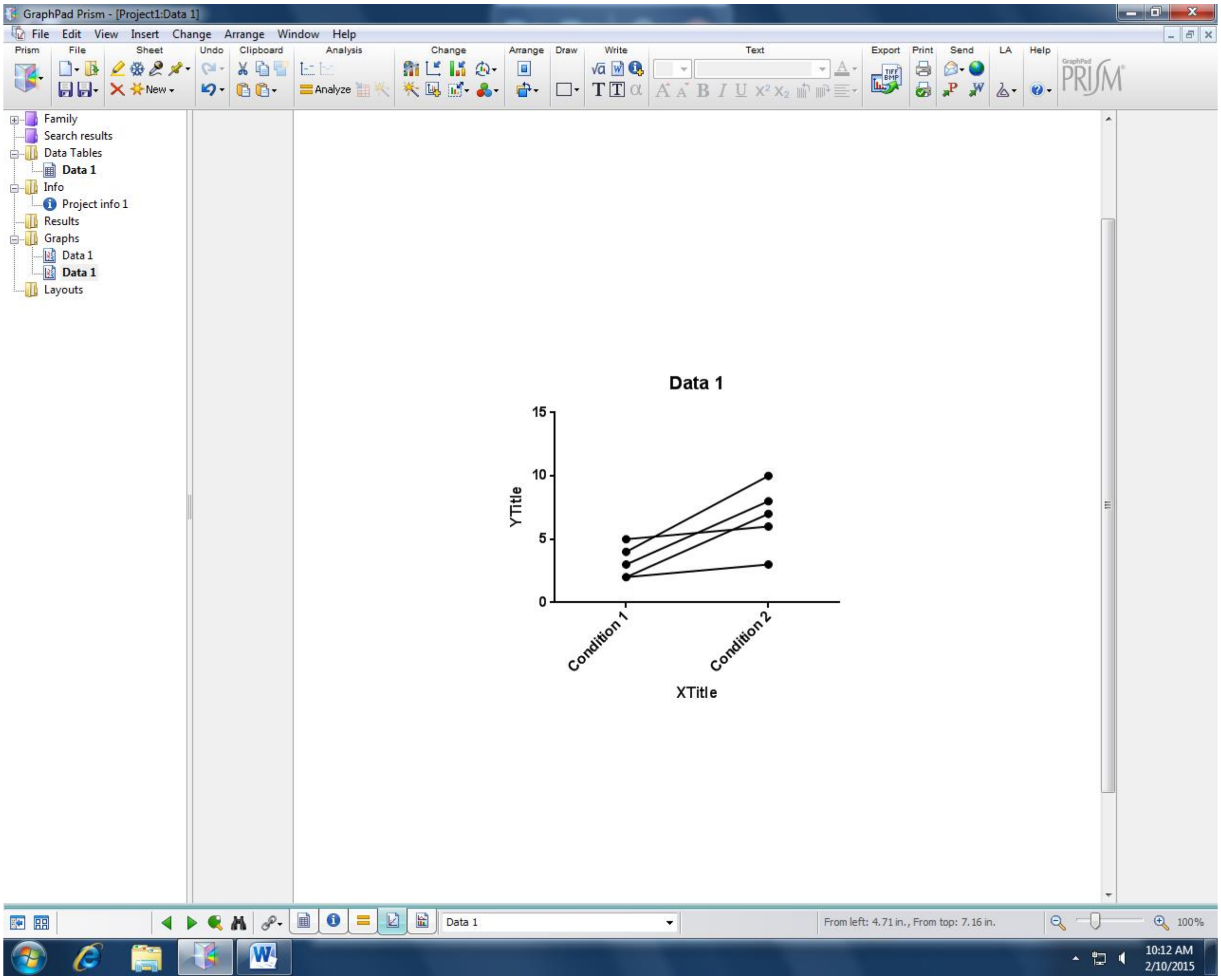

Downloaded from: Weissgerber TL, Milic NM, Winham SJ, Garovic VD. Beyond Bar and Line Graphs: Time for a New Data Presentation Paradigm. *PLOS Biology*, 2015:13.

7. Your new axis labels should now appear on the scatterplot.

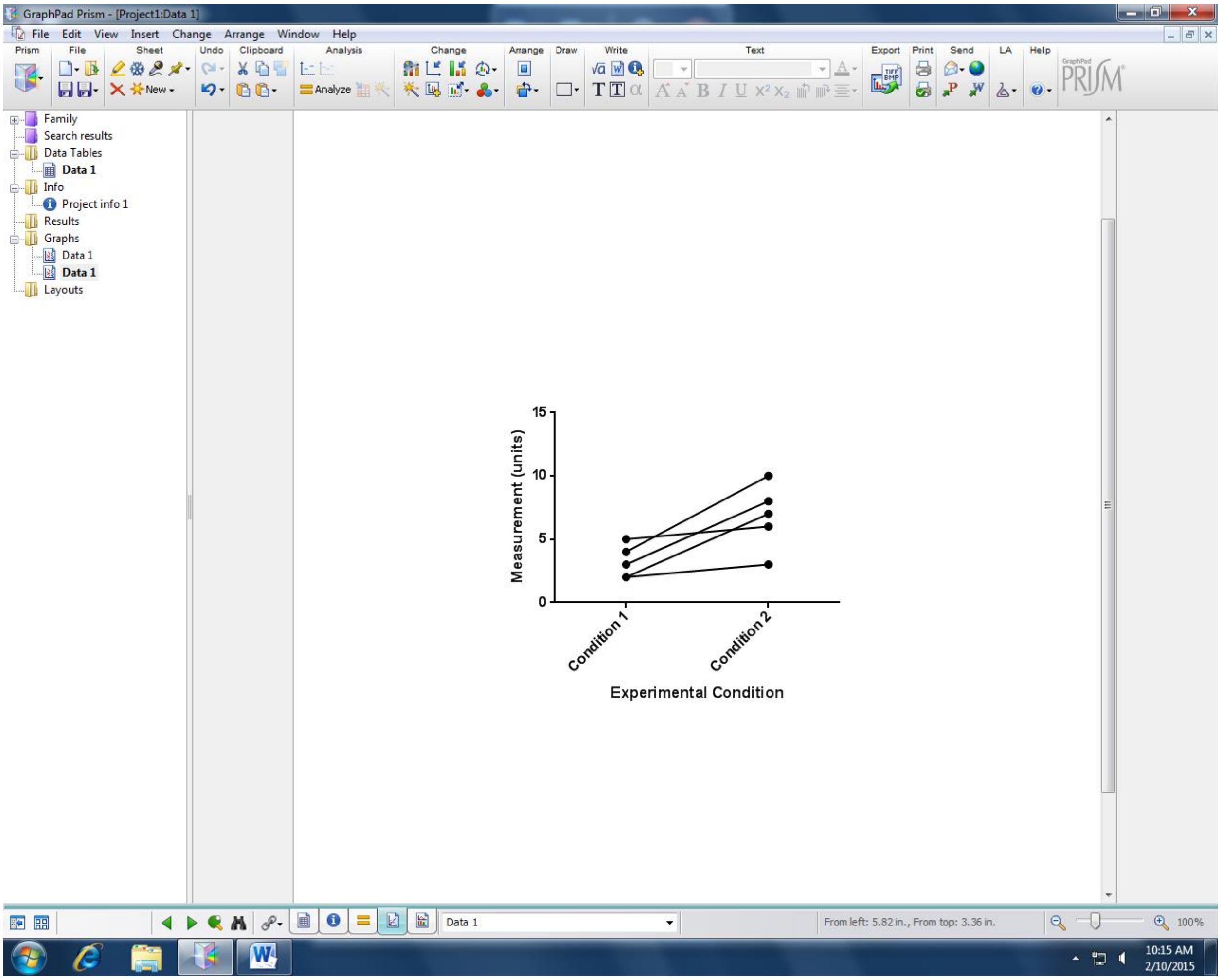

Downloaded from: Weissgerber TL, Milic NM, Winham SJ, Garovic VD. Beyond Bar and Line Graphs: Time for a New Data Presentation Paradigm. *PLOS Biology*, 2015:13.

8. Left click on one of the x-axis labels (Condition 1 or Condition 2); then right click and select “Format axis”.

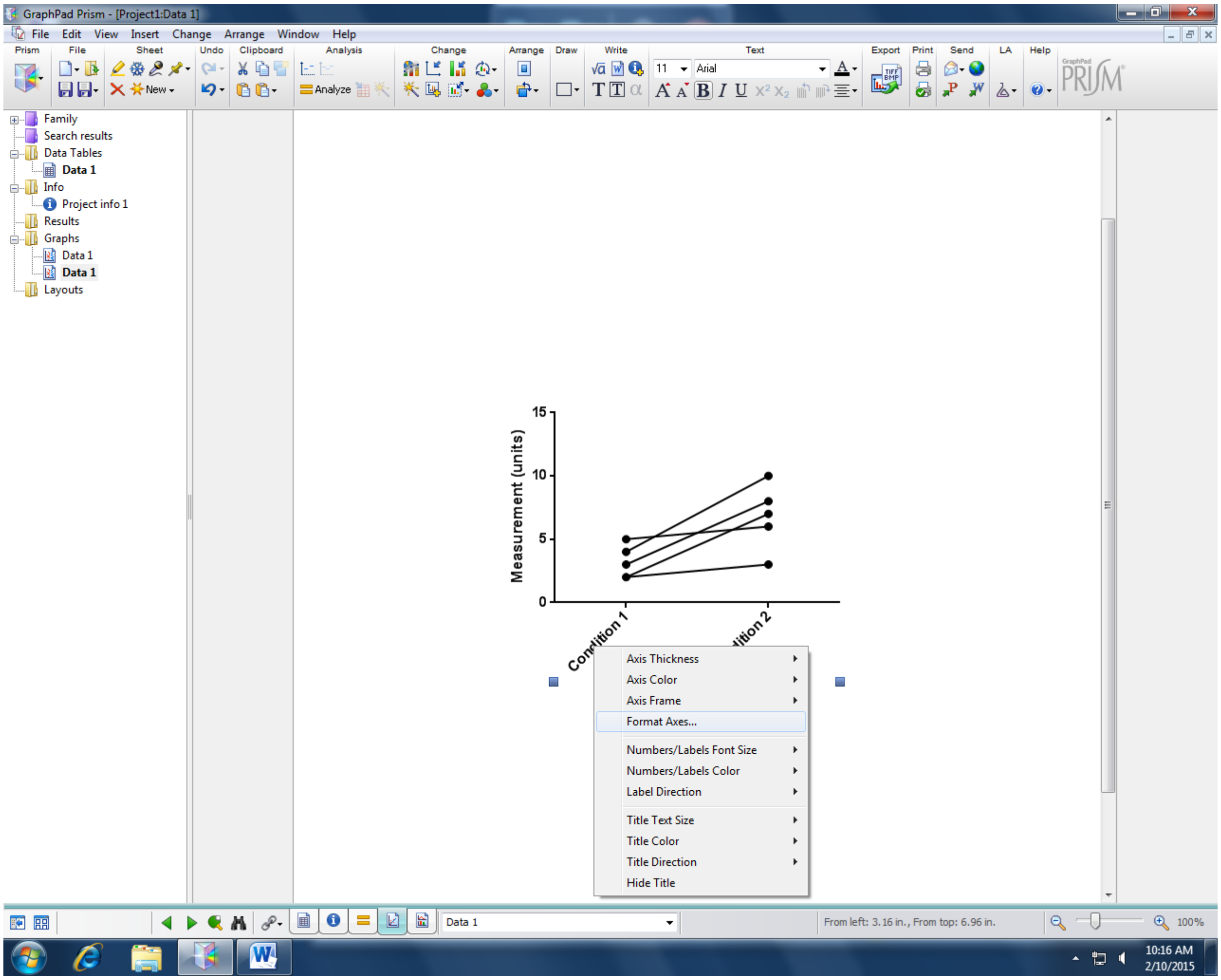

Downloaded from: Weissgerber TL, Milic NM, Winham SJ, Garovic VD. Beyond Bar and Line Graphs: Time for a New Data Presentation Paradigm. *PLOS Biology*, 2015:13.

9. In the pop-up menu, under the x-axis tab change “Below, angled” to “Below, horizontal”.

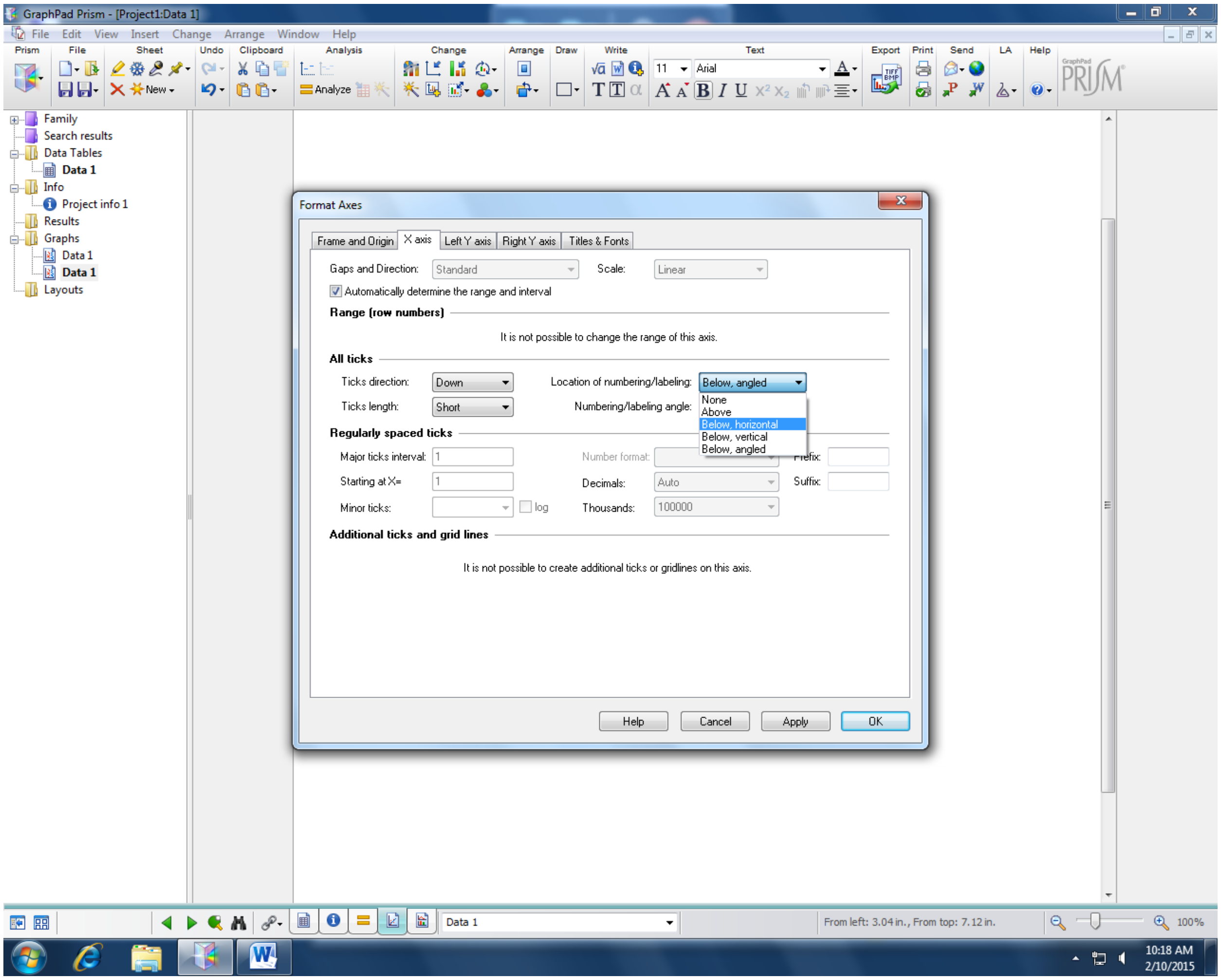

Downloaded from: Weissgerber TL, Milic NM, Winham SJ, Garovic VD. Beyond Bar and Line Graphs: Time for a New Data Presentation Paradigm. *PLOS Biology*, 2015:13.

10. If you are using an x-axis title, select the “Titles & Fonts” tab. Change ”Distance from axis” for the x-axis title to the desired value. Click “Apply” to view the change. Click “OK” when you are happy with the result.

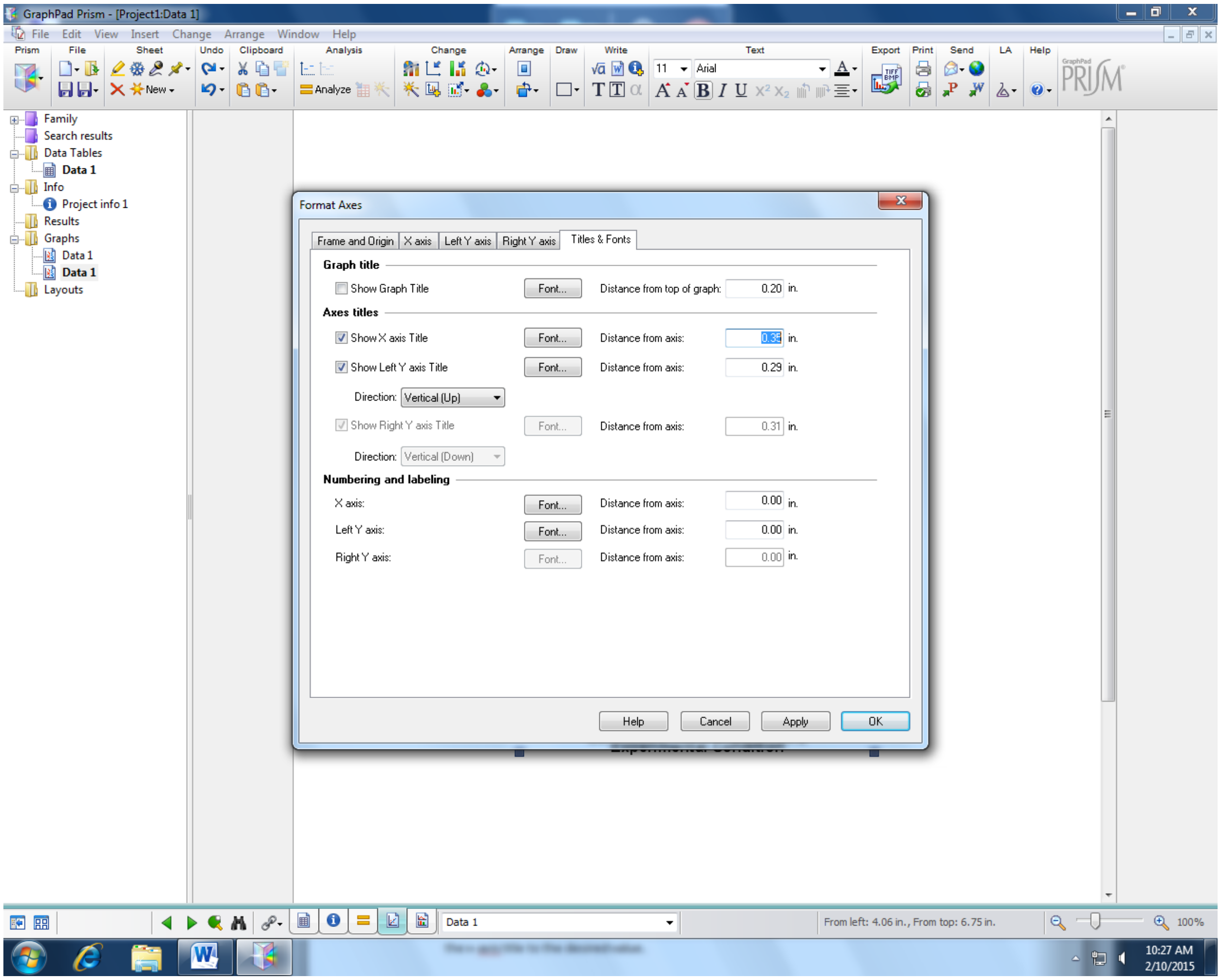

Downloaded from: Weissgerber TL, Milic NM, Winham SJ, Garovic VD. Beyond Bar and Line Graphs: Time for a New Data Presentation Paradigm. *PLOS Biology*, 2015:13.

11. Your univariate scatterplot will look something like this.

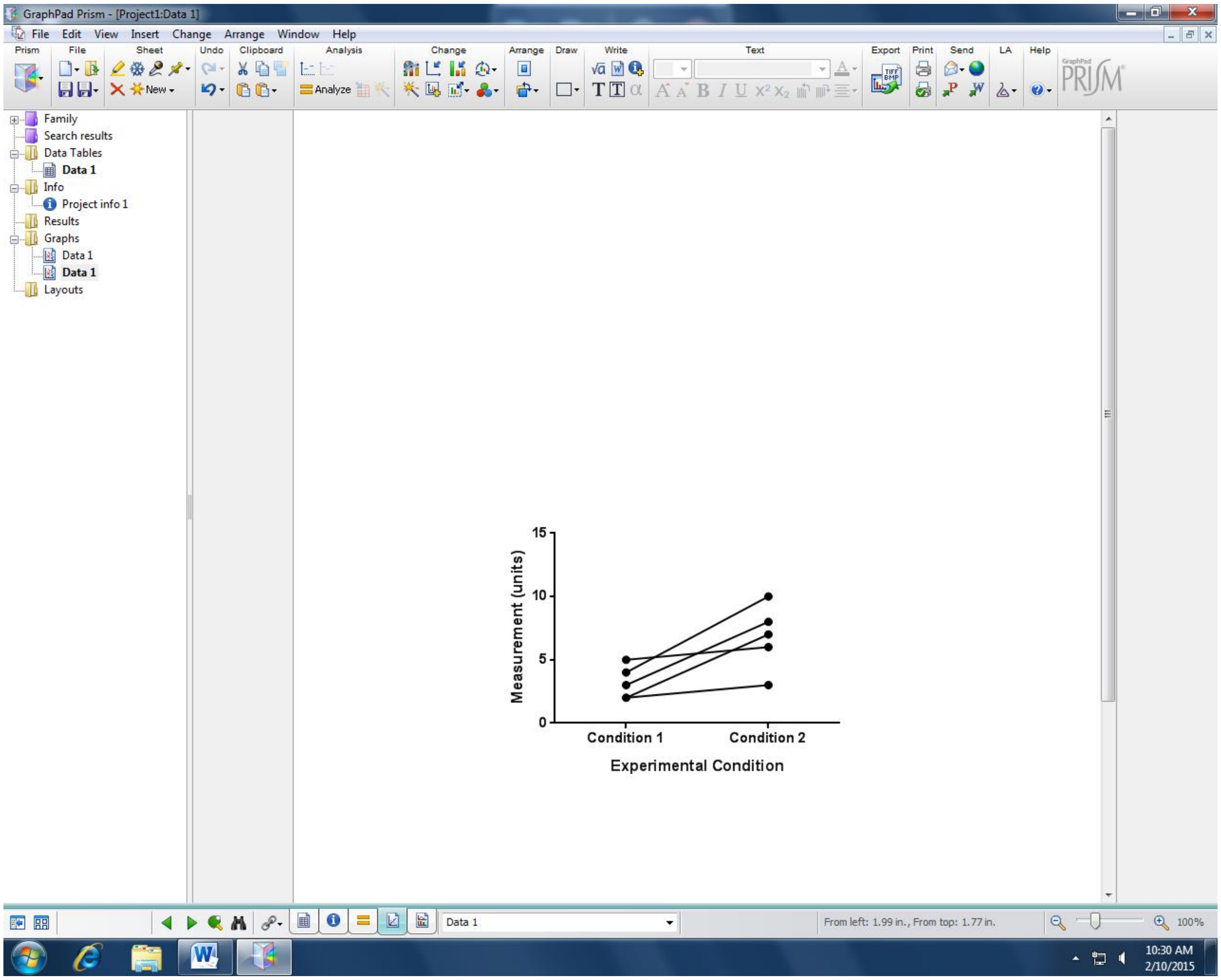

Downloaded from: Weissgerber TL, Milic NM, Winham SJ, Garovic VD. Beyond Bar and Line Graphs: Time for a New Data Presentation Paradigm. *PLOS Biology*, 2015:13.

12. Select "File", then "Export" to save the scatterplot.

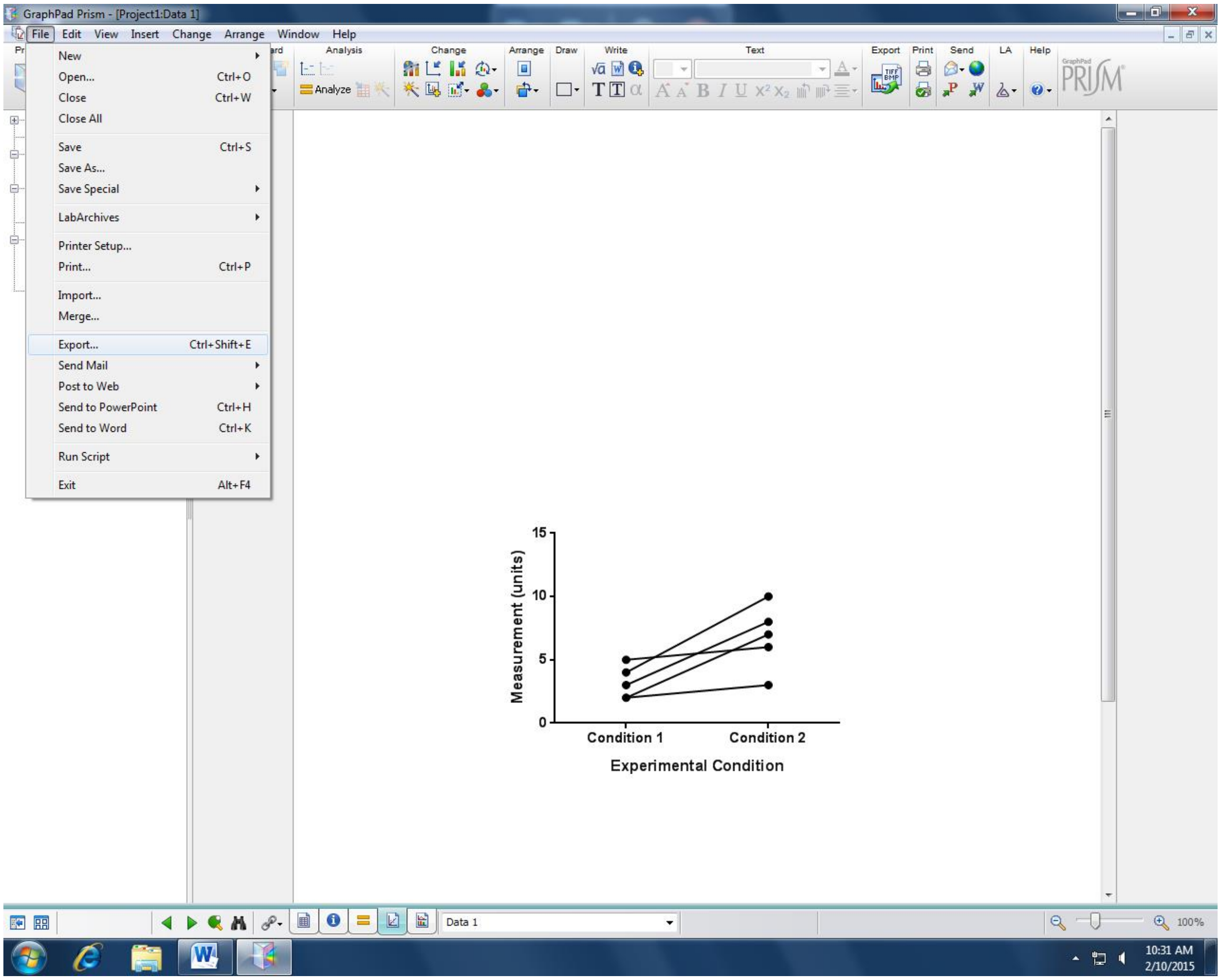

Downloaded from: Weissgerber TL, Milic NM, Winham SJ, Garovic VD. Beyond Bar and Line Graphs: Time for a New Data Presentation Paradigm. *PLOS Biology*, 2015:13.

13. Select the file format, enter a file name and chose the location where you want to save the graph. Click “OK”. Your scatterplot will be saved in the format and location that you selected.

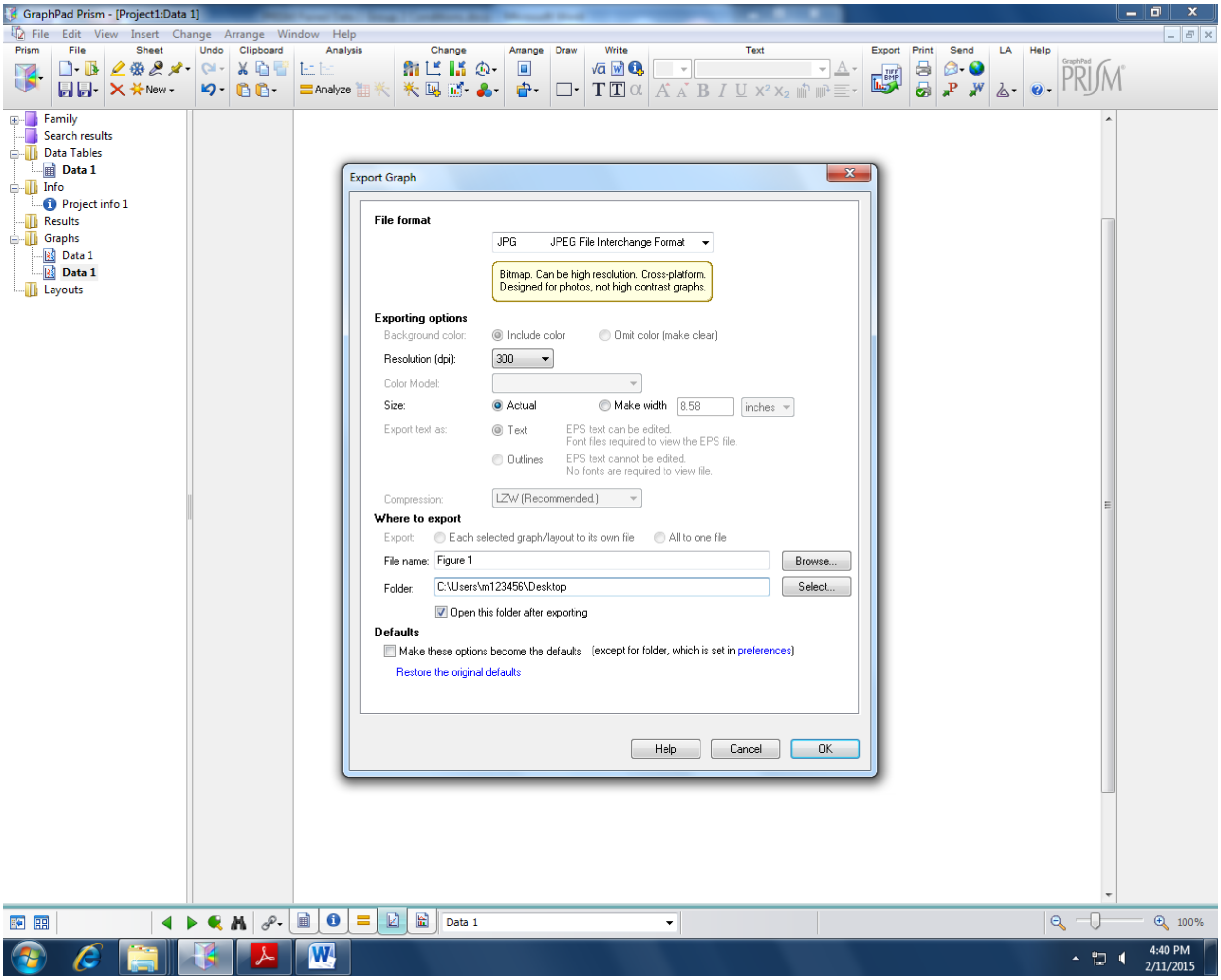

Downloaded from: Weissgerber TL, Milic NM, Winham SJ, Garovic VD. Beyond Bar and Line Graphs: Time for a New Data Presentation Paradigm. *PLOS Biology*, 2015:13.

14. The next step is to make a univariate scatterplot of the differences between Condition 1 and Condition 2. In the "Analysis" menu at the top of the screen, click on the "Analyze" symbol with the two yellow bars. In the pop-up menu, select "t-tests (and non-parametric tests)". Make sure Condition 1 and Condition 2 are both checked. Click "OK".

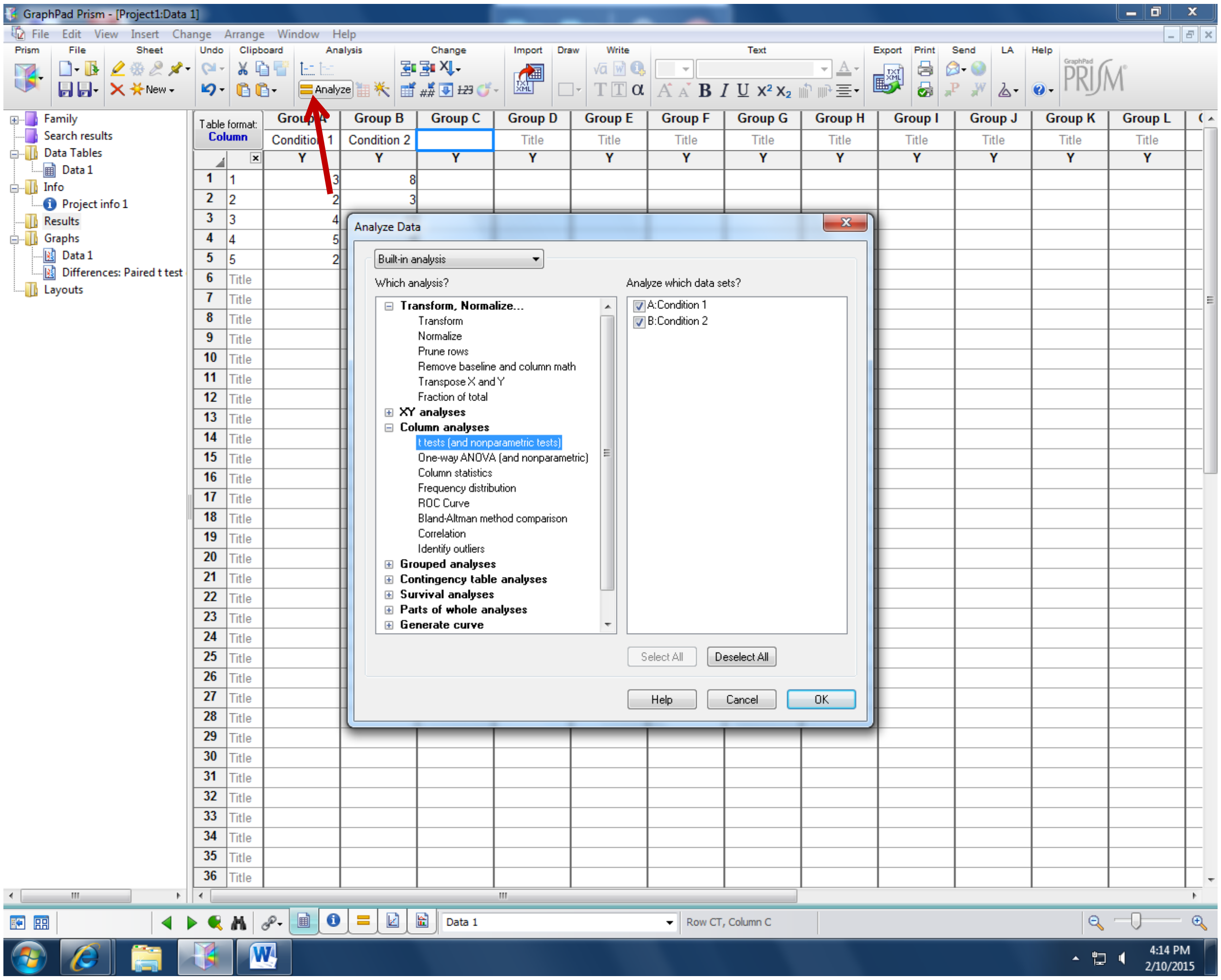

Downloaded from: Weissgerber TL, Milic NM, Winham SJ, Garovic VD. Beyond Bar and Line Graphs: Time for a New Data Presentation Paradigm. *PLOS Biology*, 2015:13.

15. In the next pop-up menu, made sure that “paired” is selected. You can adjust other options depending on whether you want the results of the statistical tests. For the purposes of graphing the differences, any test is fine.

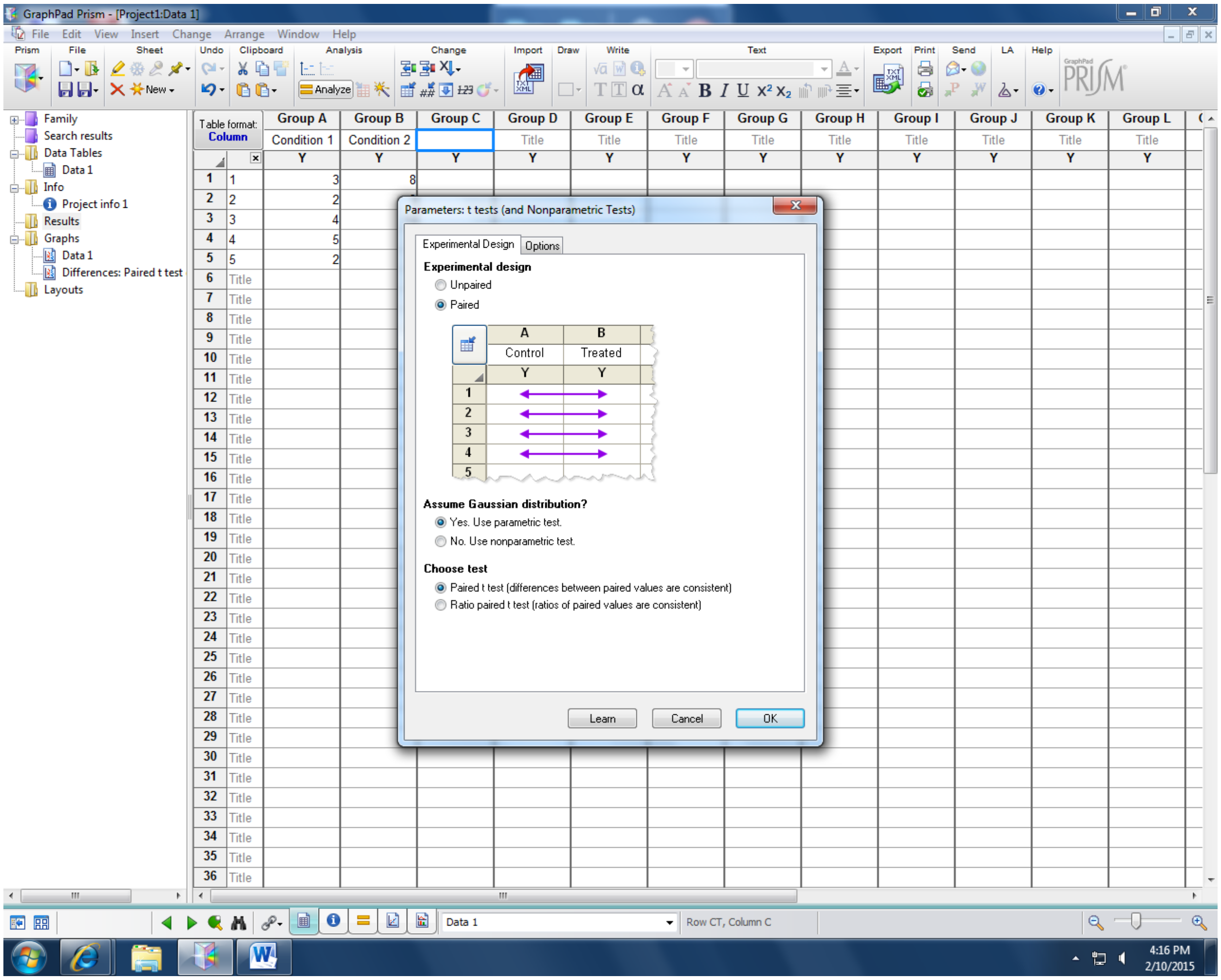

Downloaded from: Weissgerber TL, Milic NM, Winham SJ, Garovic VD. Beyond Bar and Line Graphs: Time for a New Data Presentation Paradigm. *PLOS Biology*, 2015:13.

16. Click on the “Options” tab. Select “Graph Differences (paired)”. Click “OK”.

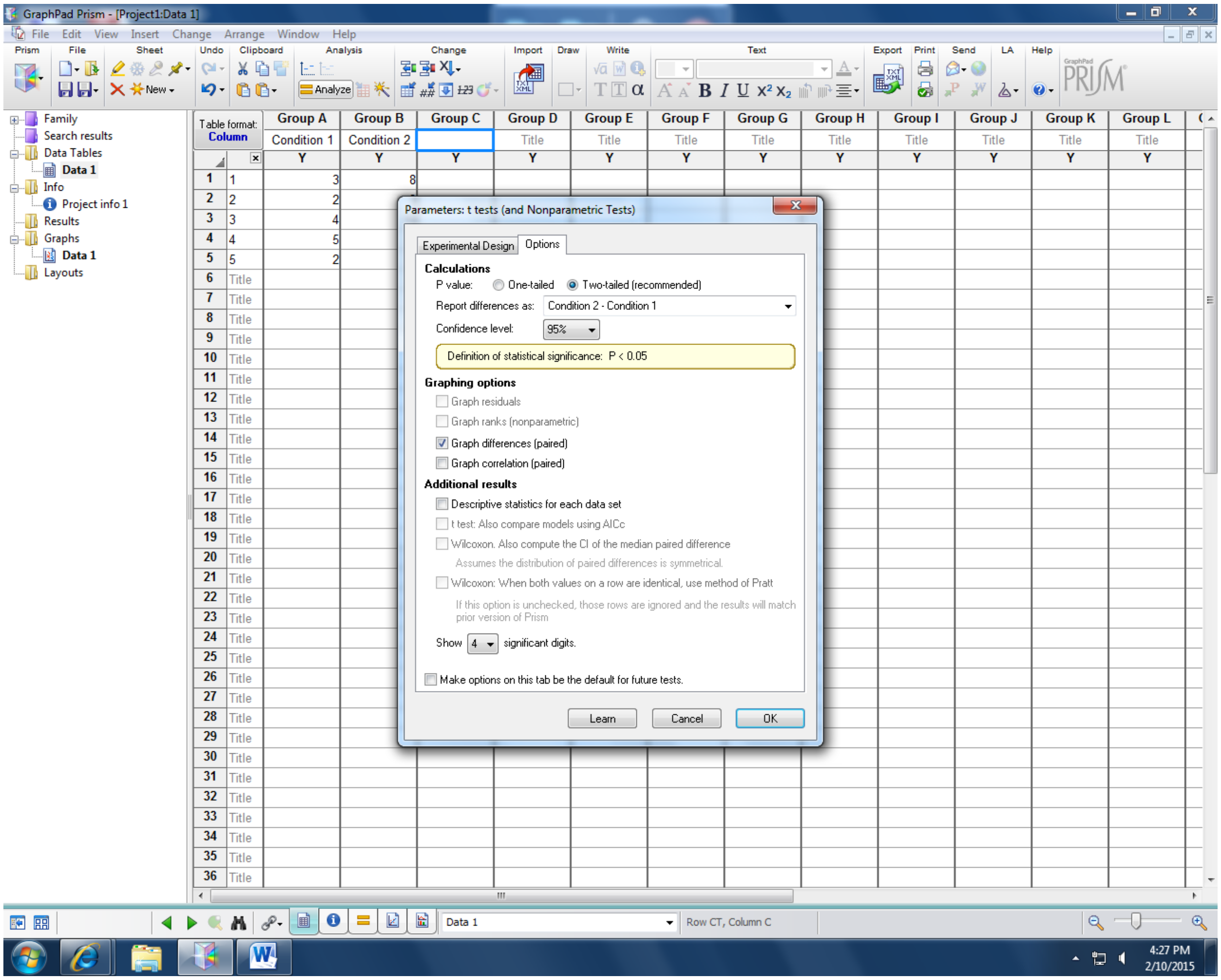

Downloaded from: Weissgerber TL, Milic NM, Winham SJ, Garovic VD. Beyond Bar and Line Graphs: Time for a New Data Presentation Paradigm. *PLOS Biology*, 2015:13.

17. The screen will show the analysis tab listing the results of the statistical test. The "Analysis" tab with two yellow lines will be highlighted at the bottom of the screen. To see the graph of differences, select the "View and Modify Graphs" tab next to the "Analysis" tab.

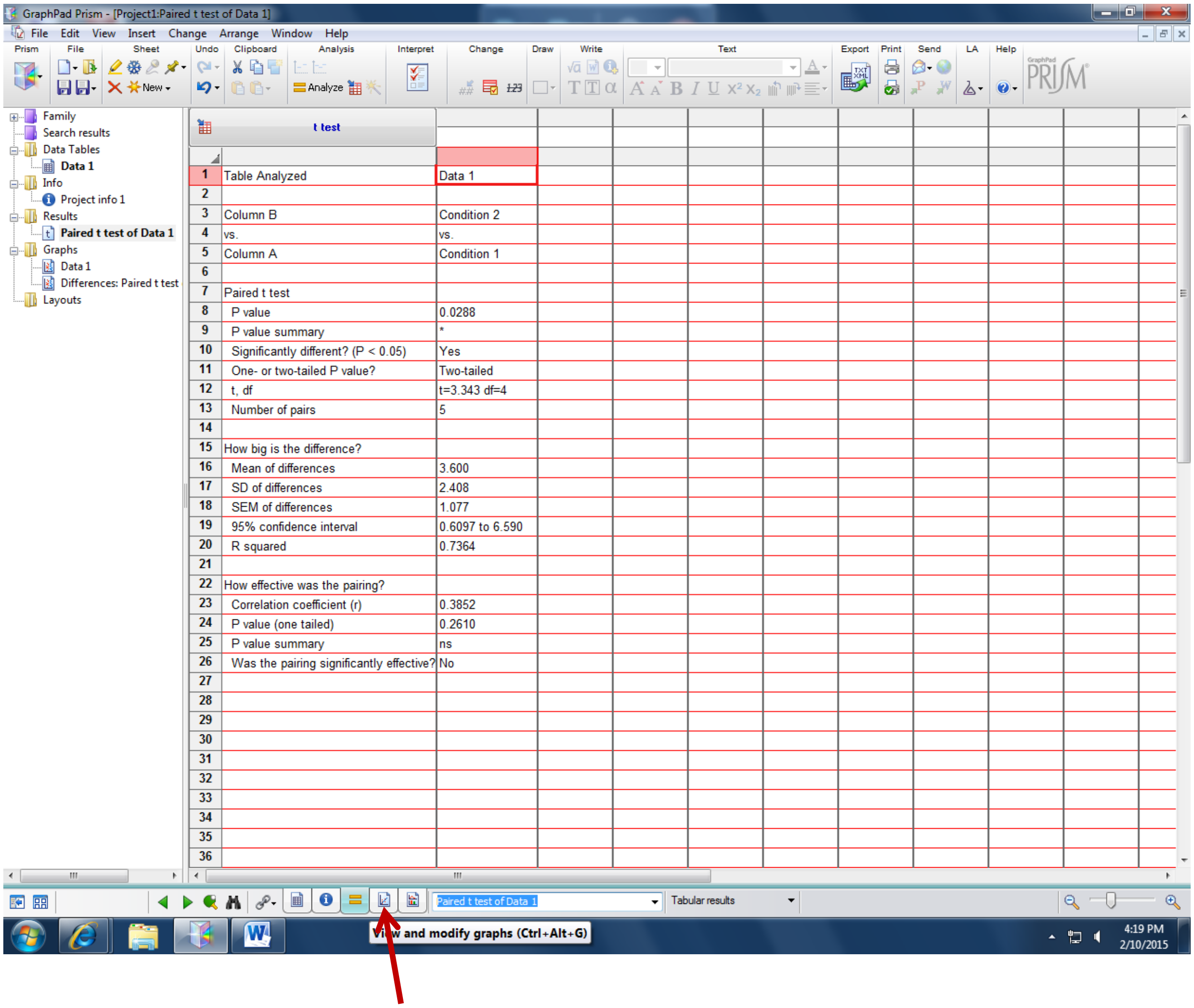

Downloaded from: Weissgerber TL, Milic NM, Winham SJ, Garovic VD. Beyond Bar and Line Graphs: Time for a New Data Presentation Paradigm. *PLOS Biology*, 2015:13.

18. This will show a graph of the difference scores. Click on "Difference" and type in the label that you would like to show.

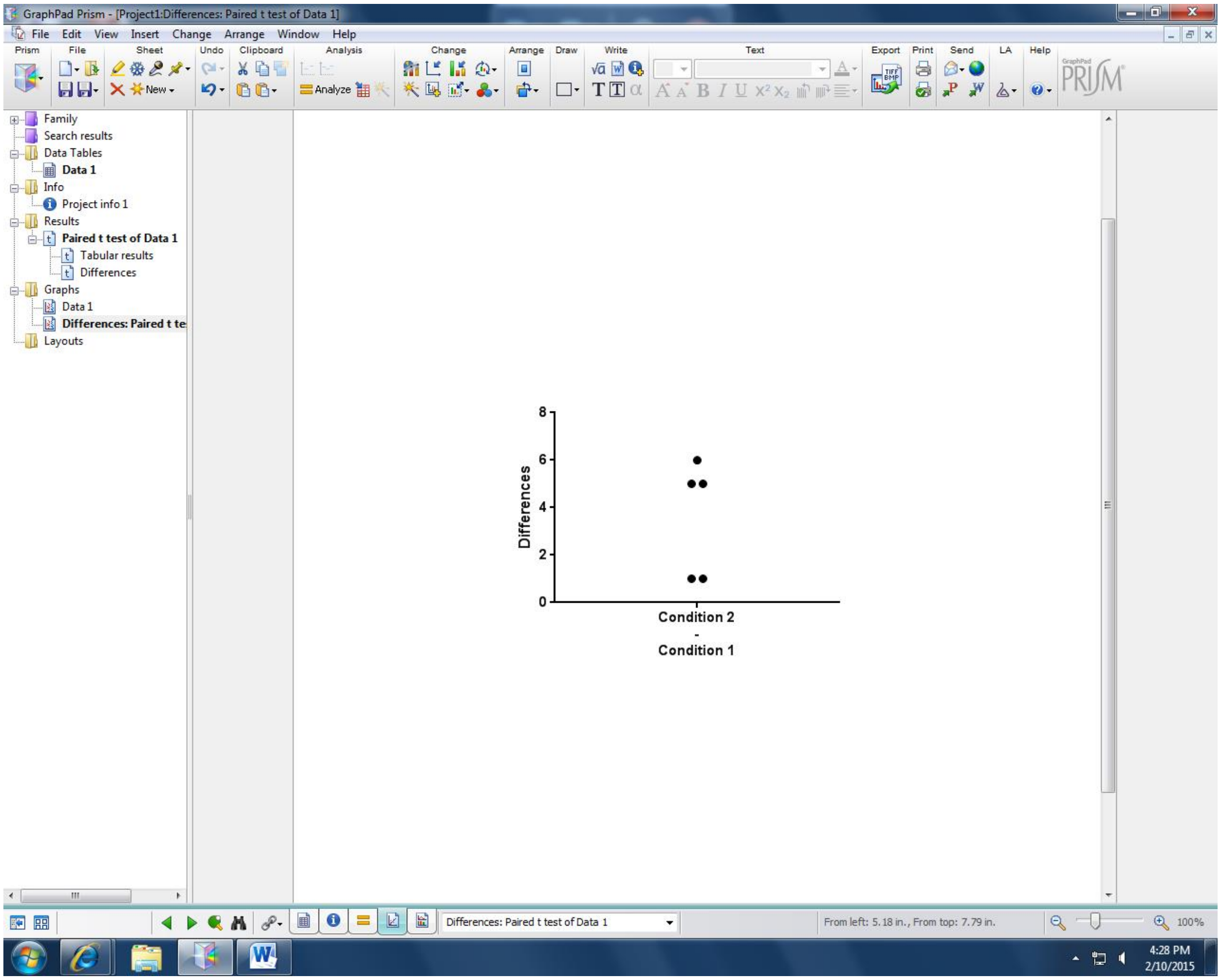

Downloaded from: Weissgerber TL, Milic NM, Winham SJ, Garovic VD. Beyond Bar and Line Graphs: Time for a New Data Presentation Paradigm. *PLOS Biology*, 2015:13.

19. Right click on the x-axis label. Select “Format axes” from the pop-up menu.

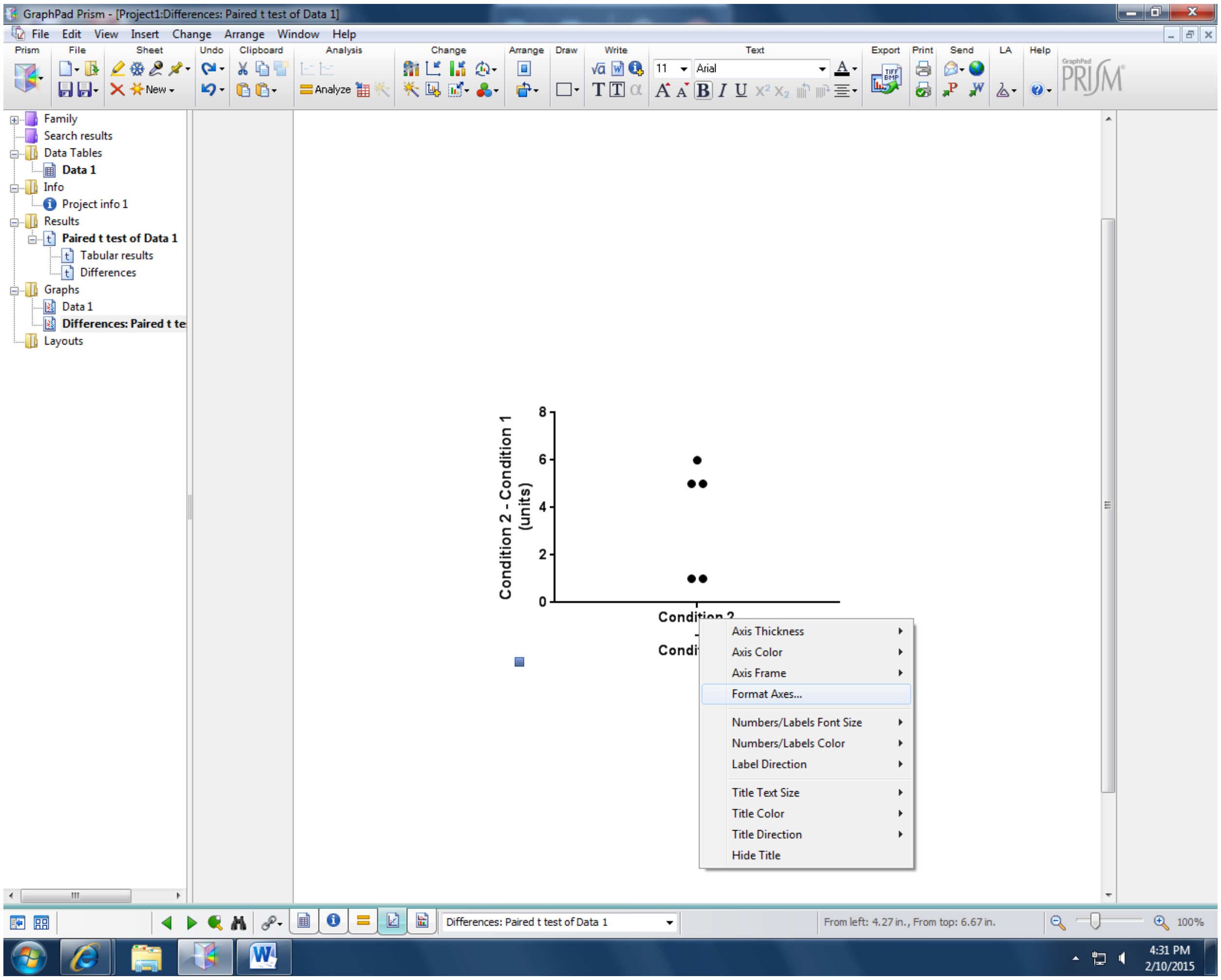

Downloaded from: Weissgerber TL, Milic NM, Winham SJ, Garovic VD. Beyond Bar and Line Graphs: Time for a New Data Presentation Paradigm. *PLOS Biology*, 2015:13.

20. Under “Location of numbering/labeling”, select “None”. Click “OK”.

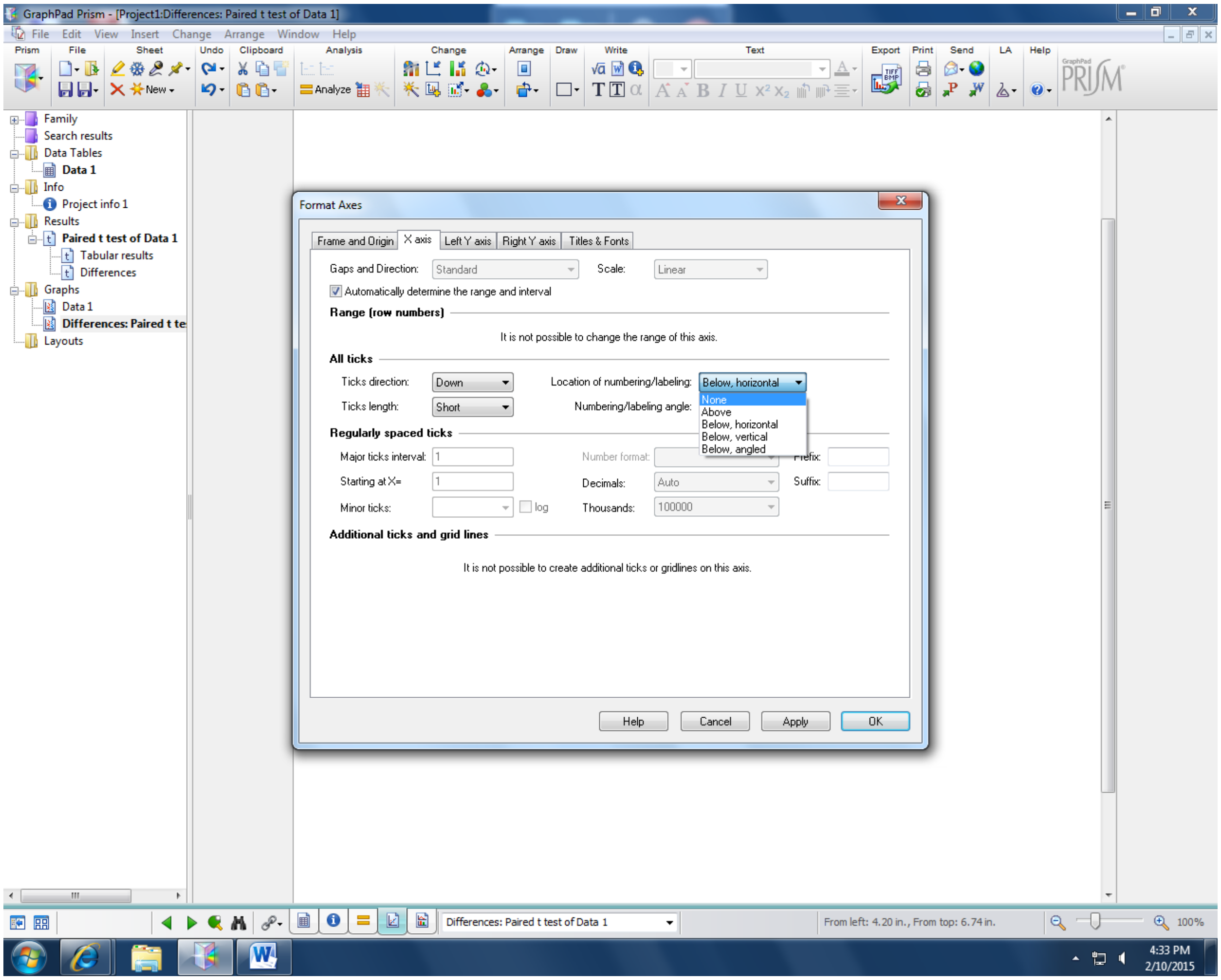

Downloaded from: Weissgerber TL, Milic NM, Winham SJ, Garovic VD. Beyond Bar and Line Graphs: Time for a New Data Presentation Paradigm. *PLOS Biology*, 2015:13.

21. In the “Change” menu at the top of the screen, select “Format graph (symbols, bars, error bars, etc.”.

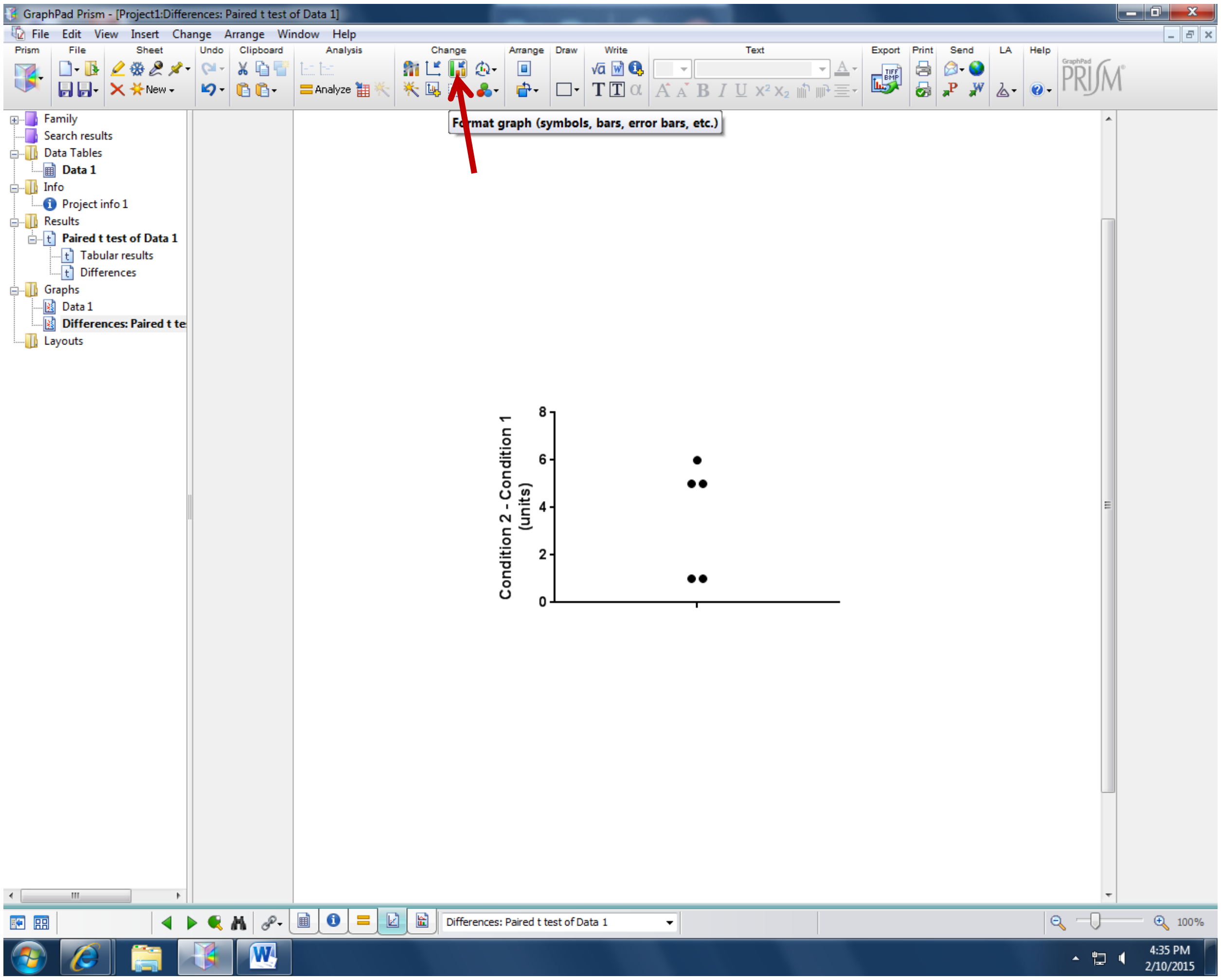

Downloaded from: Weissgerber TL, Milic NM, Winham SJ, Garovic VD. Beyond Bar and Line Graphs: Time for a New Data Presentation Paradigm. *PLOS Biology*, 2015:13.

22. In the pop-up menu, under “Line at” select “Median” if you are using non-parametric statistics or “Mean” if you are using parametric statistics. Under “Lines”, change length to “Short”. Click “OK”.

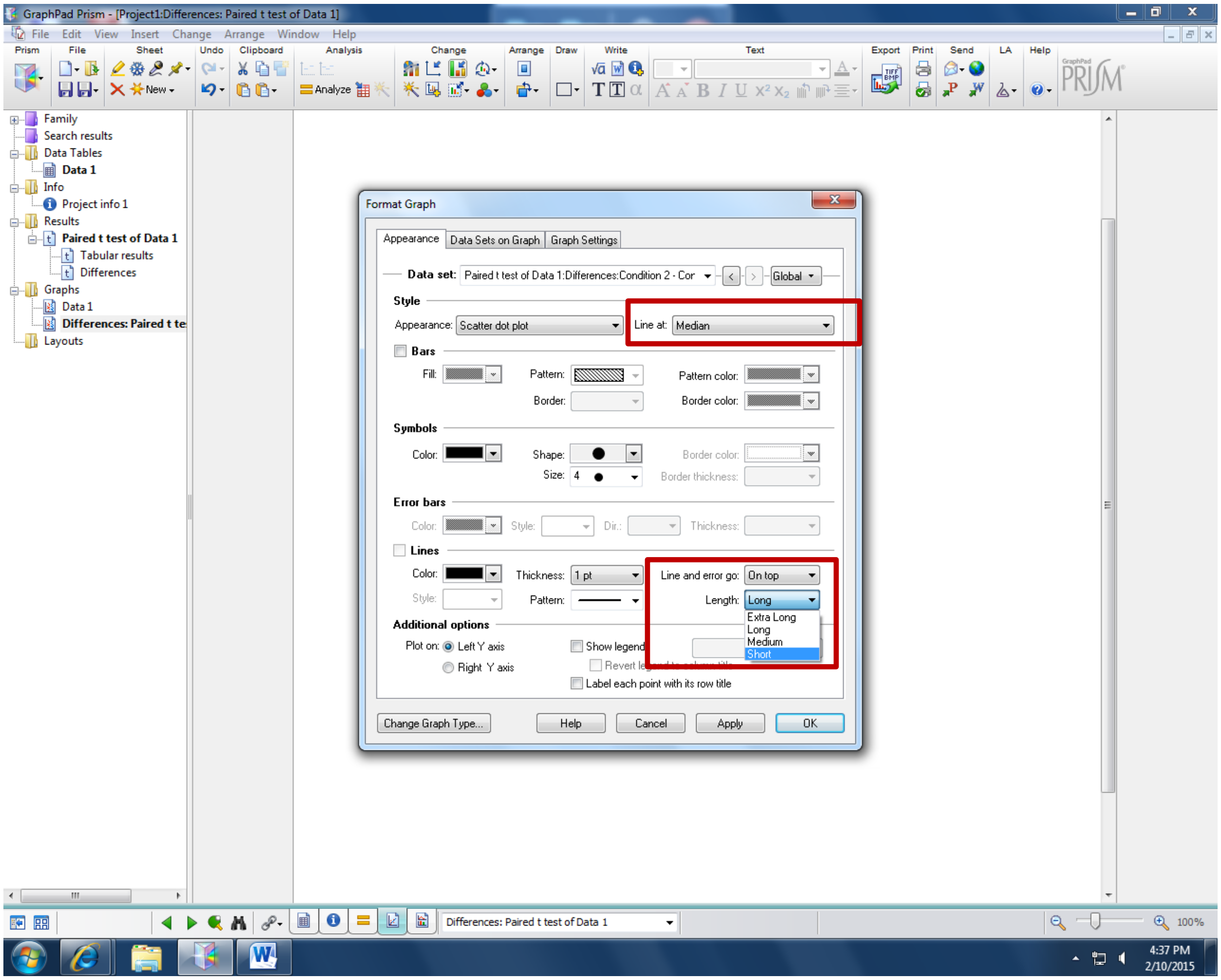

Downloaded from: Weissgerber TL, Milic NM, Winham SJ, Garovic VD. Beyond Bar and Line Graphs: Time for a New Data Presentation Paradigm. *PLOS Biology*, 2015:13.

23. Your graph should look something like this. Repeat steps 12 and 13 to save an image of your graph.

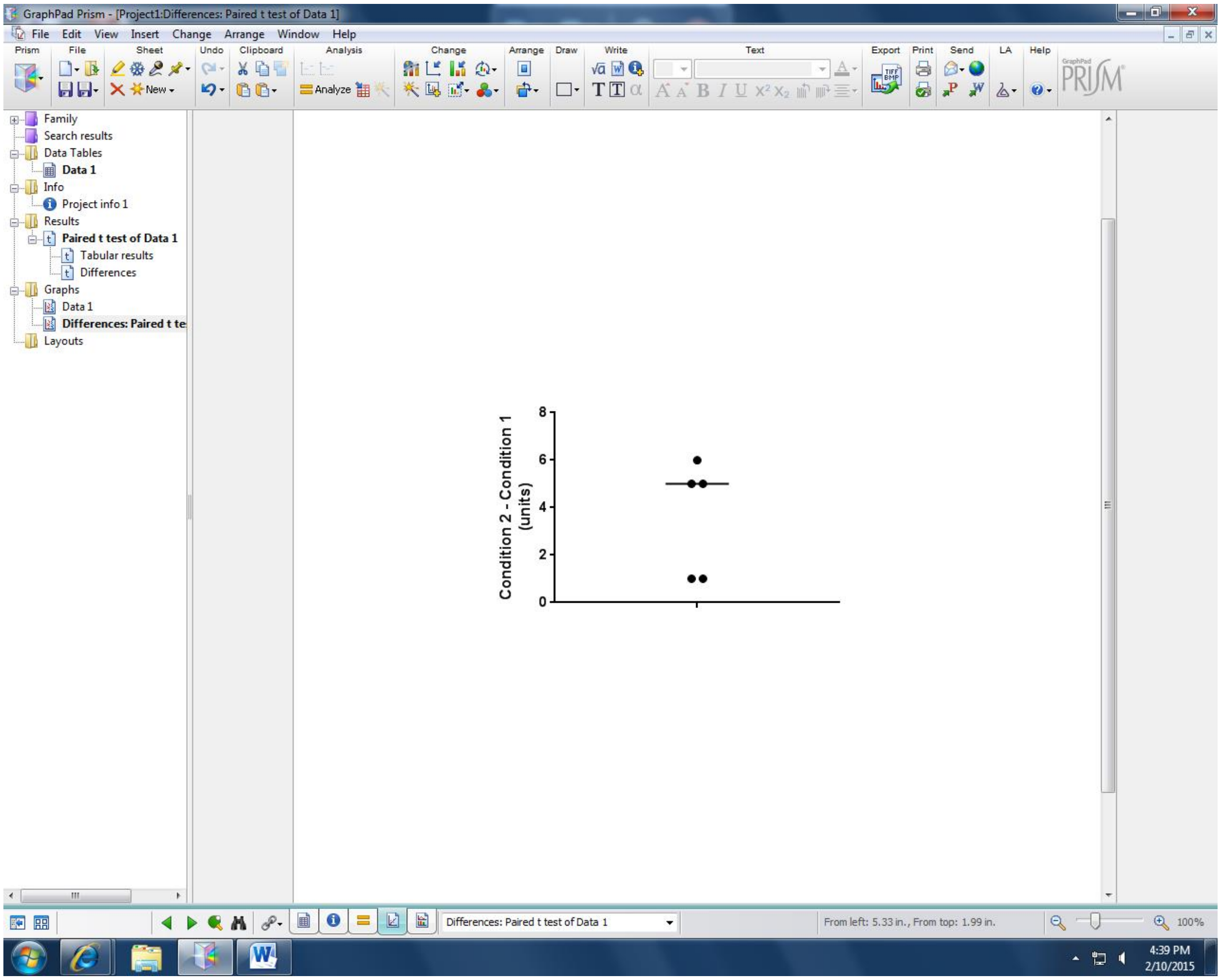

Downloaded from: Weissgerber TL, Milic NM, Winham SJ, Garovic VD. Beyond Bar and Line Graphs: Time for a New Data Presentation Paradigm. *PLOS Biology*, 2015:13.
