# Supplementary material for: Beyond Bar and Line Graphs: Time for a New Data Presentation Paradigm
Source: PLoS Biol. 2015 Apr 22;13(4):e1002128. doi: 10.1371/journal.pbio.1002128 (PMC4406565; doi:10.1371/journal.pbio.1002128)
Supplement: S6 Text — Use these instructions to create scatterplots for paired data (two conditions) in two groups of participants or specimens using GraphPad PRISM 6.0. Paired data are when you measure the variable of interest more than one time in each participant. If your data are independent, please see the instructions for Independent data. (PDF) [file pbio.1002128.s006.pdf]

How to Create a Univariate Scatterplots for Paired Data (2 groups, 2 conditions/group) in GraphPad PRISM 6

These instructions will allow you to make graphs like this:

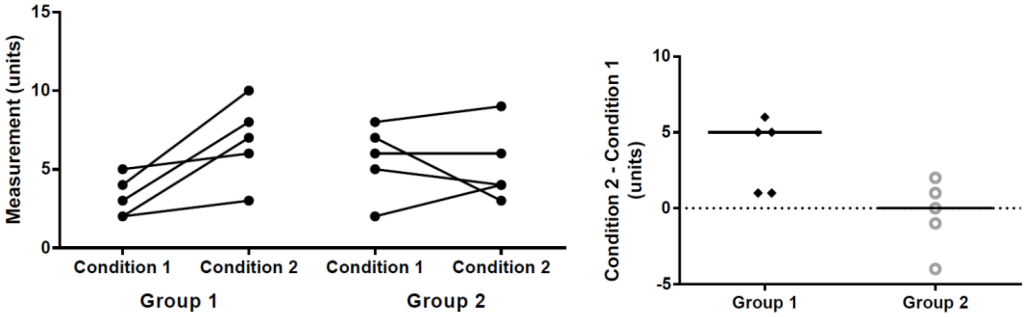

Use these instructions to create scatterplots for paired data (2 conditions) in two groups of subjects. Paired data are when you measure the variable of interest more than one time in each participant. If your data are independent, please see the instructions for Independent data.

Downloaded from: Weissgerber TL, Milic NM, Winham SJ, Garovic VD. Beyond Bar and Line Graphs: Time for a New Data Presentation Paradigm. *PLOS Biology*, 2015:13.

1. Under “New Table & Graph”, select “Column”
2. Select “Enter paired or repeated measures data – each subject on a separate row”. Click “Create”.

   *Note that these graphs can also be made using the “Grouped” option, however data entry is more complex.

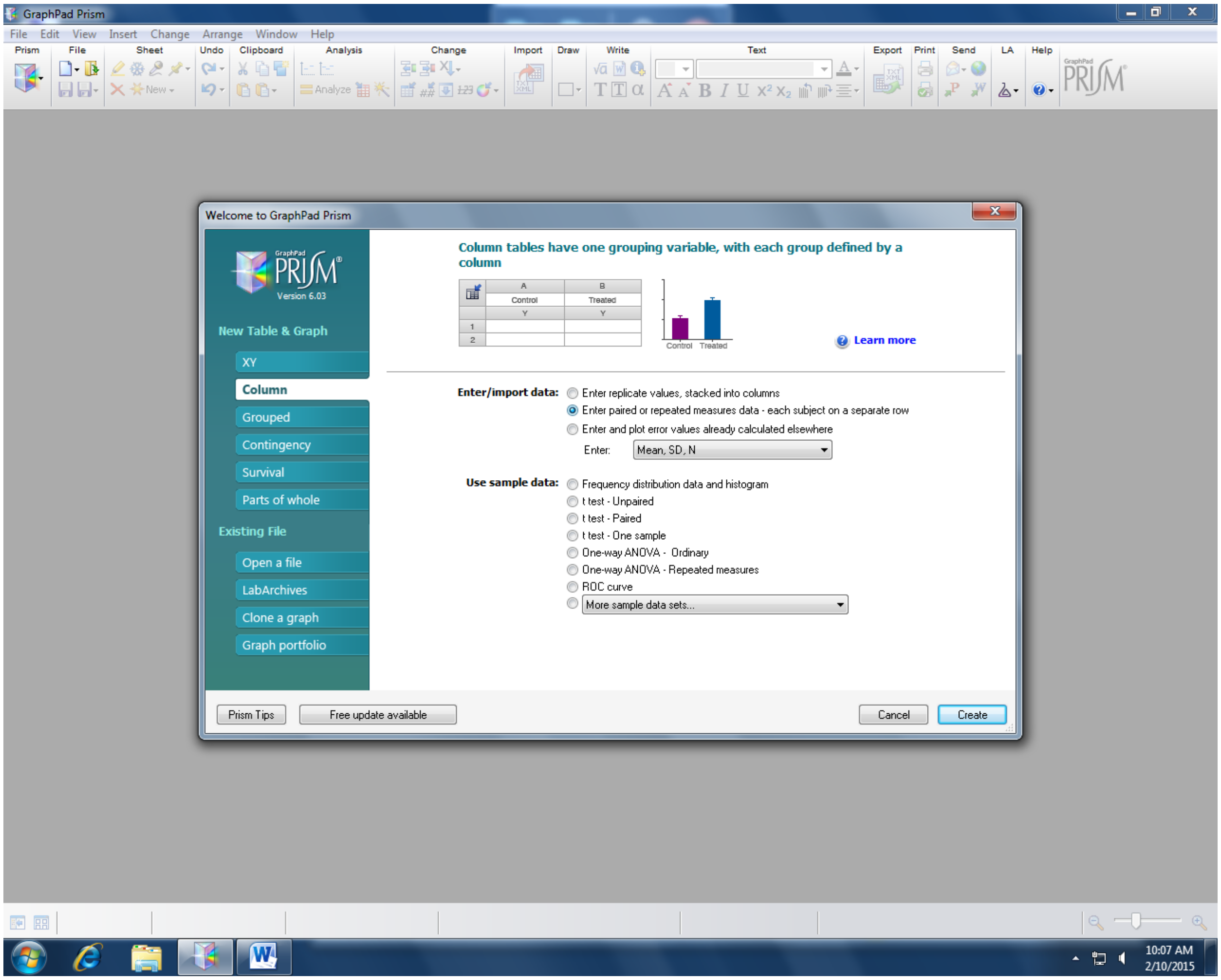

Downloaded from: Weissgerber TL, Milic NM, Winham SJ, Garovic VD. Beyond Bar and Line Graphs: Time for a New Data Presentation Paradigm. *PLOS Biology*, 2015:13.

3. Enter the name for Condition 1 just below “Group A” and “Group “C”. Enter the name for Condition 2 just below “Group B” and “Group D”. The condition names that you enter will appear as labels on your graph.
   Enter you data as follows (one row per subject or specimen):
   - First column (before “Group A” column): Subject or specimen ID
   - “Group A” column: Data from Group 1, Condition 1
   - “Group B” column: Data from Group 1, Condition 2
   - “Group C” column: Data from Group 2, Condition 1
   - “Group D” column: Data from Group 2, Condition 2

   Subjects from Group 1 will only have data in the “Group A” and Group B” columns, whereas subjects from Group 2 will only have data in the “Group C” and “Group D” columns.

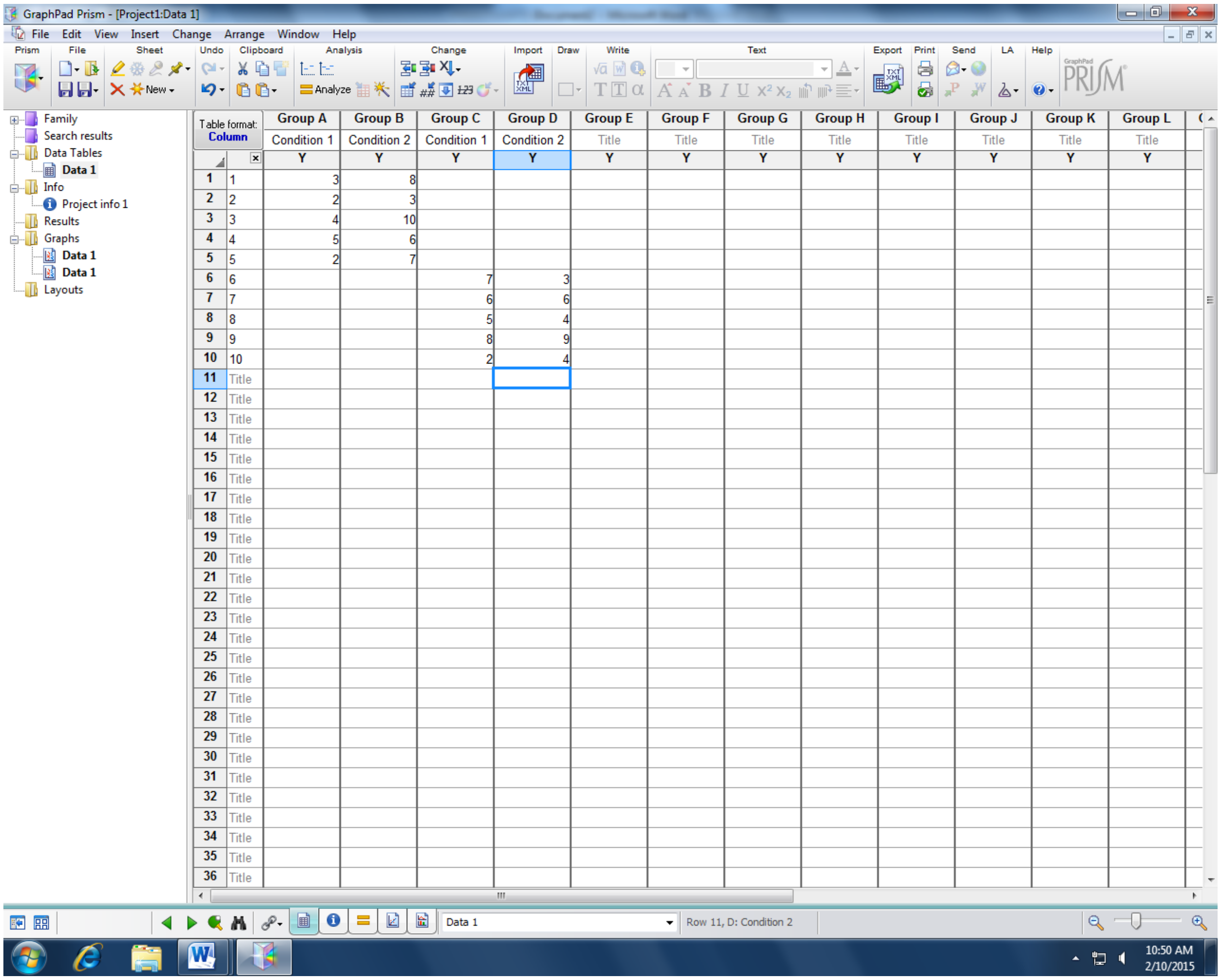

Downloaded from: Weissgerber TL, Milic NM, Winham SJ, Garovic VD. Beyond Bar and Line Graphs: Time for a New Data Presentation Paradigm. *PLOS Biology*, 2015:13.

4. From the insert menu at the top of the screen, select “New graph of existing data”

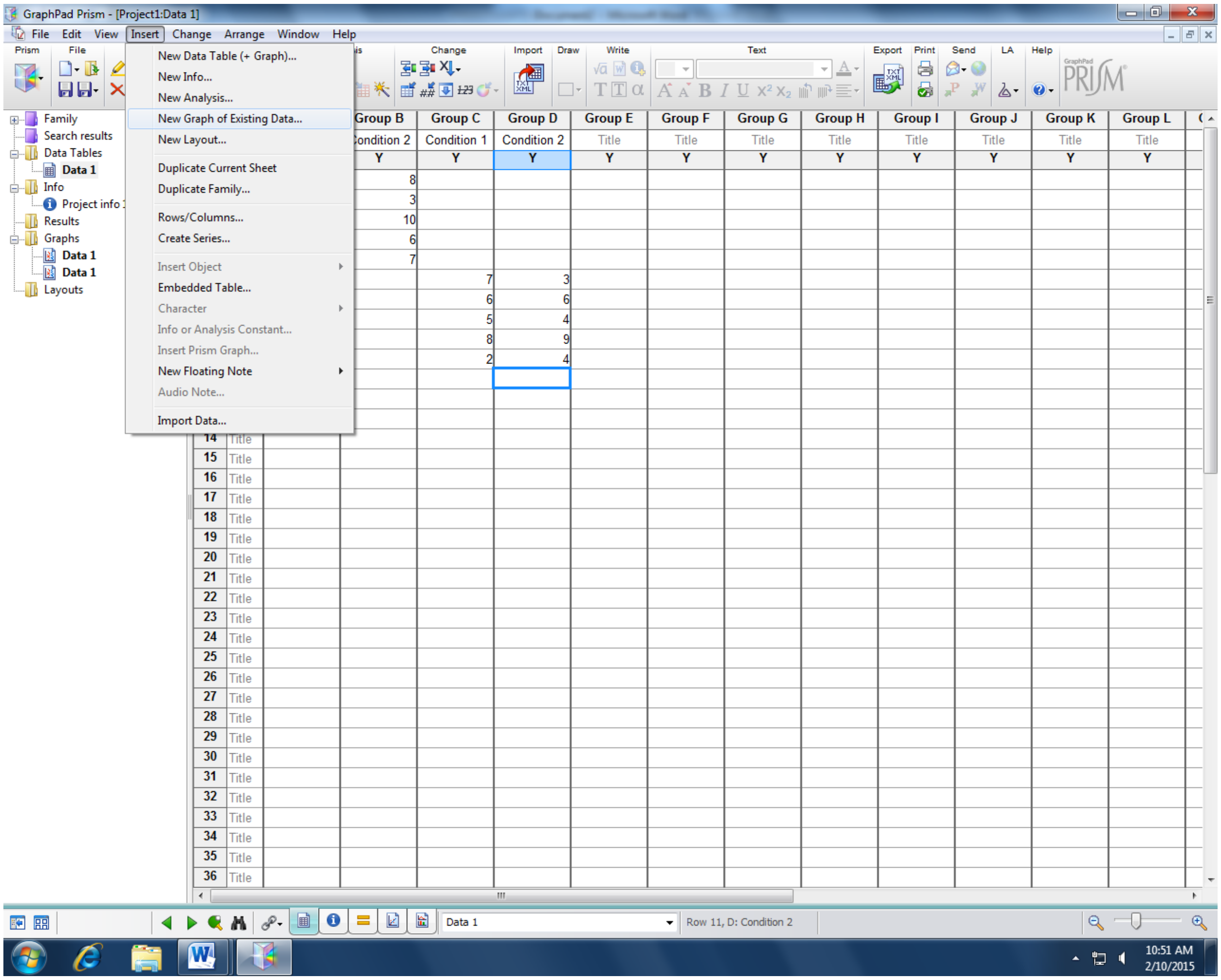

Downloaded from: Weissgerber TL, Milic NM, Winham SJ, Garovic VD. Beyond Bar and Line Graphs: Time for a New Data Presentation Paradigm. *PLOS Biology*, 2015:13.

5. Ensure that the “Symbols & lines” plot is selected. Click “OK” to create the graph.

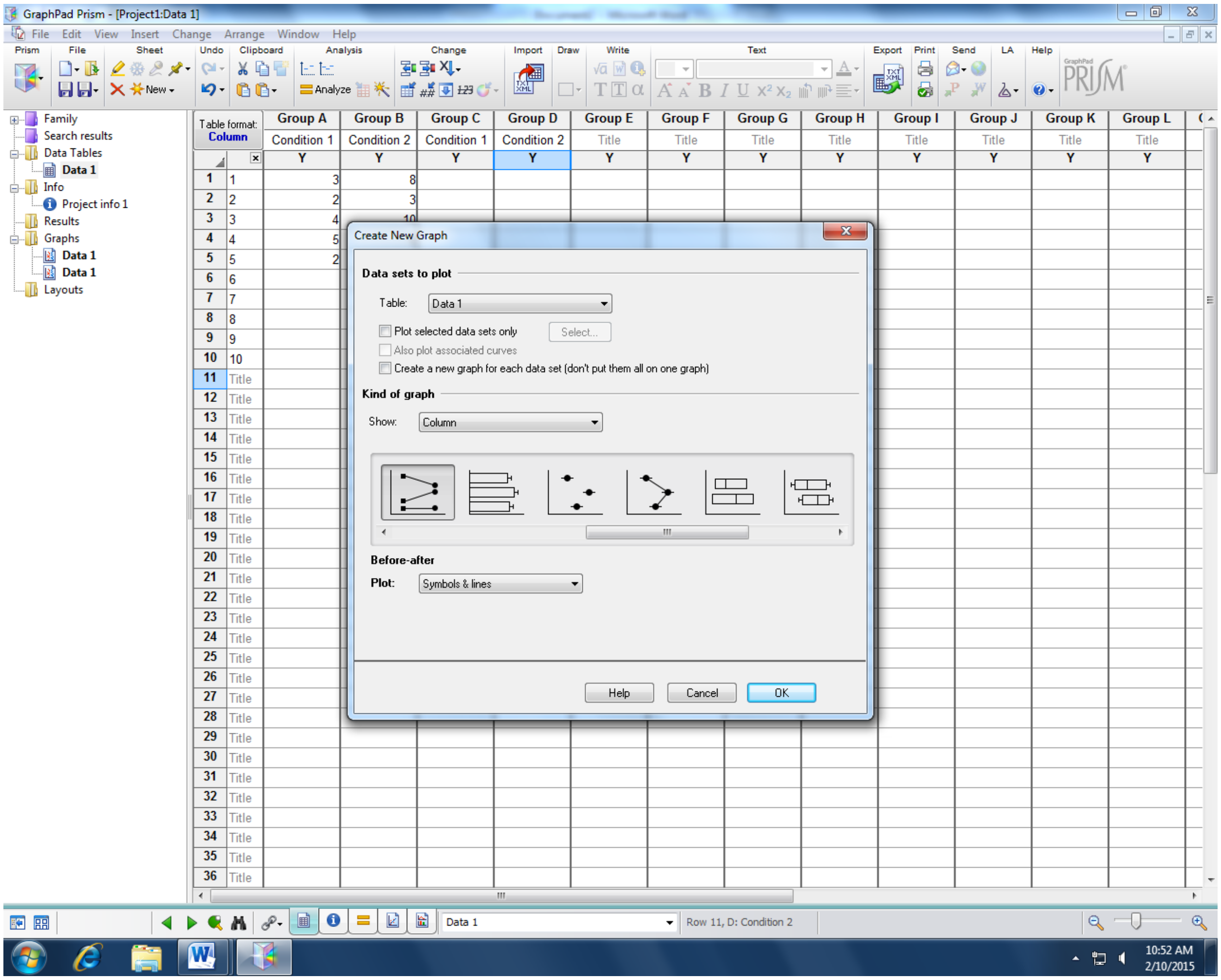

Downloaded from: Weissgerber TL, Milic NM, Winham SJ, Garovic VD. Beyond Bar and Line Graphs: Time for a New Data Presentation Paradigm. *PLOS Biology*, 2015:13.

6. Your graph will look something like this. Click on "YTitle" to replace this label with the names and units for your axis. Click on "XTitle" and replace this with the names for Group 1 and Group 2, using the spaces in between to center both labels. Delete the chart title (Data1 in this example) or click on the chart title to replace it with the desired text.

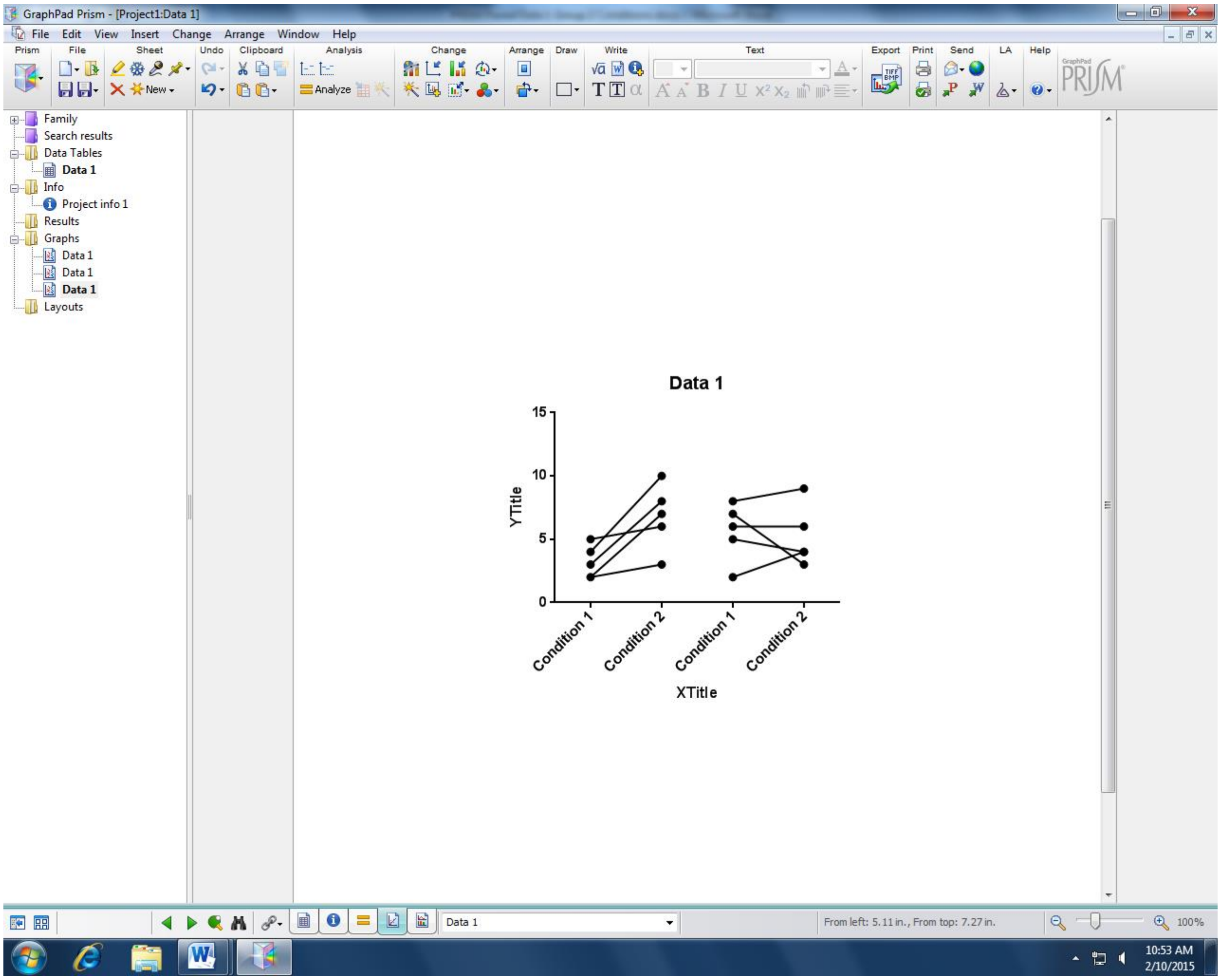

Downloaded from: Weissgerber TL, Milic NM, Winham SJ, Garovic VD. Beyond Bar and Line Graphs: Time for a New Data Presentation Paradigm. *PLOS Biology*, 2015:13.

7. Your new axis labels should appear on the graph.

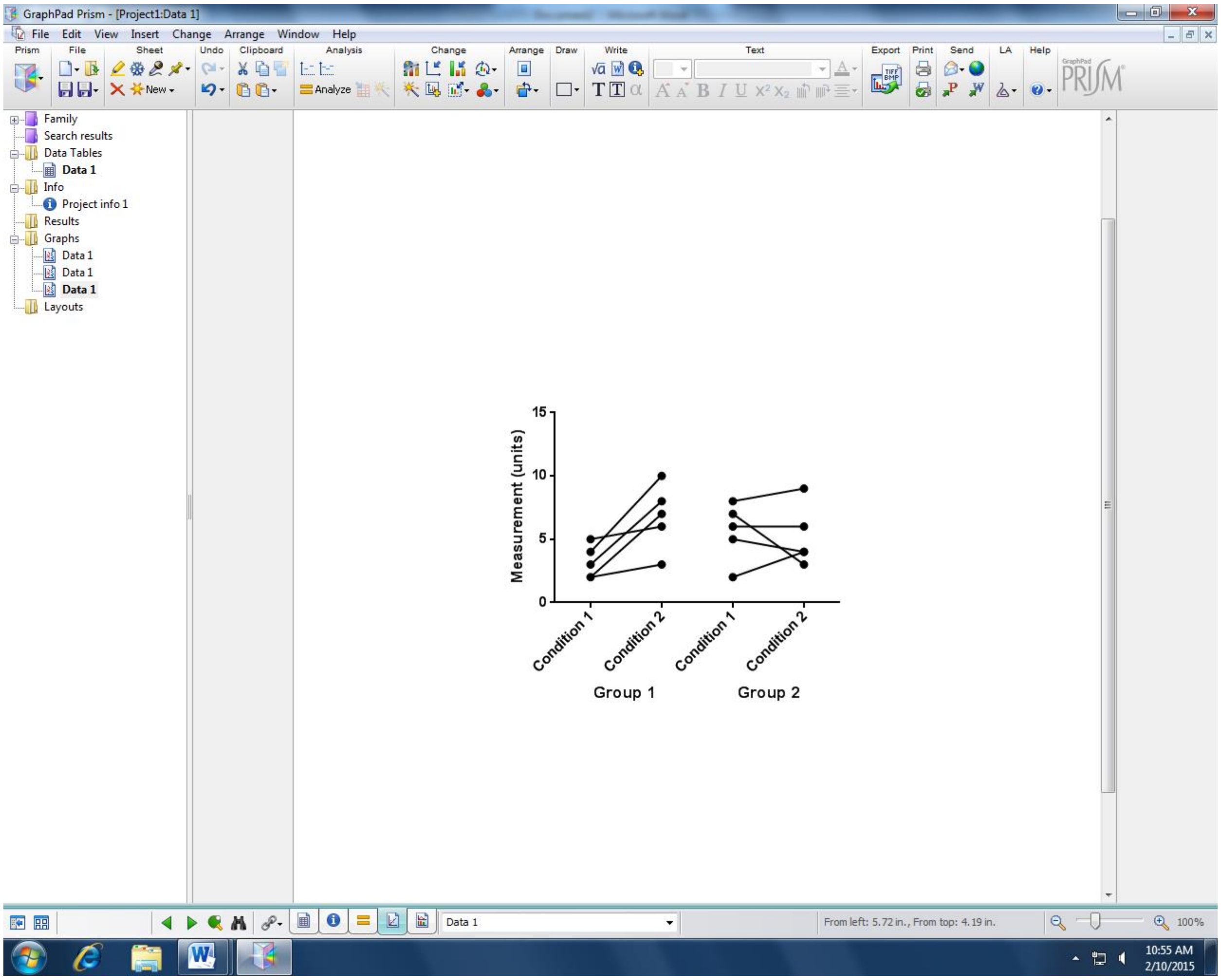

Downloaded from: Weissgerber TL, Milic NM, Winham SJ, Garovic VD. Beyond Bar and Line Graphs: Time for a New Data Presentation Paradigm. *PLOS Biology*, 2015:13.

8. Left click on one of the x-axis labels; then right click and select “Format axis”.

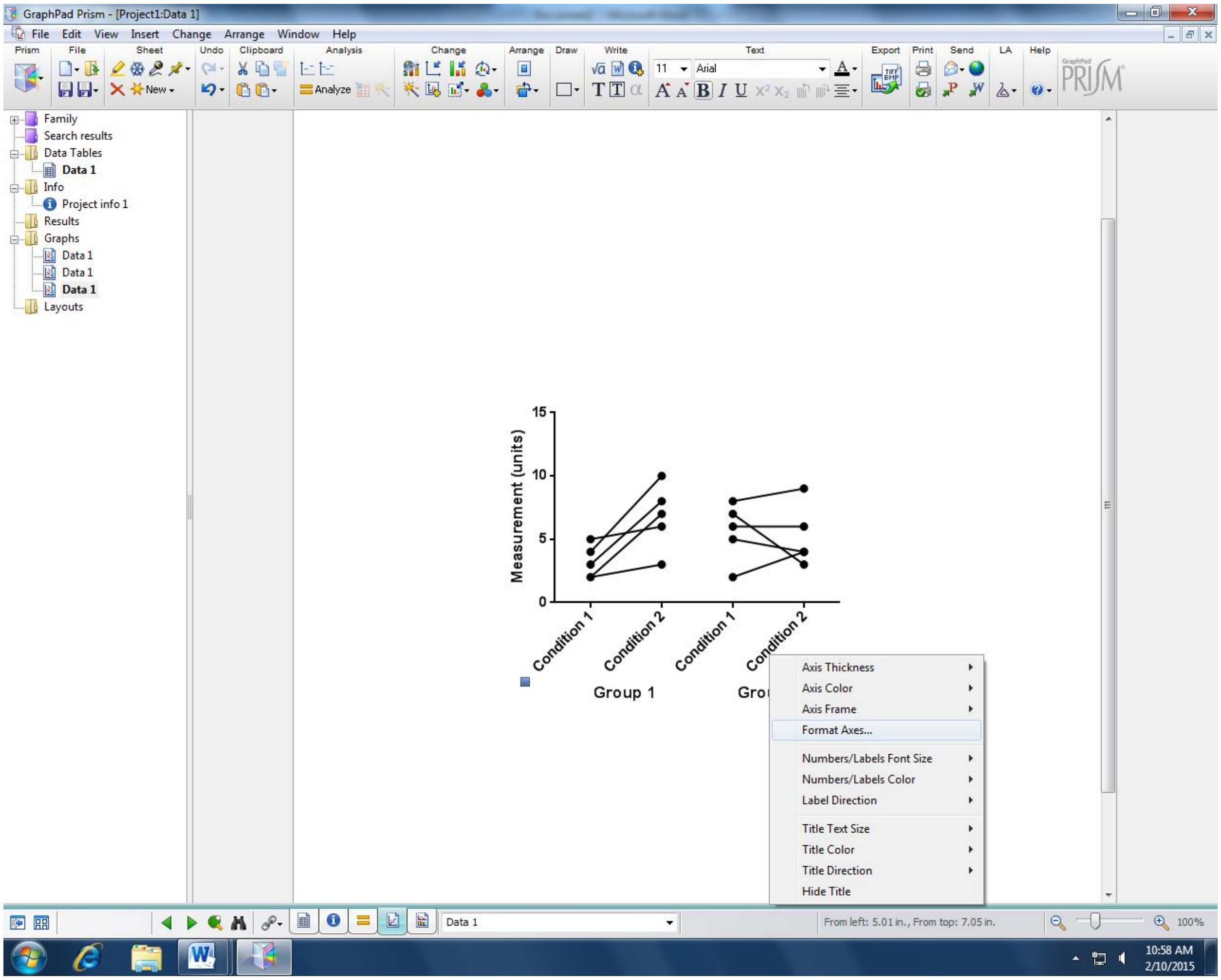

Downloaded from: Weissgerber TL, Milic NM, Winham SJ, Garovic VD. Beyond Bar and Line Graphs: Time for a New Data Presentation Paradigm. *PLOS Biology*, 2015:13.

9. In the pop-up menu, under the x-axis tab change “Below, angled” to “Below, horizontal”.

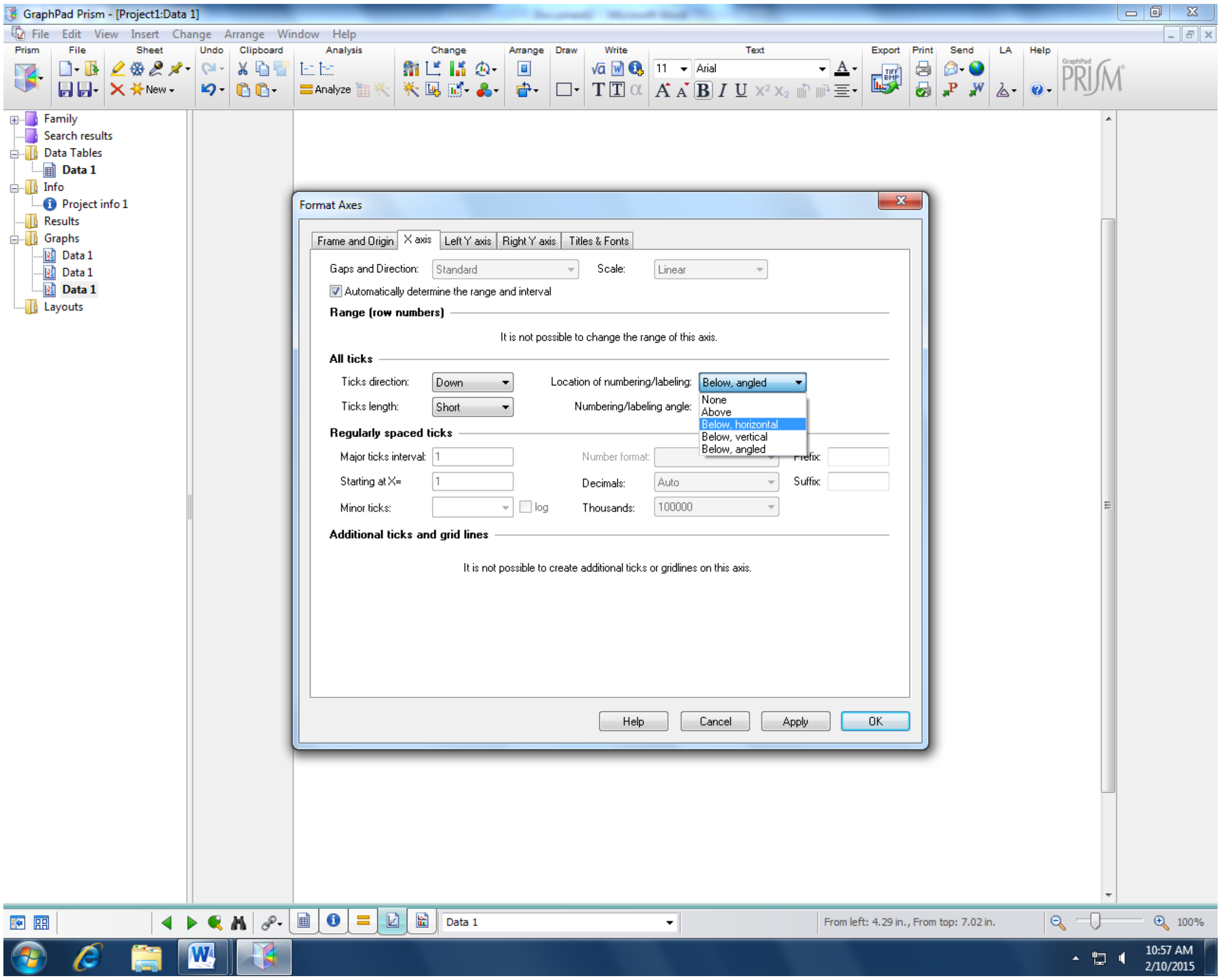

Downloaded from: Weissgerber TL, Milic NM, Winham SJ, Garovic VD. Beyond Bar and Line Graphs: Time for a New Data Presentation Paradigm. *PLOS Biology*, 2015:13.

10. Select the “Titles & Fonts” tab. Change ”Distance from axis” for the x-axis title to the desired value. Click “Apply” to view the change. Click “OK” when you are happy with the result.

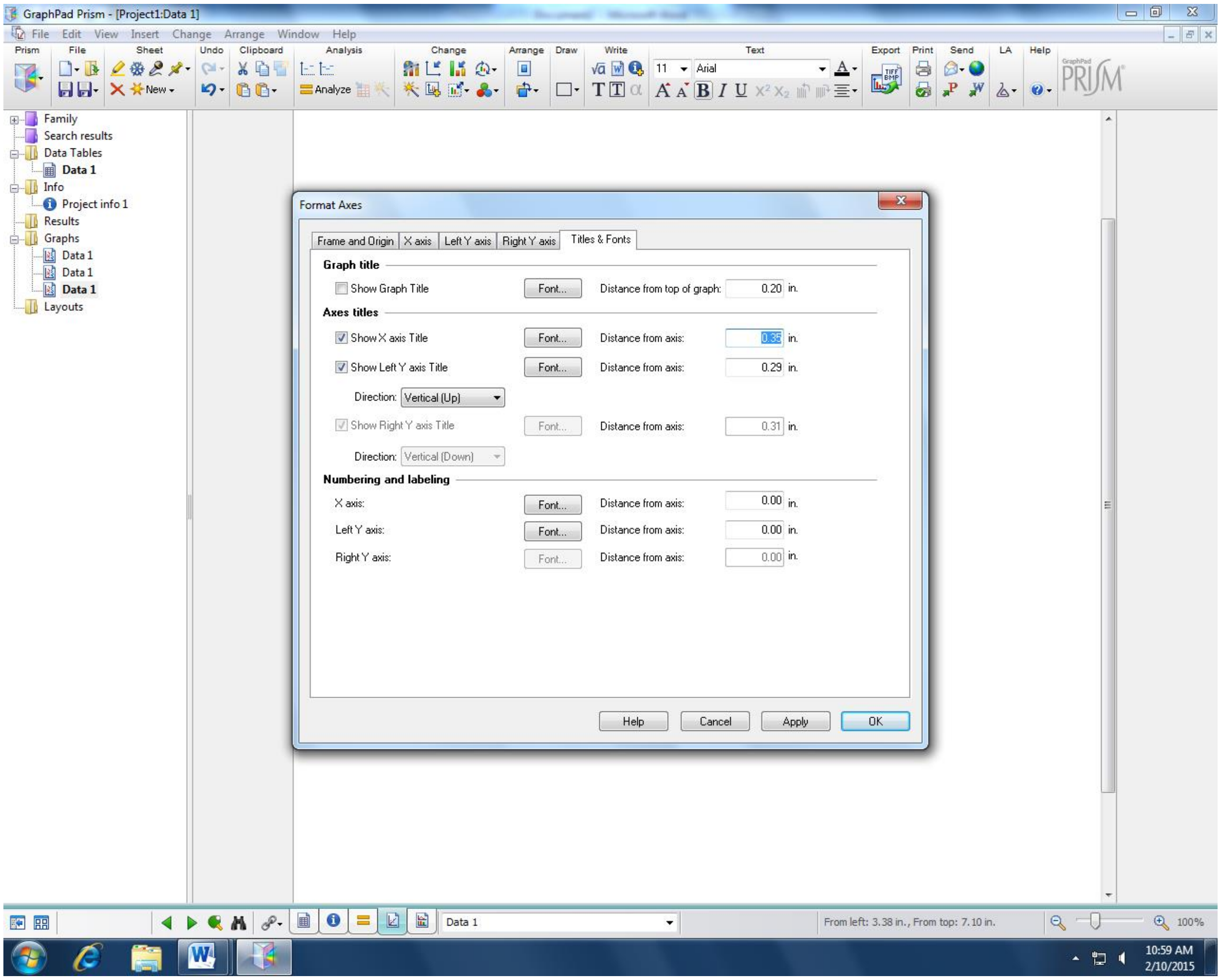

Downloaded from: Weissgerber TL, Milic NM, Winham SJ, Garovic VD. Beyond Bar and Line Graphs: Time for a New Data Presentation Paradigm. *PLOS Biology*, 2015:13.

11. Your graph will appear. Click on the right end of the x-axis and drag the blue box to extend the length of the axis as needed.

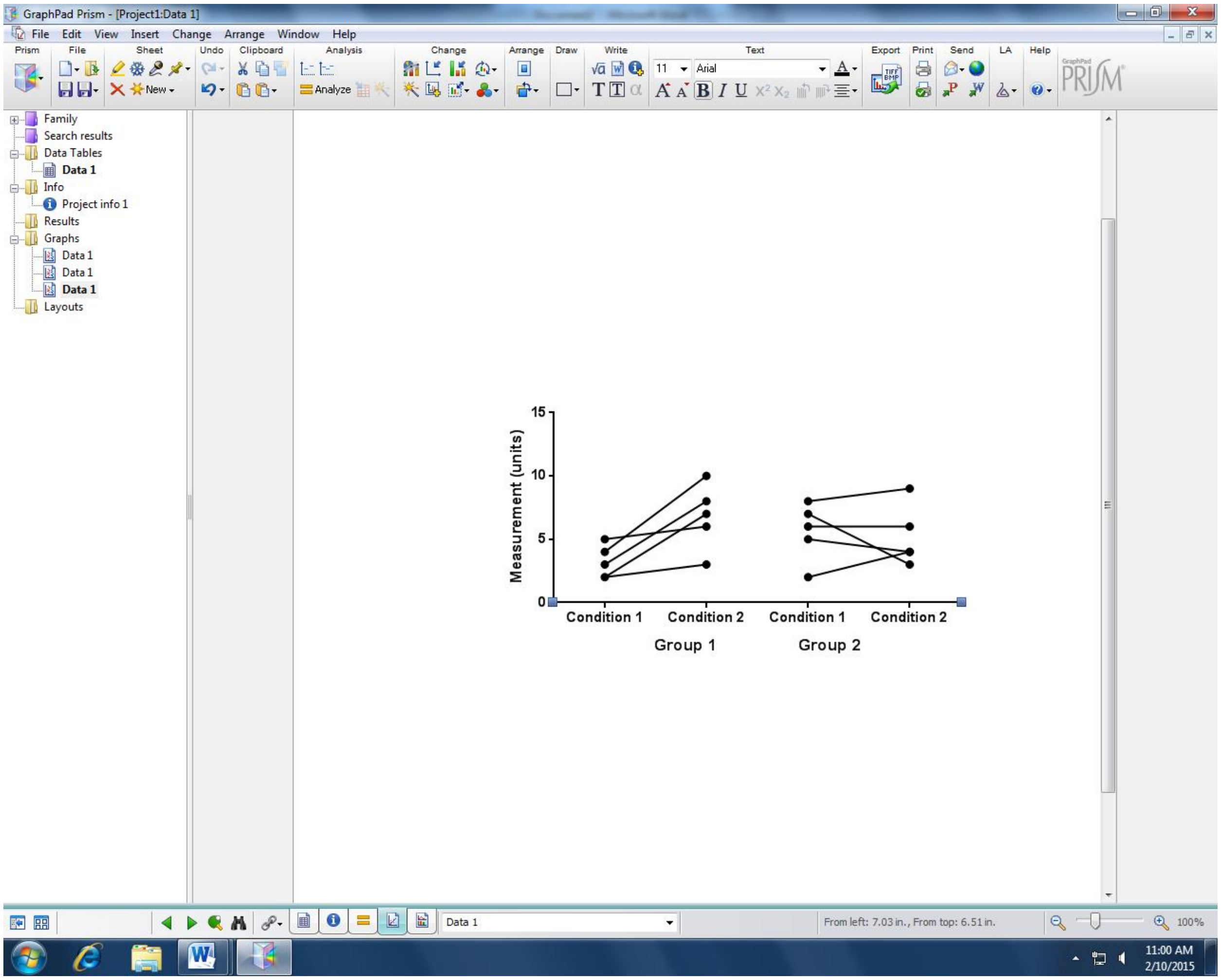

Downloaded from: Weissgerber TL, Milic NM, Winham SJ, Garovic VD. Beyond Bar and Line Graphs: Time for a New Data Presentation Paradigm. *PLOS Biology*, 2015:13.

12. Click on the Group labels on the x-axis and adjust the spacing as needed to center the Group 1 and Group 2 labels.

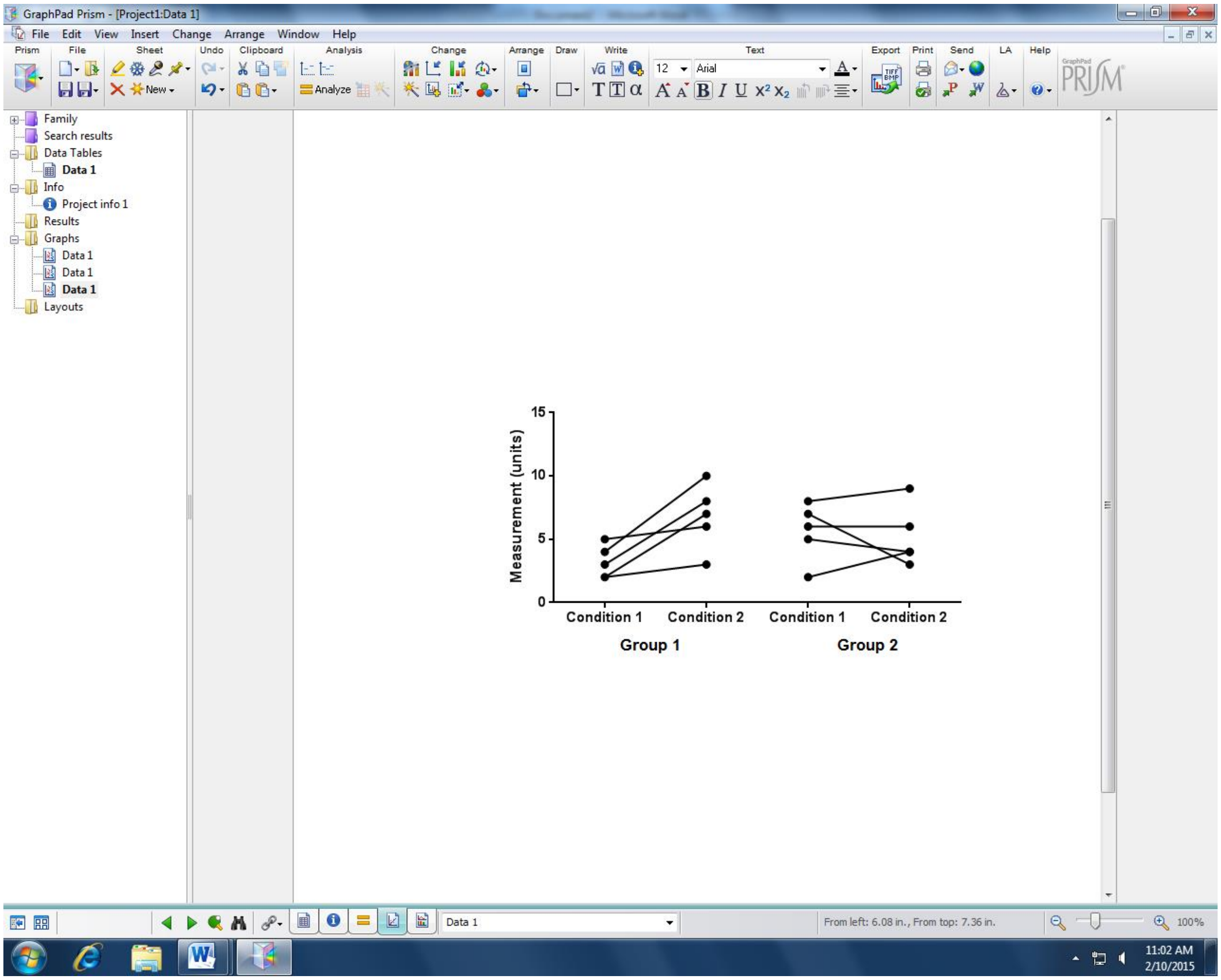

Downloaded from: Weissgerber TL, Milic NM, Winham SJ, Garovic VD. Beyond Bar and Line Graphs: Time for a New Data Presentation Paradigm. *PLOS Biology*, 2015:13.

13. Select “File”, then “Export” to save the graph.

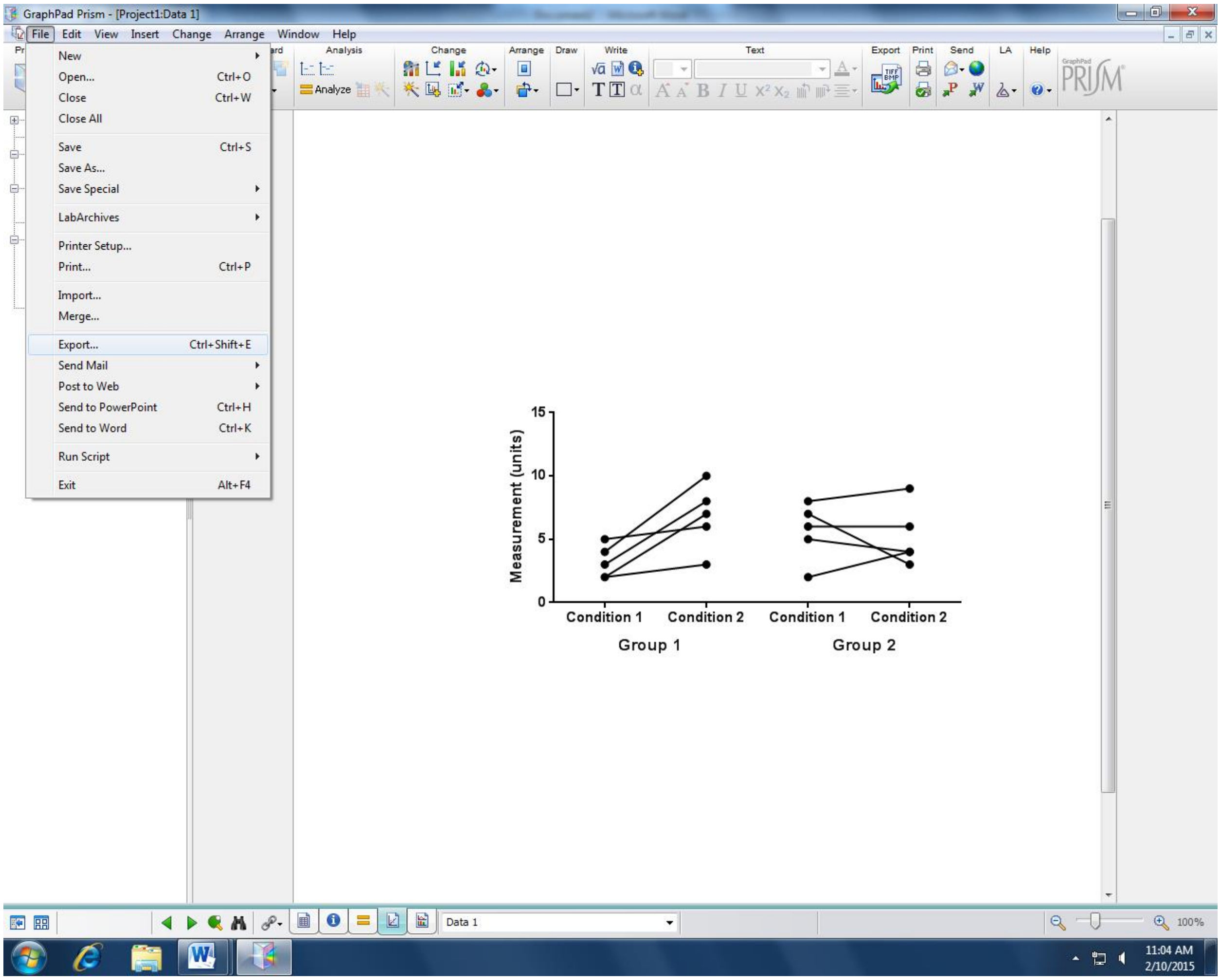

Downloaded from: Weissgerber TL, Milic NM, Winham SJ, Garovic VD. Beyond Bar and Line Graphs: Time for a New Data Presentation Paradigm. *PLOS Biology*, 2015:13.

14. Select the file format, enter a file name and chose the location where you want to save the file. Click “OK”. Your graph should be saved in the format and location that you selected.

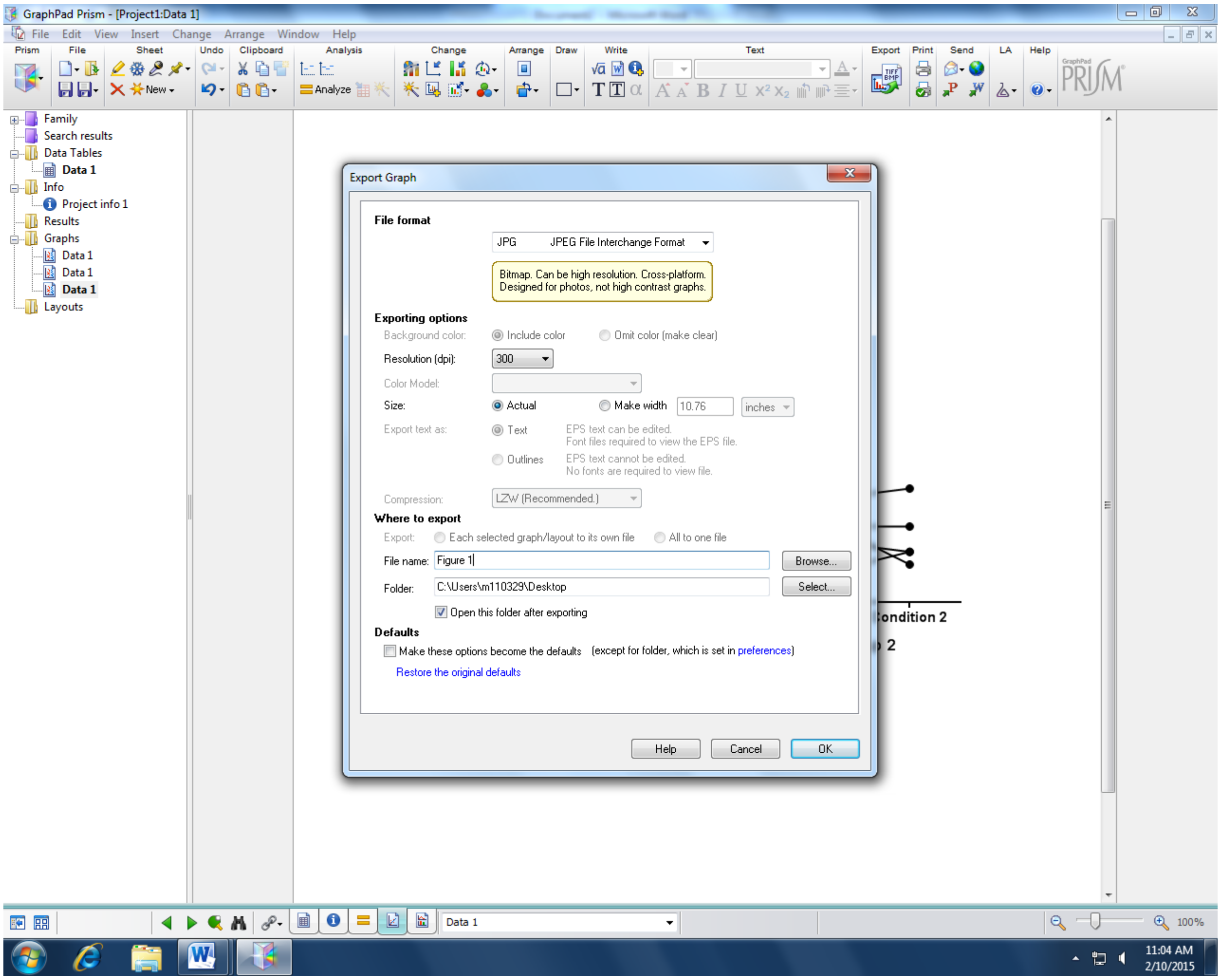

Downloaded from: Weissgerber TL, Milic NM, Winham SJ, Garovic VD. Beyond Bar and Line Graphs: Time for a New Data Presentation Paradigm. *PLOS Biology*, 2015:13.

15. The next step is to graph the differences for both groups. Enter the name for Group 1 just below “Group E”. Enter the name for Group 2 just below “Group F”. The condition names that you enter will appear as labels on your graph.
    Enter you data as follows (one row per subject or specimen):
    - “Group E” column: Difference scores for Group 1 (Condition 2 – Condition 1)
    - “Group F” column: Difference scores for Group 2 (Condition 2 – Condition 1)

    Subjects from Group 1 will only have a difference score in the “Group E” column, whereas subjects from Group 2 will only have a difference score in the “Group F” column.

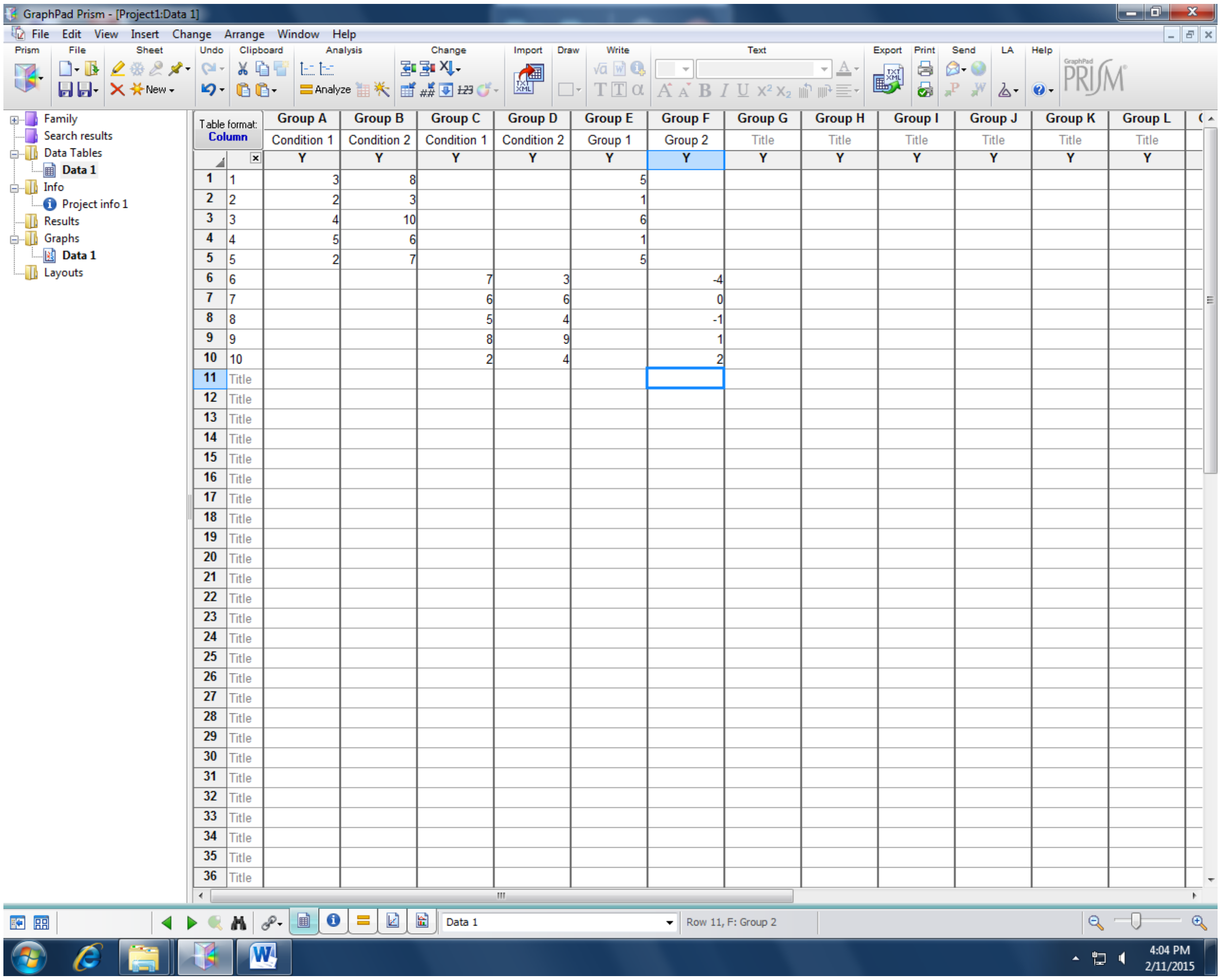

Downloaded from: Weissgerber TL, Milic NM, Winham SJ, Garovic VD. Beyond Bar and Line Graphs: Time for a New Data Presentation Paradigm. *PLOS Biology*, 2015:13.

16. From the insert menu at the top of the screen, select “New graph of existing data”

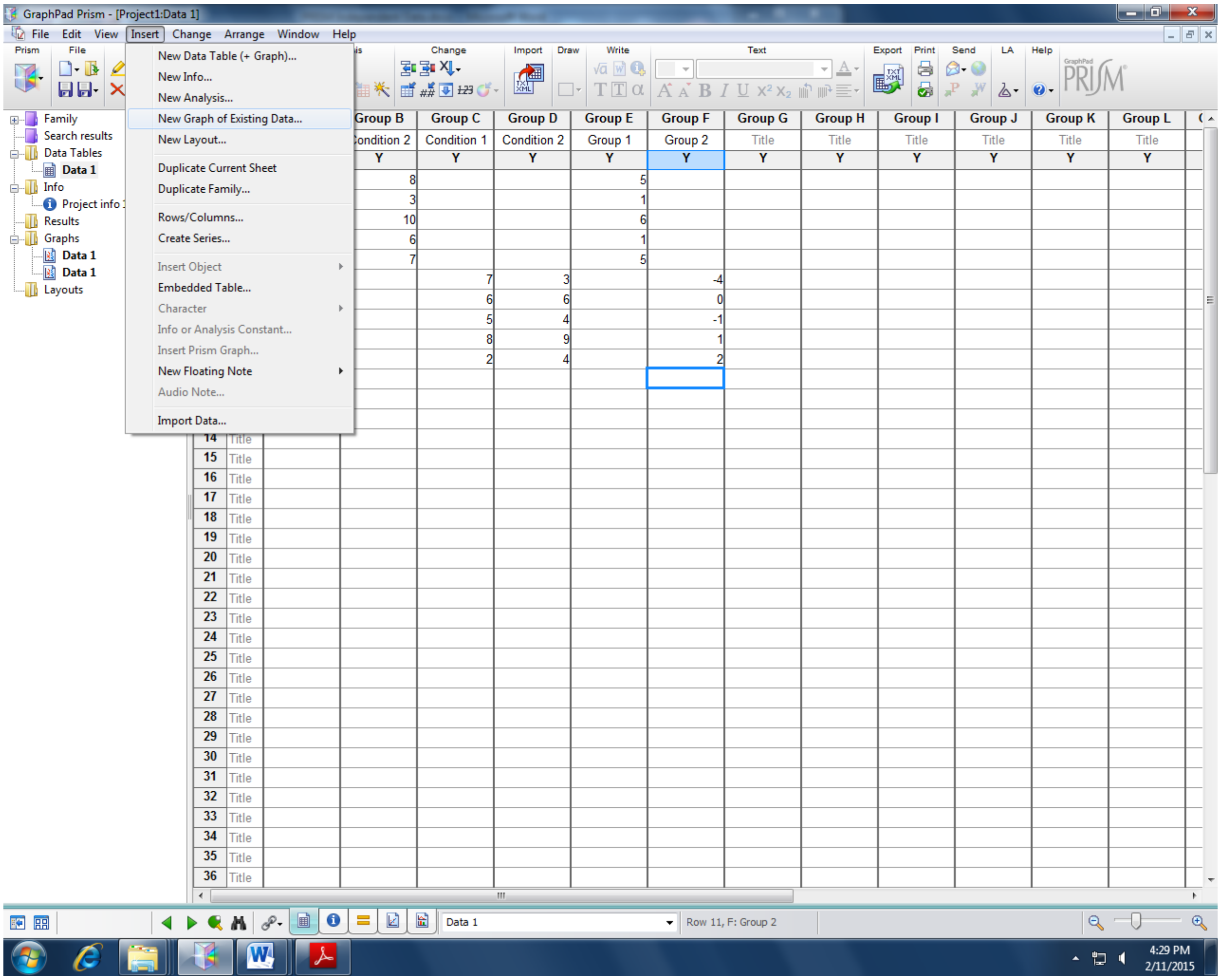

Downloaded from: Weissgerber TL, Milic NM, Winham SJ, Garovic VD. Beyond Bar and Line Graphs: Time for a New Data Presentation Paradigm. *PLOS Biology*, 2015:13.

17. Select "scatterplot" in the pop-up menu. Under the plot menu, select any summary statistics that you would like the graph to show. You should show the mean if you are using parametric statistics for your data analysis, and the median if you are using non-parametric statistics for your data analysis. Click "OK".

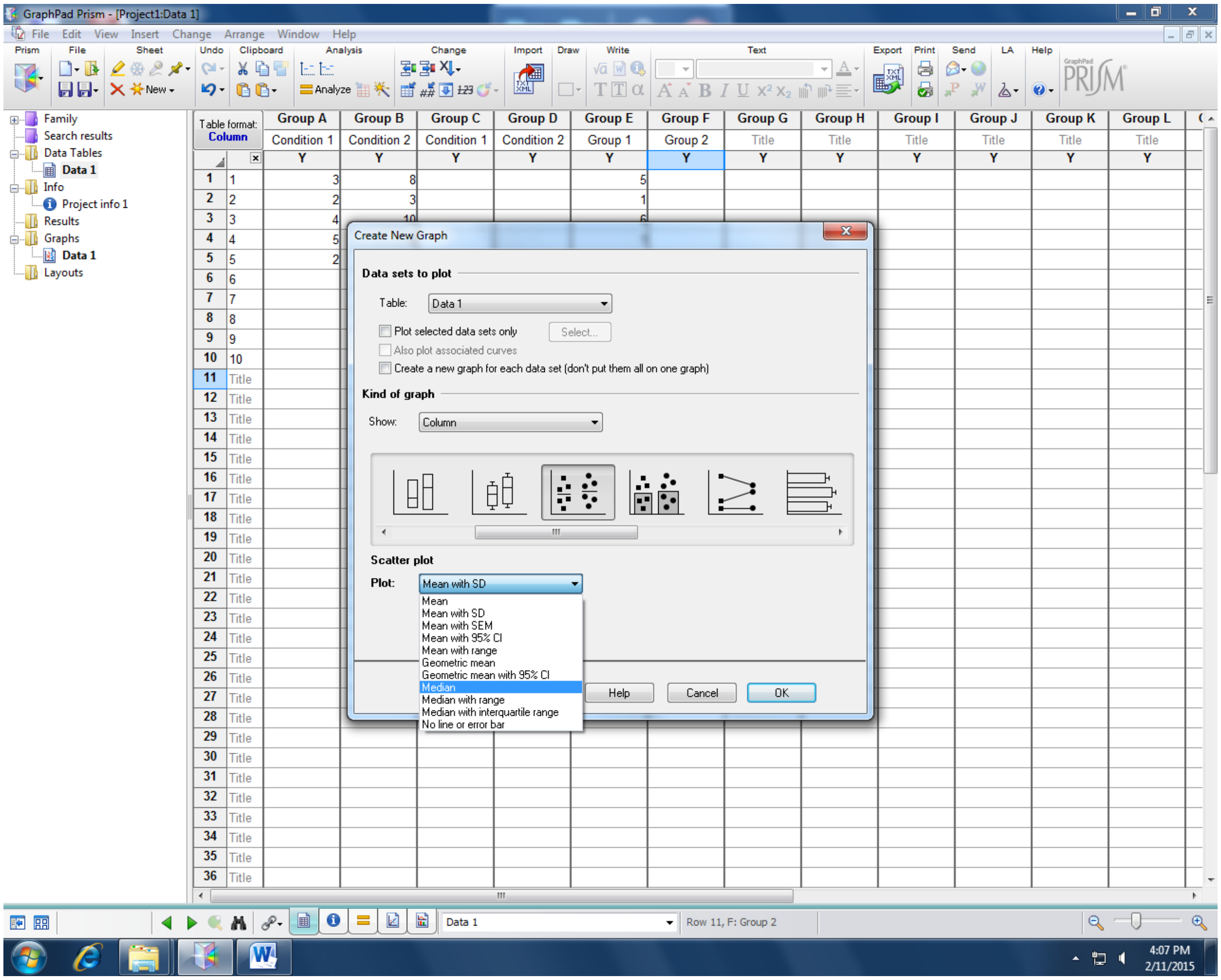

Downloaded from: Weissgerber TL, Milic NM, Winham SJ, Garovic VD. Beyond Bar and Line Graphs: Time for a New Data Presentation Paradigm. *PLOS Biology*, 2015:13.

18. The graph will look something like this. You will need to delete the groups that are not difference scores. In the “Change” menu at the top of the screen, click on the “Format Graph” symbol.

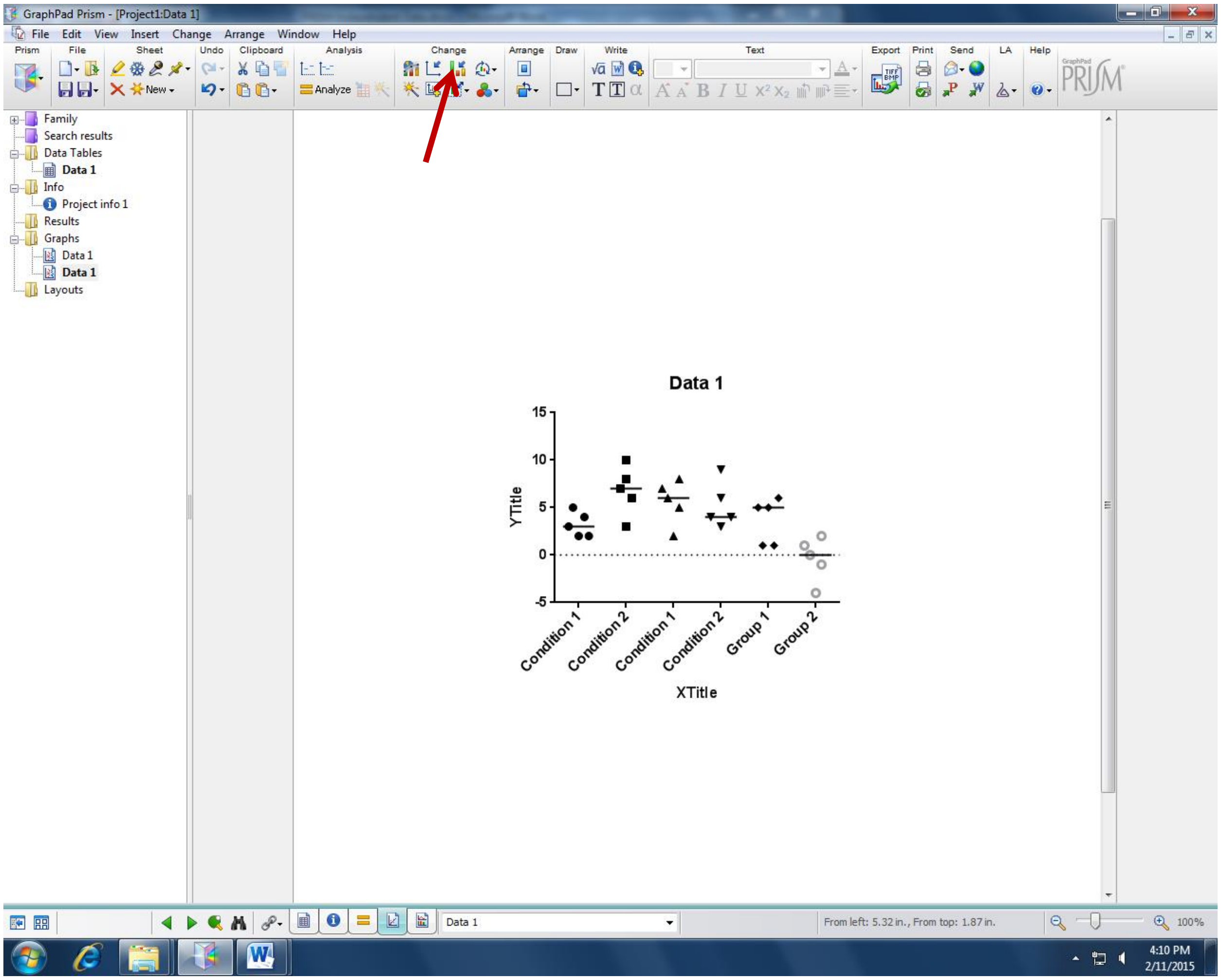

Downloaded from: Weissgerber TL, Milic NM, Winham SJ, Garovic VD. Beyond Bar and Line Graphs: Time for a New Data Presentation Paradigm. *PLOS Biology*, 2015:13.

19. Go to the “Data Sets on Graph” tab in the pop-up menu. Highlight the groups that contain raw data for each condition. Click “Remove”, then click “OK”.

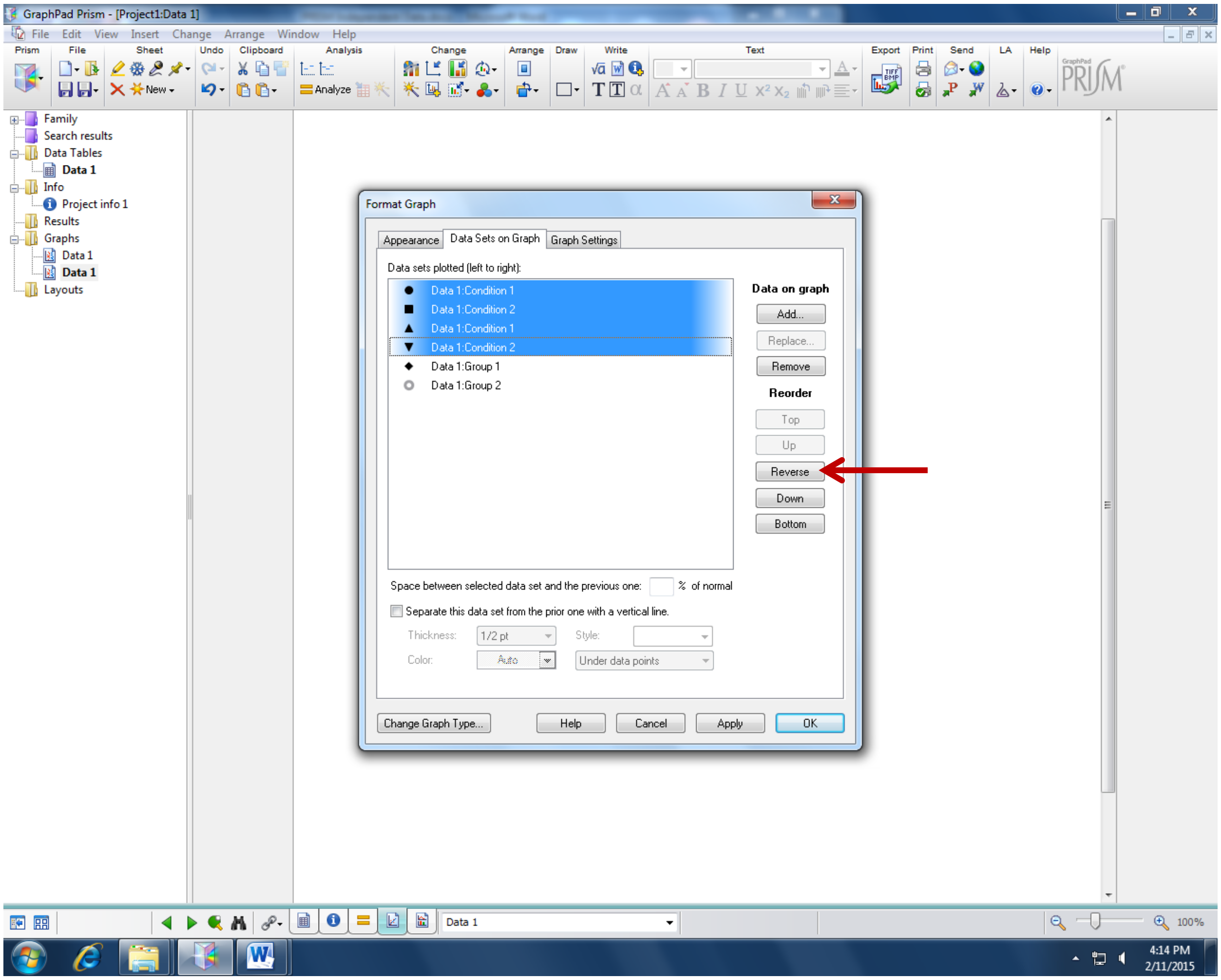

Downloaded from: Weissgerber TL, Milic NM, Winham SJ, Garovic VD. Beyond Bar and Line Graphs: Time for a New Data Presentation Paradigm. *PLOS Biology*, 2015:13.

20. The graph will look something like this. Click on “YTitle” to replace this label with the names and units for your axis. Either delete the chart title (Data1 in this example) and “XTitle” or click on these titles to replace them with the desired text.

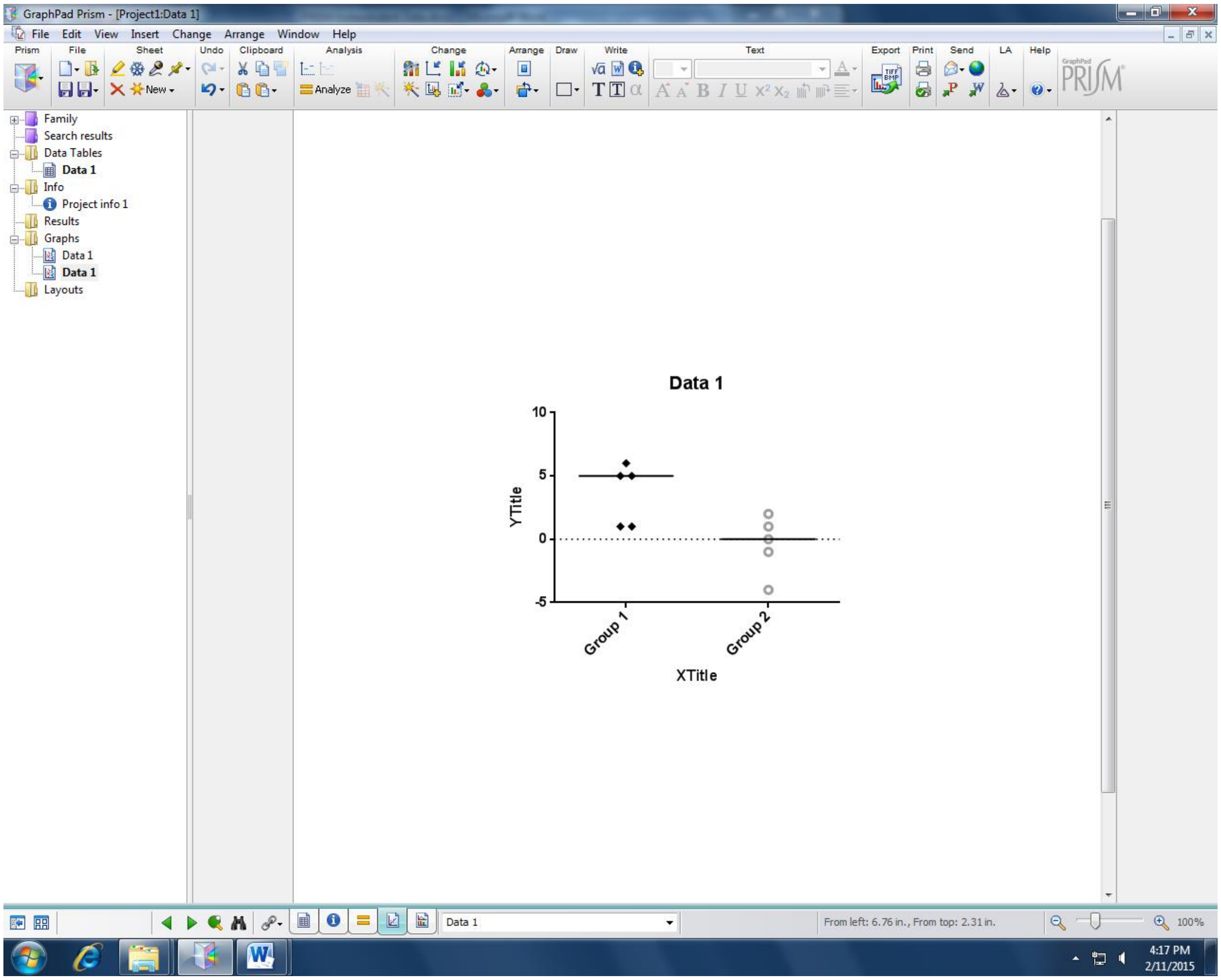

Downloaded from: Weissgerber TL, Milic NM, Winham SJ, Garovic VD. Beyond Bar and Line Graphs: Time for a New Data Presentation Paradigm. *PLOS Biology*, 2015:13.

21. Your new axis labels should now appear on the scatterplot. Left click on one of the x-axis labels (Group 1, Group 2, etc.); then right click and select “Format axis”.

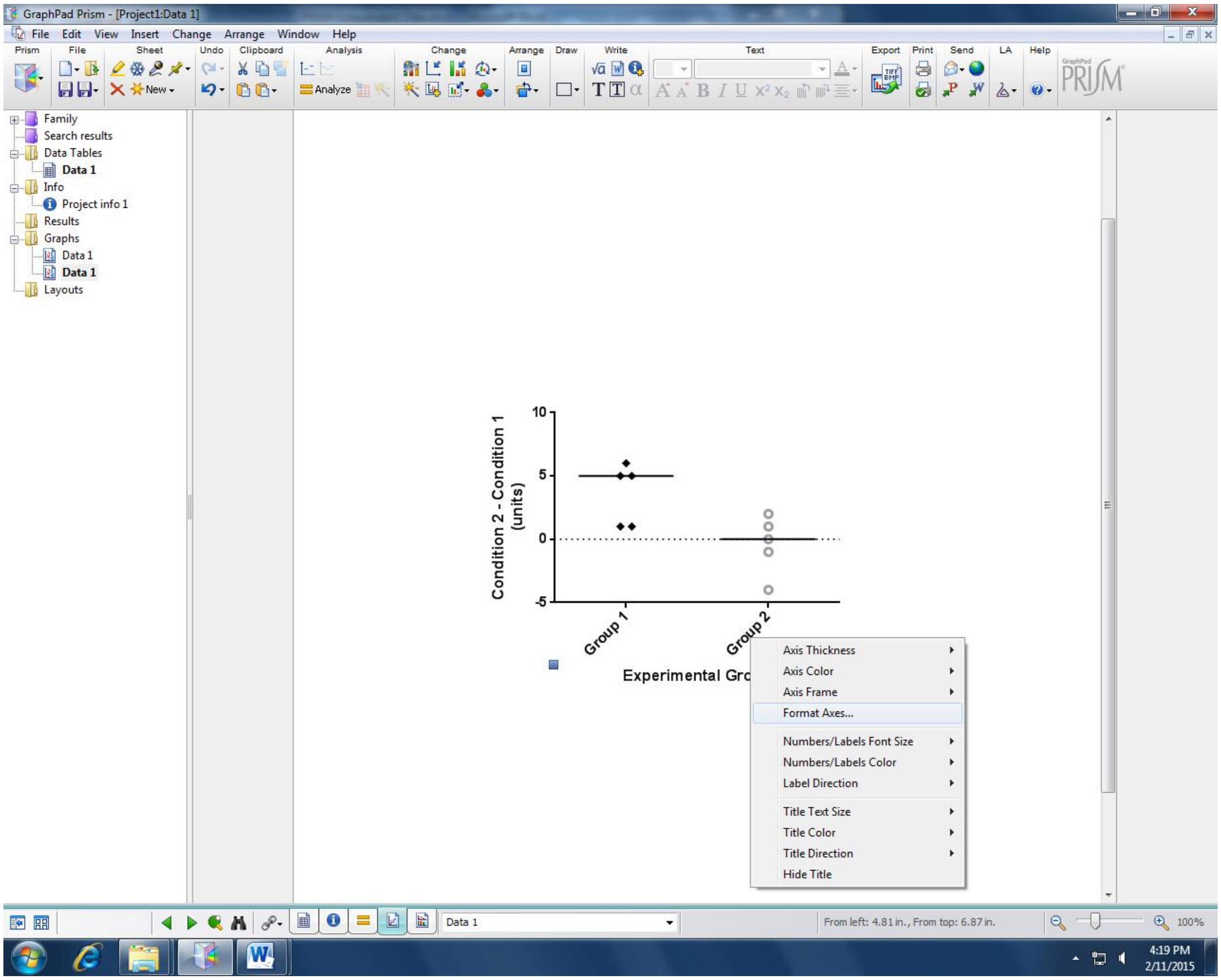

Downloaded from: Weissgerber TL, Milic NM, Winham SJ, Garovic VD. Beyond Bar and Line Graphs: Time for a New Data Presentation Paradigm. *PLOS Biology*, 2015:13.

22. In the pop-up menu, under the x-axis tab change “Below, angled” to “Below, horizontal”.

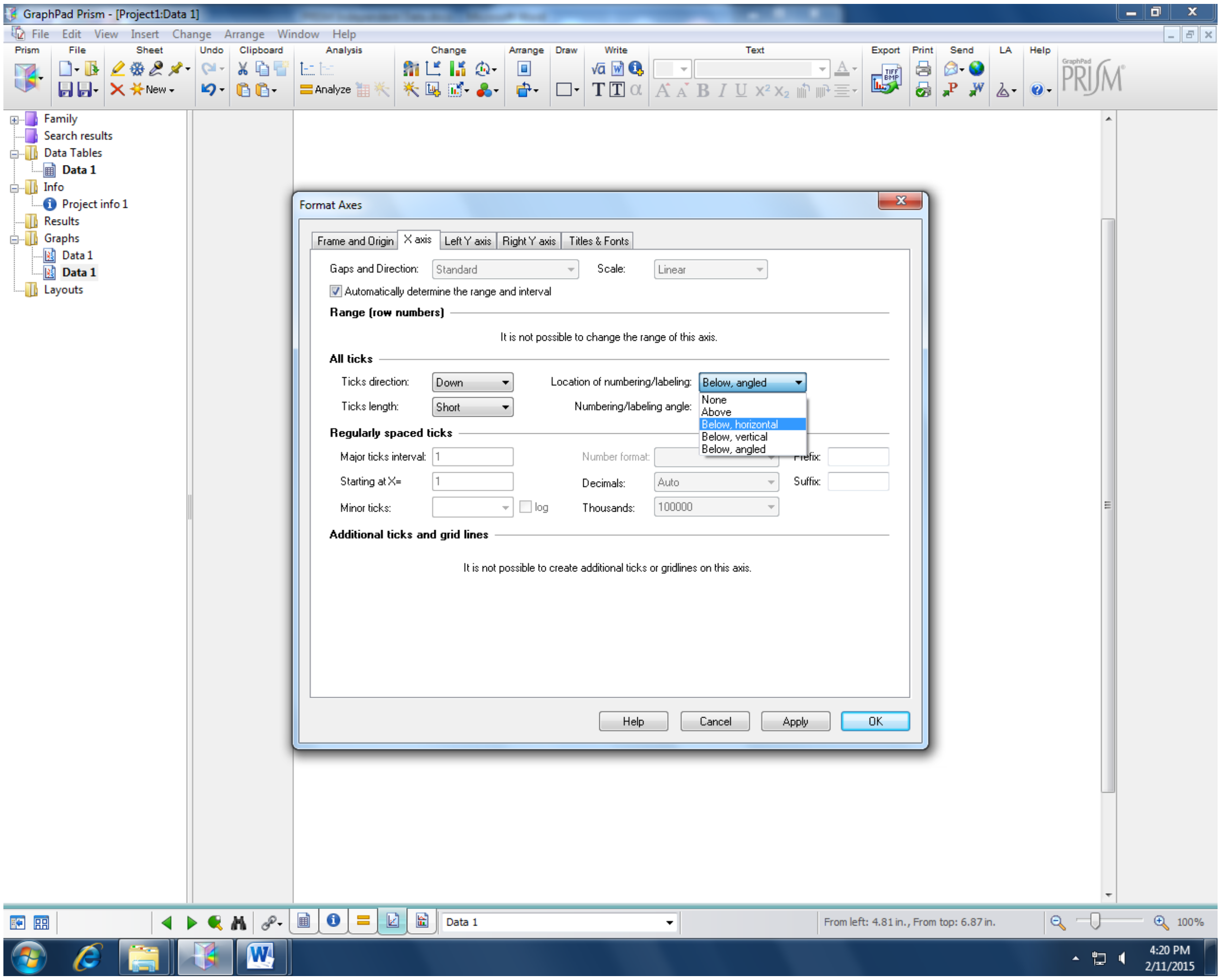

Downloaded from: Weissgerber TL, Milic NM, Winham SJ, Garovic VD. Beyond Bar and Line Graphs: Time for a New Data Presentation Paradigm. *PLOS Biology*, 2015:13.

23. If you are using an x-axis title, select the “Titles & Fonts” tab. Change ”Distance from axis” for the x-axis title to the desired value. Click “Apply” to view the change. Click “OK” when you are happy with the result.

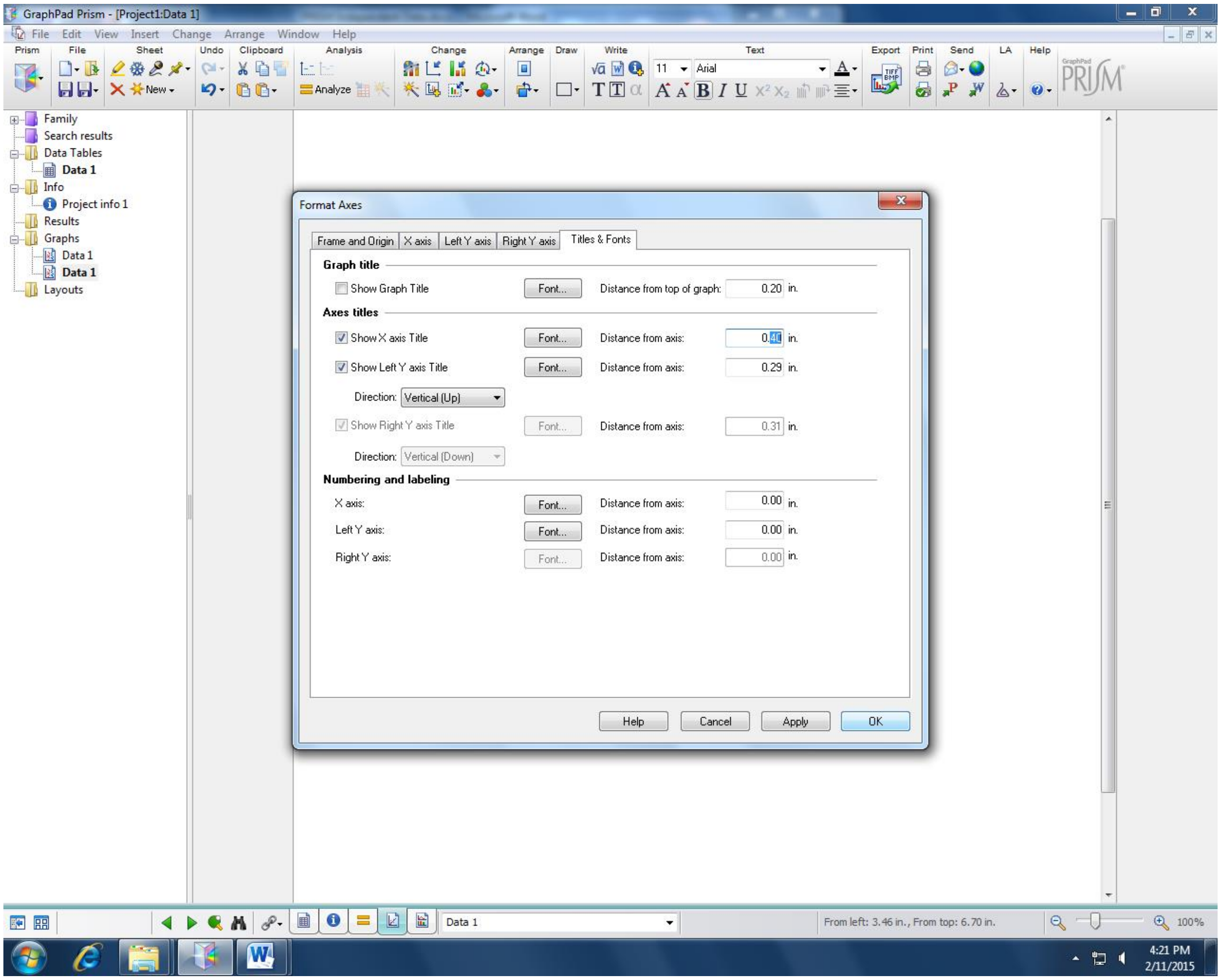

Downloaded from: Weissgerber TL, Milic NM, Winham SJ, Garovic VD. Beyond Bar and Line Graphs: Time for a New Data Presentation Paradigm. *PLOS Biology*, 2015:13.

24. Your univariate scatterplot will look something like this. Select “File”, then “Export” to save the scatterplot.

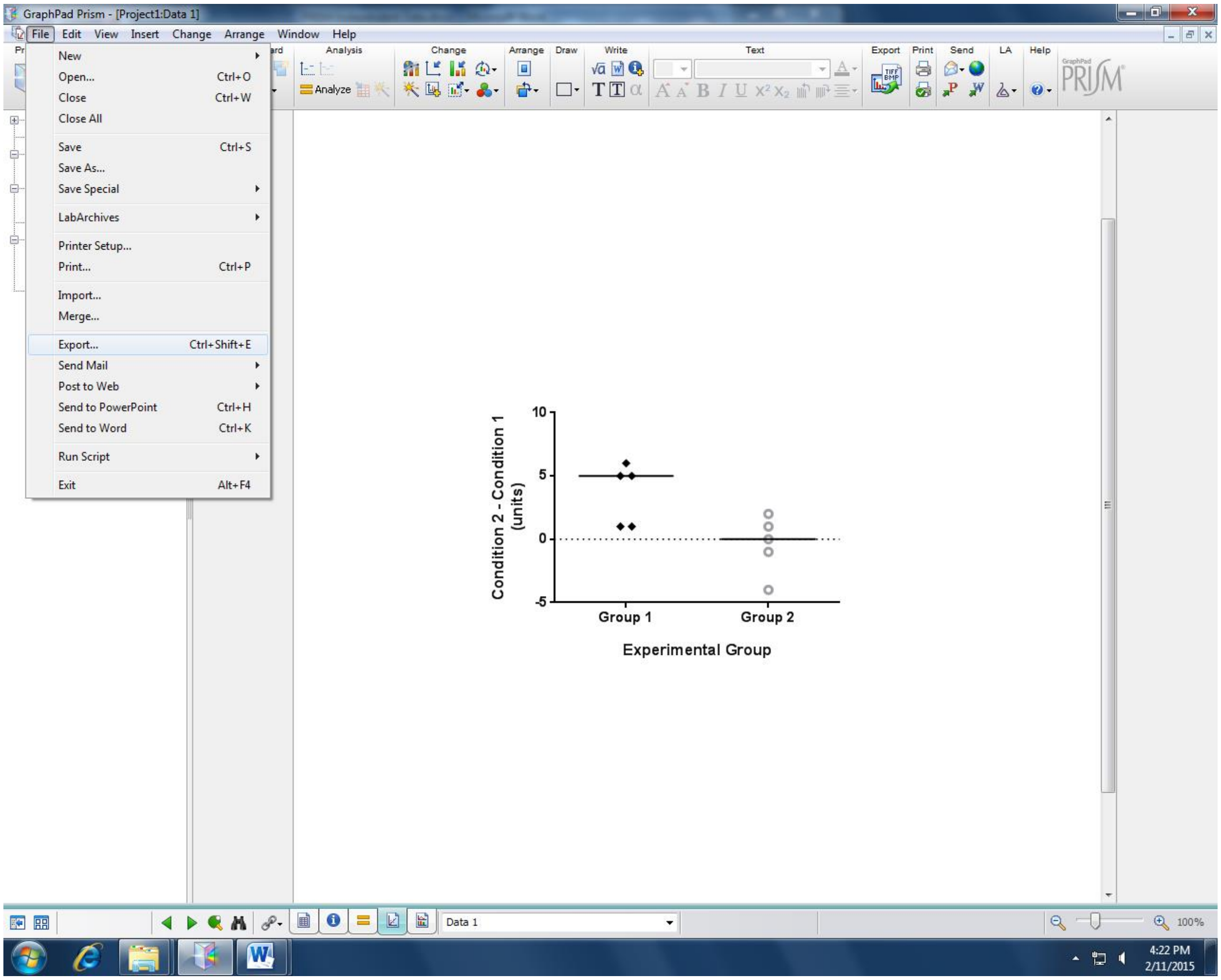

Downloaded from: Weissgerber TL, Milic NM, Winham SJ, Garovic VD. Beyond Bar and Line Graphs: Time for a New Data Presentation Paradigm. *PLOS Biology*, 2015:13.

25. Select the file format, enter a file name and chose the location where you want to save the graph. Click “OK”. Your scatterplot will be saved in the format and location that you selected.

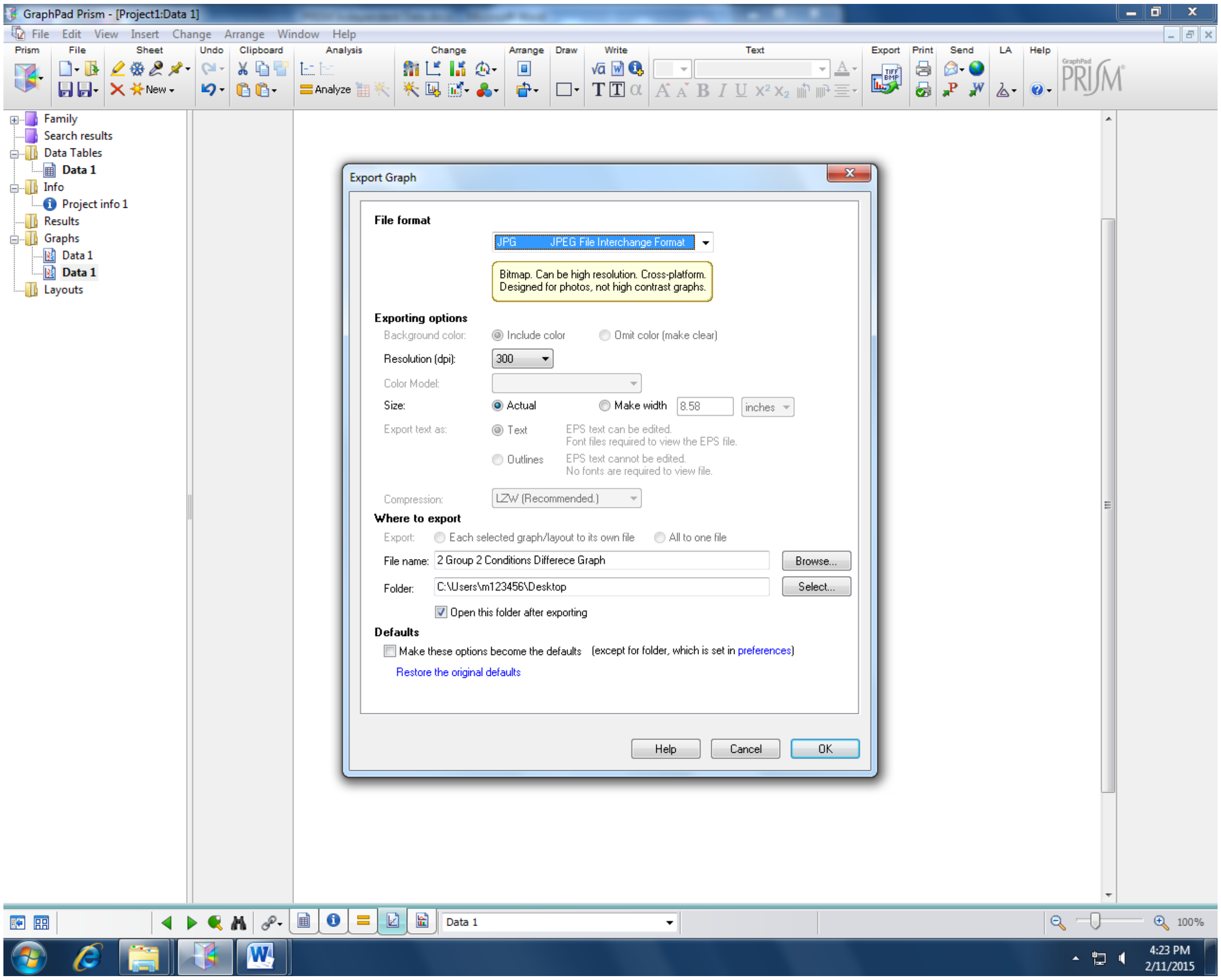

Downloaded from: Weissgerber TL, Milic NM, Winham SJ, Garovic VD. Beyond Bar and Line Graphs: Time for a New Data Presentation Paradigm. *PLOS Biology*, 2015:13.
