# Supplementary figures and images for: Beyond Bar and Line Graphs: Time for a New Data Presentation Paradigm
Source: PLoS Biol. 2015 Apr 22;13(4):e1002128. doi: 10.1371/journal.pbio.1002128 (PMC4406565; doi:10.1371/journal.pbio.1002128)

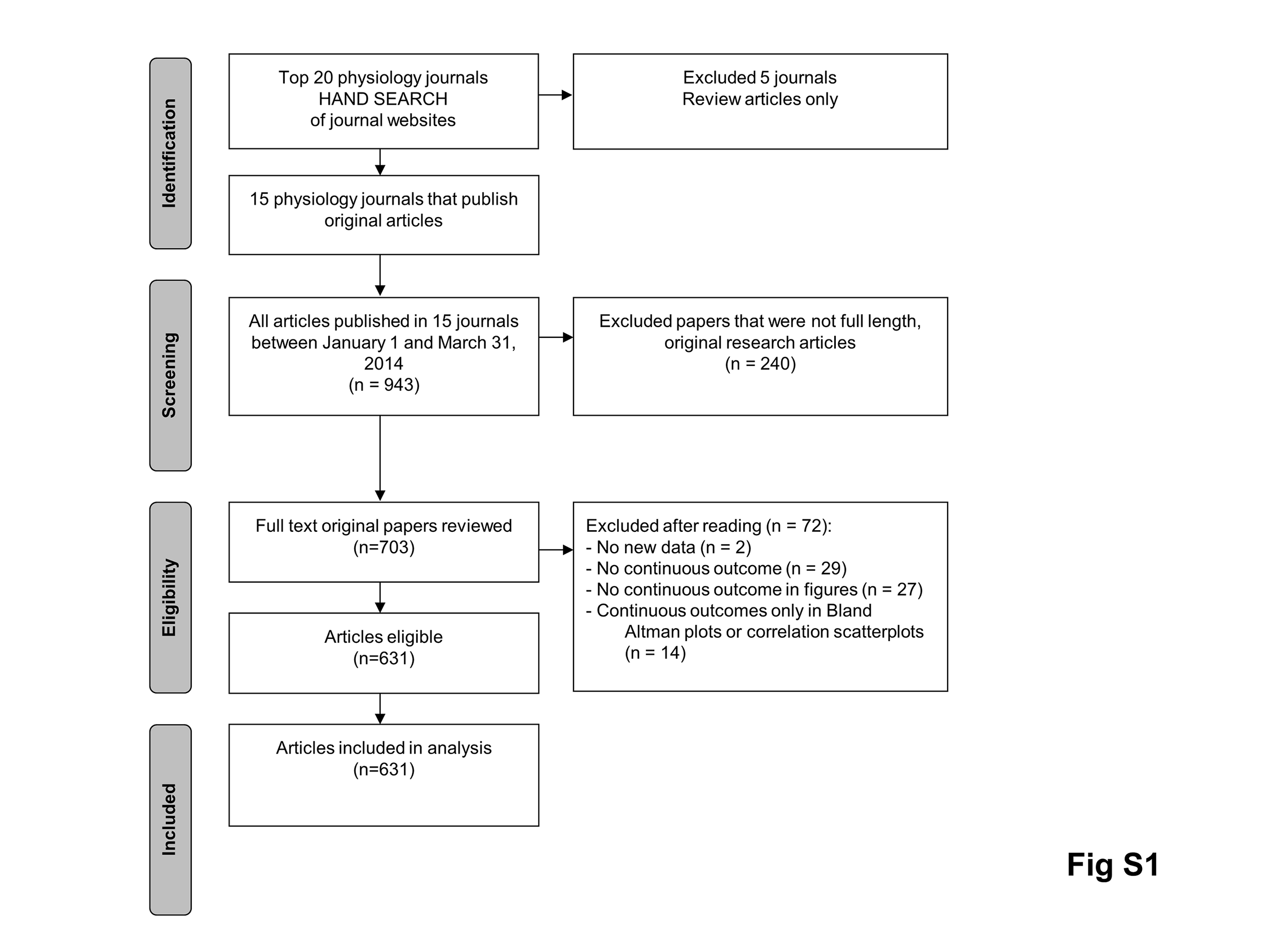

Supplement: S1 Fig — (TIF) [file pbio.1002128.s007.TIF]

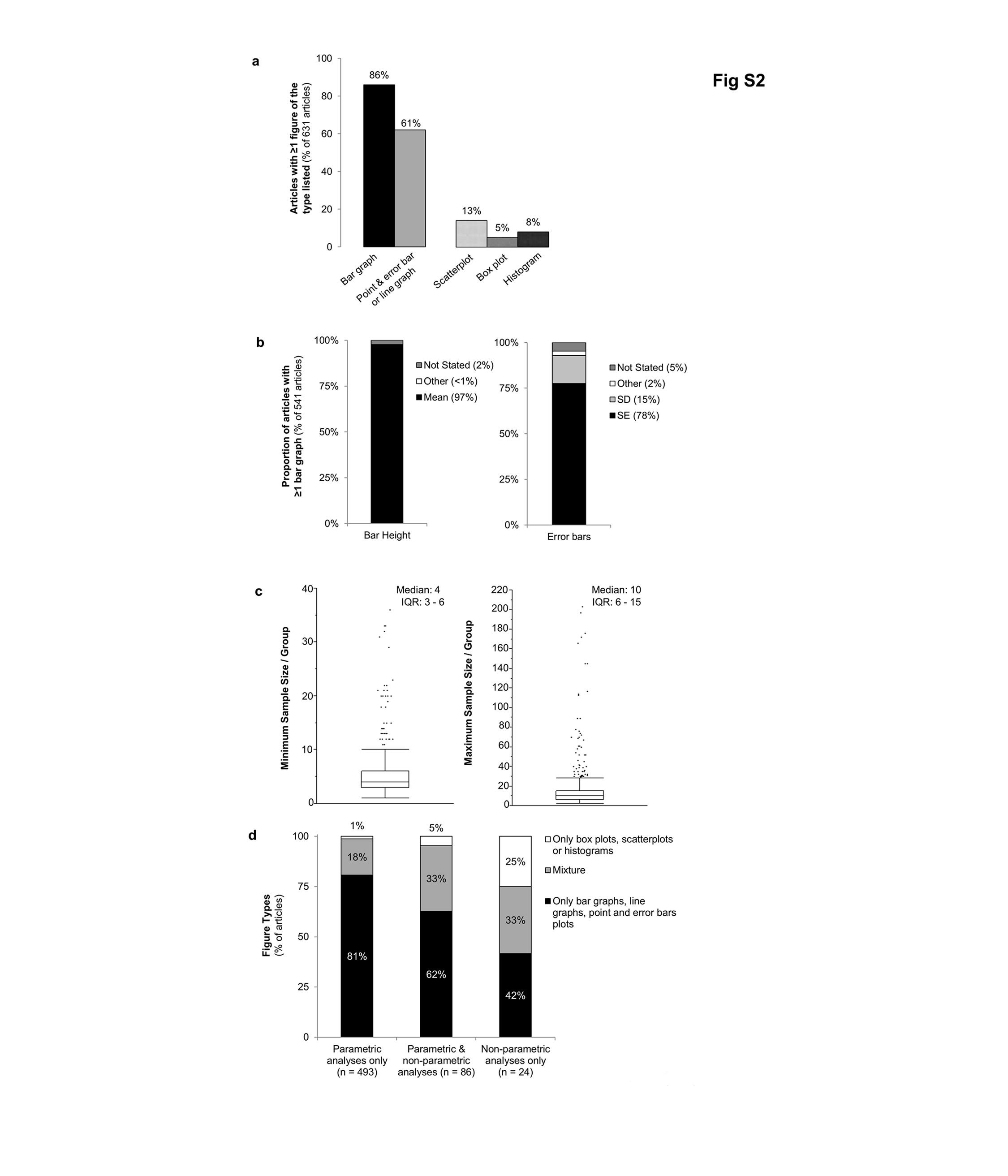

Supplement: S2 Fig — Panel a: Bar graphs and other figures that typically show mean and SE or mean and SD were strongly preferred to figures that provide detailed information about the distribution of the data (scatterplots, box plots, and histograms). Panel b: Most bar graphs show mean ± SE. Panel c: Box plots show the minimum and maximum sample sizes for any group presented in a figure. The box shows the median and interquartile range. Whiskers show the furthest point that is within 1.5 times the interquartile range. Note that a few very high outliers are not shown (n = 8 for minimum sample size; n = 7 for maximum sample size). The maximum values for minimum and maximum sample size per group were 593 and 2,192, respectively. Showing these outliers would make the box plots impossible to see. Seventeen studies were excluded from this analysis as sample size was not reported (n = 614). Panel d: The types of figures that are selected depend on the type of statistical analysis that is performed. We performed ordinal logistic regression, with analysis type and figure type both classified as ordinal variables. The distribution of figure types differed significantly between studies that performed only parametric analyses and studies that performed both parametric and nonparametric analyses (p < 0.001), and between studies that performed both types of analyses and studies that performed only nonparametric analyses (p < 0.001). (TIF) [file pbio.1002128.s008.TIF]
